# Synthesis of New Brassinosteroid Analogs with Androstane Skeleton and Heterocyclic Acyl Side Chains: Preliminary Molecular Docking Studies

**DOI:** 10.3390/molecules30194011

**Published:** 2025-10-07

**Authors:** Omara Araya, María Núñez, Marco Mellado, Andrés F. Olea, Luis Espinoza-Catalán

**Affiliations:** 1 Departamento de Química, Universidad Técnica Federico Santa María, Avenida España 1680, Valparaíso 2340000, Chile; omara.araya@usm.cl; 2 Centro de Investigación en Ingeniería de Materiales, Universidad Central de Chile, Santiago 8330507, Chile; marco.mellado@ucentral.cl; 3 Instituto de Ciencias Aplicadas, Facultad de Ingeniería, Universidad Autónoma de Chile, Av. del Valle Sur 534, Santiago 8580640, Chile; andres.olea@uautonoma.cl

**Keywords:** brassinosteroids, androstane, heterocyclic side chain

## Abstract

Brassinosteroid analogs with heterocyclic rings in the side chain are interesting because important biological activity has been shown by these compounds. Thus, herein, five 23-24-dinorcholane BR analogs with a heterocyclic ester function at C-22 were synthesized and fully characterized by different spectroscopic techniques. The acylation reaction at C-22, which is a key synthetic step, was carried out by two different methods, namely acylation with heterocyclic acid chlorides and Steglich esterification reaction. In both cases, the acyl derivatives were obtained with good yields. Additionally, a preliminary molecular docking study of BRI1–BAK1 complexes formed by these analogs and brassinolide was performed to estimate what their biological activity would be. Results indicate that the complex formed by the analog **36**, which has an indole group in the side chain, within the active site of BRI1–BAK1 is more stable than that formed by brassinolide. Additionally, molecular docking of a derivative having a benzoate function at C-22 and a F atom in the *ortho* position, **23**, shows a similar pose and interactions at the active site but the highest binding energy. As **23** has shown similar activity to brassinolide in the Rice Lamina Inclination Test, it is expected that **36** will also exhibit similar behavior.

## 1. Introduction

**Figure 1:**
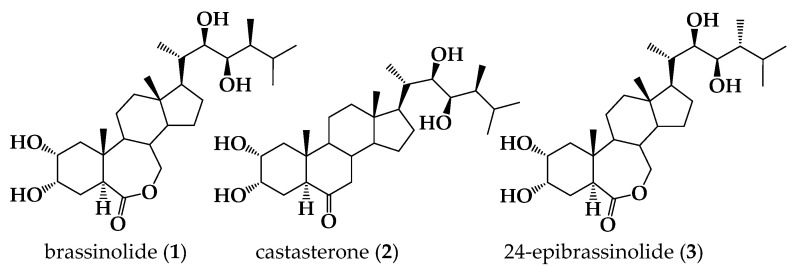
Brassinolide (**1**) and other important naturally occurring BRs, castasterone (**2**) and 24-epibrassinolide (**3**).

Brassinosteroids (BRs) are a group of phytohormones that are widespread in the plant kingdom, especially in angiosperms [1]. Brassinolide (**1**) was the first compound of this group to be isolated and characterized. After that, castasterone (**2**), epibrassinolide (**3**), and more than 80 naturally occurring BRs were reported [2]. The presence of BRs influences the development and growth of plants [3,4], even under conditions of abiotic and biotic stress [5,6,7,8,9,10,11,12]. Due to their highest biological activity and ubiquitous distribution in plants, **1**, **2**, and 24-epibrassinolide (**3**) (Figure 1) have been extensively used in studies of the physiological effects of exogenous BR application [13].

Even though BRs are found in roots, stems, leaves, flowers, anthers, pollen, seeds, and grain [14,15,16,17], their concentration is extremely low, 1–100 ng/g fresh weight in young tissues and 0.01–0.1 ng/g fresh weight in mature ones [18]. Thus, much effort has been dedicated to developing efficient synthesis methods for **1** and BR derivatives. However, the chemical structures of BRs are extremely complex, as shown by a significant number of chiral centers and functional groups. Although several attempts were made, their total synthesis seems difficult and impractical [19,20]. Partial syntheses using other abundant steroids as starting substances, with organic functions in appropriate positions (easy and convenient to be chemically modified), have been explored to obtain BR derivatives in amounts that allow both scientific and practical studies to be performed. Several reviews focus on stereoselective synthesis leading to functionalized A, B rings in the steroid system, and also on the side chain modification of naturally occurring BRs and their synthetic analogs [21,22,23,24].

**Figure 2:**
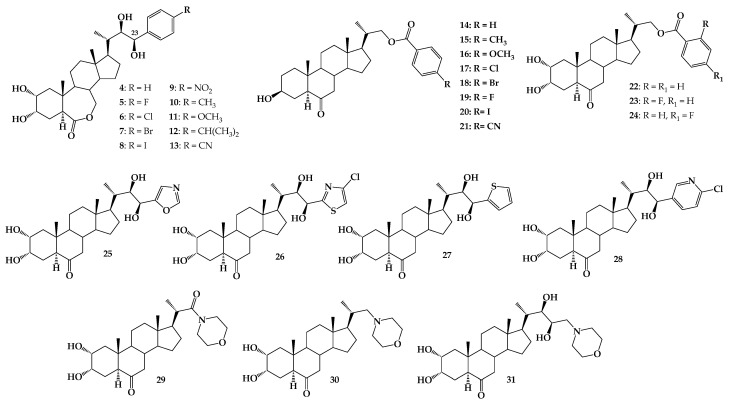
Structure of synthetic BR analogs with an aryl ring at C-23 (**4**–**13**), 23,24-dinorcholan type analogs with 3β-hydroxyl and 22-benzoate having a different substituent at the ˝*para*” position on aromatic ring (**14**–**21**), 2α,3α-dihydroxy with 4-substituted benzoate at C-22 (compounds **22**–**24**), analogs with a heteroaryl group at C-23 with different sizes (compounds **25**–**28**), and analogs with heterocyclic nitrogen-containing side chains (**29**–**31**).

In the last few decades, much effort has been made to synthesize BR analogs, in which the side chain is submitted to huge changes in length, nature of oxygenated functional groups, and attachment of substituted cyclic or aromatic rings, esters, or carboxylic acids. Surprisingly, it has been found that these analogs exhibit interesting biological activity. For example, it has been reported that exchange of the terminal isopropyl group for cyclopropyl or cyclobutyl induces a five-to-seven-fold increase in activity in the Rice Lamina Inclination Test (RLIT) [25]. Similarly, exchanging the alkyl chain for an aromatic ring has also been carried out, and 23-phenyl BR derivatives (compounds **4**–**13**, Figure 2) were synthesized. Their RLIT activity was reported to be like that shown by 24-epibrassinolide [26,27,28]. Additional BR analogs with a substituted aryl group at C-23 have been studied to assess the effect of substituent chemical structure on biological activity [27,28].

On the other hand, the synthesis of 23,24-dinorcholane benzoates at C-22, with electron-donating and electron-withdrawing substituents in the “*para*” position of aromatic ring (compounds **14**–**24**, Figure 2), has also been reported [29,30]. Evaluation of their biological activities has been performed by different bioassays, i.e., RLIT, Bean Second Internode (BSI), and *Arabidopsis* brassinosteroid sensitivity assay. Results suggest that electron-withdrawing and size effects seem to be the most important factors in determining activities of these analogs [29,30].

Following this line, the synthesis and biological evaluation of analogs in which heteroaryl groups, with different sizes and electronic properties, have been attached to C-23 (compounds **25**–**28**, Figure 2) have been performed [7]. Results indicate that thiophene derivative **27** is among the most active five-ring analogs, whereas the corresponding more polar oxazole **25** exhibits only moderate activity.

Interestingly, an analysis of the receptor binding site of analogs **25**–**28** (molecular docking study) revealed mostly lipophilic interactions with Ile 563, Met 657, and Trp 564 and probably some free space available for side chain modification, which is in agreement with the good activity observed for such modified brassinosteroid analogs [7].

In addition, BR analogs with heterocyclic nitrogen-containing side chains have also been reported (compounds **29**–**31**, Figure 2) [31]. The bioactivity of these compounds was tested by an *Arabidopsis* root sensitivity bioassay and cytotoxicity screening. Finally, a few additional studies on the synthesis and biological activities of BR analogs with heterocyclic rings in the side chain have been reported [7,31].

Considering the potential application of BR analogs with side chains substituted with aromatic heterocyclic systems with varying sizes and electron distributions (compounds **25**–**28**), or with aliphatic heterocyclic rings containing nitrogen atoms (compounds **29**–**31**), we consider that it is interesting to explore the synthesis of new analogs containing different heterocyclic systems with variations in size and electron distribution. So, herein, the synthesis of a new series of 23,24-dinorcholane BR analogs with a heterocyclic ester function at C-22 is reported.

Additionally, all synthesized compounds were considered in a preliminary molecular docking study in the BRI1–BAK1 crystallized complex with the aim of obtaining their theoretical biological activity. Interestingly, one of them gives a binding energy lower than that obtained for brassinolide (**1**). Thus, a comparative study of the biological activities of BR analogs carrying an acyl heterocyclic function in the side chain structure is underway.

## 2. Results and Discussion

**Figure 3:**
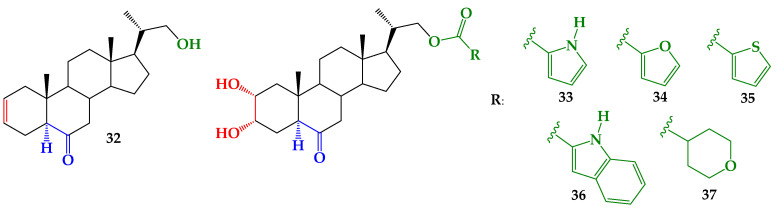
Chemical structures of precursor **32**, and the new BR analogs **33**–**37** with a heterocyclic ester function at C-22.

The structures of new analogs synthesized in this work, **33**–**37**, are shown in Figure 3. These compounds have been obtained using the known compound **32** as a starting material [29].

### 2.1. Chemical Synthesis

**Scheme 1:**
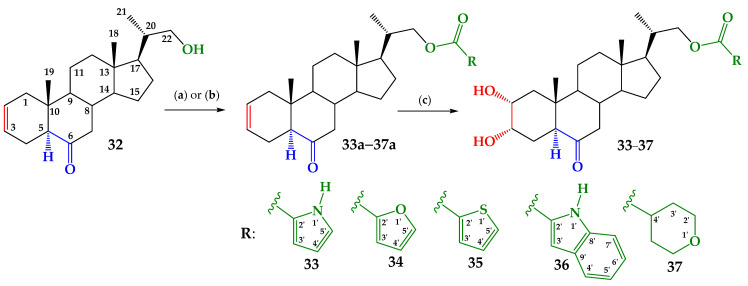
Conditions: (**a**) Het-COCl, DMAP/py/CH_2_Cl_2_; r.t. 2 h; **34a**, **35a**, and **37a** at 70.0%, 52.5%, and 75.5% yield, respectively. (**b**) Het-CO_2_H, DCC, DMAP/C_6_H_6_, r.t. 24 h, **33a** and **36a** at 73.1% and 71.2% yield, respectively. (**c**) Sharpless dihydroxylation: DHQD-CLB/CH_3_SO_2_NH_2_, K_2_CO_3_/K_3_[Fe(CN)_6_], t-BuOH/H_2_O (1:1 *v*/*v*), and OsO_4_/t-BuOH; 36 h; **33**–**37** at 83.3%, 57.6%, 66.3%, 37.1%, and 71.4% yield, respectively.

Compound **32**, obtained and described previously [29], is esterified by two different methods, depending on the availability and reactivity of the corresponding acid chlorides or carboxylic heterocyclic acids (Scheme 1). On one hand, compounds **34a**, **35a**, and **37a** are prepared by esterification with the corresponding heterocyclic acid chlorides (furan-2-carbonyl chloride, thiophene-2-carbonyl chloride, and tetrahydro-2*H*-pyran-4-carbonyl chloride, respectively). These reactions are performed in a Py/DMAP/CH_2_Cl_2_ system according to a protocol reported for other benzoyl derivatives [29,30]. On the other hand, derivatives **33a** and **36a** are prepared by Steglich esterification [32,33], with the corresponding heterocyclic acid (1*H*-pyrrole-2-carboxylic acid and 1*H*-indole-2-carboxylic acid) in a DCC/DMAP/C_6_H_6_ system.

Full structures of all compounds are determined by IR, 1D and 2D NMR experiments, and HRMS. All spectroscopic information, including IR; ^1^H, ^13^C{^1^H}, ^13^C{^1^H} DEPT-135, 2D ^1^H-^13^C HSQC, and 2D ^1^H-^13^C HMBC NMR; and HRMS spectra are shown in Figures S1–S56, Supplementary Material. To exemplify structural determination, the 2D ^1^H-^13^C HMBC spectrum obtained for compound **36a** is shown in Figure 4, where the most important heteronuclear correlations used for unambiguous structural determination of compound **36a** are shown.

**Figure 4:**
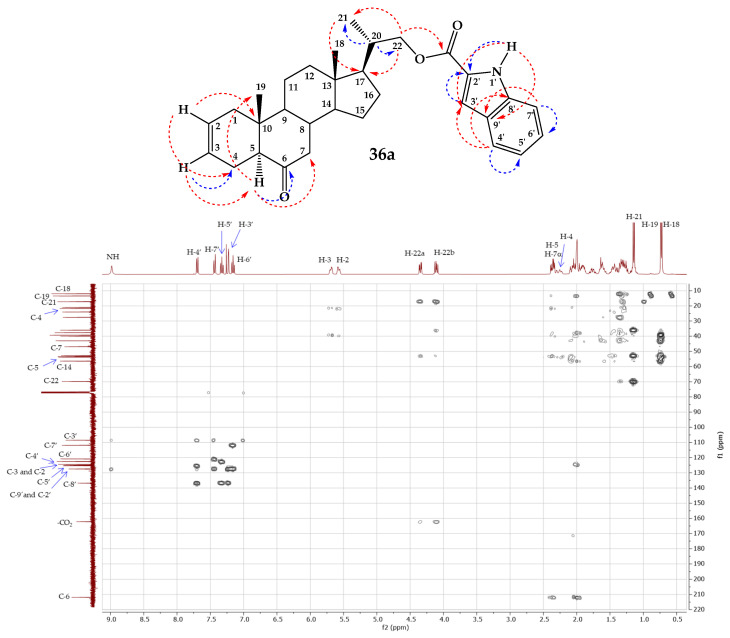
Top: structure of compound **36a**; bottom: 2D ^1^H-^13^C HMBC spectrum. Main ^2^*J*_HC_ (blue) and ^3^*J*_HC_ (red) correlations observed for the H-22 hydrogens of the side chain, indole moiety ring, and A/B junction rings.

In this way, the formation of ester function at C-22 is established by signals observed in the ^1^H NMR spectrum, namely at δ = 4.35 ppm (1H, dd, *J* = 10.7, and 3.5 Hz) and δ = 4.11 ppm (1H, dd, *J* = 10.7, and 7.2 Hz) which are assigned to the hydrogens H-22a and H-22b, respectively. These signals are correlated in the 2D HSQC spectrum with the signal observed at δ = 69.82 ppm (CH_2_-22 from ^13^C{^1^H} and ^13^C{^1^H} DEPT-135 spectra) (Figures S6–S10, Supplementary Material). Thus, formation of the ester function at C-22 is confirmed by the heteronuclear coupling signal at ^3^*J*_HC_ between the hydrogens H-22ab with the carbonyl group signal at δ = 162.19 ppm (^13^C{^1^H} NMR spectrum) observed in the 2D ^1^H-^13^C HMBC spectrum (Figure 4). Additionally, using the combined techniques of ^1^H, ^13^C{^1^H}, ^13^C{^1^H} DEP-135, 2D ^1^H-^13^C HSQC, and 2D ^1^H-^13^C HMBC NMR, it is possible to unequivocally identify and assign the signals corresponding to hydrogens NH, H-4’, H-7’, H-5’, H-3’, and H-6’ and C2’ to C9’ of the indole ring (Figure 4 and Figures S9 and S10, Supplementary Material). The rest of the heteronuclear correlations observed in the A/B rings and side chain confirm the structure of compound **36a**.

Subsequently, Sharpless dihydroxylation [27,29] of the double bond on C2 to C3 in compounds **33a**–**37a** produces the new BR analogs with the expected 2α,3α glycol function in compounds **33**–**37** with 83.3%, 57.6%, 66.3%, 37.1%, and 71.4% yield, respectively (Scheme 1). Determination of the chemical structure of these compounds is also established by combined IR, 1D, 2D NMR, and ^1^H-^13^C HRMS spectroscopic techniques (Figures S27–S56, Supplementary Material). For example, the 2D ^1^H-^13^C HMBC spectrum for analog **36** is shown in Figure 5.

The main heteronuclear correlations to ^2^*J*_HC_ and ^3^*J*_HC_ are shown for the indole, A/B junction rings, and side chain fragments.

### 2.2. Molecular Docking Study

It is well established that the various growth and developmental effects induced by BRs on plants start with the binding of these molecules to the extracellular region of a receptor kinase, BRI1 [34]. Therefore, this binding process has been extensively studied, and it has been shown that bioactivity can be correlated with the binding energies of BRs binding to the active site of *Arabidopsis thaliana*, which is calculated from molecular docking studies [35]. Thus, herein, preliminary molecular docking studies have been performed to determine the binding energies for the newly synthesized BR analogs. This is accomplished by placing new compounds within the active site of the BRI1–BAK1 crystallized complex (PDB: 4m7e) using AutoDock Vina. To validate the docking procedure, the natural ligand brassinolide is removed from the complex and then redocked into the crystallographic structure of BRI1–BAK1 (Figure S57, Supplementary Material). The results show that the lowest calculated energy, −12.9 kcal/mol, is associated with a pose that is quite similar to the crystallographic pose adopted by brassinolide.

Then, synthesized BR analogs **33**–**37** are located in the active site to determine the binding mode and key interactions within the active site, along with the binding free energy, for each of them (Figure S58, Supplementary Material).

For each compound, the pose of highest similarity to brassinolide and the lowest binding energy were selected, and their interactions were analyzed (Figures S59–S63, Supplementary Material). The calculated binding energies are listed in Table S1. The data indicate that the lowest binding energy (−13.7 kcal/mol), corresponding to compound **36**, is even lower than that obtained for brassinolide (−12.9 kcal/mol). This means that the complex formed by **36** within the active site of BRI1–BAK1 is more stable than that formed by brassinolide. This increase in stability can be explained in terms of different molecular interactions established by both compounds in the active site (Figure S64, Supplementary Material).

From a comparison of side chain structures, it becomes clear that hydrophobic interactions make an important contribution to the binding energy inside the active site of BRI1–BAK1. In addition, hydrogen bonds are formed by these two compounds, i.e., the NH group in the indole substituent of **36** interacts with Ser647 residue, and the carbonyl group with Tyr597, while the hydroxyl group of brassinolide interacts with the Ser647 residue and exhibits a π-alkyl interaction with the Tyr597 residue. Both amino acid residues could be important for improving the stability of BR derivatives through hydrogen bonds with Ser647 and Tyr597.

Interestingly, compound **33** is not able to form hydrogen bonds through its pyrrole group (Figure S65, Supplementary Material) despite being an indole isostere, like compound **36**, and this behavior might be attributed to the spatial orientation of this group. Pyrrole is smaller than the indole group, and therefore, it is located at a higher distance from the Ser647 residue, more specifically, 3.6 Å from the carbonyl group and 3.0 Å from the amine group in the peptidyl chain.

Thus, the results given by this preliminary molecular docking study suggest that **36** would be a very active molecule. To validate this prediction, we have carried out a molecular docking study of compound **23**, a compound that has previously been reported and that exhibits important activity in the RLIT bioassay [29]. These two compounds exhibit a notable structural similarity on the side chain even though they have different substituents attached at C-22; i.e., **36** has an indole fragment and **23** has a benzoate function. Because of this, the H atom in the NH group of **36** and the F atom in the ortho position of benzoate in **23** can be engaged in similar and relevant non-covalent interactions, halogen and hydrogen bonding. These interactions promote the ligand stabilization within the active site of the BRI1–BAK1 complex (Figure S66, Supplementary Material). In addition, the obtained interatomic distance of the NH group, in the indole fragment, from the peptide backbone of the Pro648 residue is 2.8 Å, while the fluorine atom of compound **23** is positioned at 2.9 Å from the same residue. As shown in Figure S62 (Supplementary Material), the carbonyl group of the benzoyl fragment is also oriented toward the same residue, with distances of 2.1 Å and 2.0 Å for BR derivatives **23** and **36**, respectively. Thus, these results reinforce the hypothesis of functional mimicry. Additionally, the calculated binding affinities for **23** and **36** are −12.4 kcal·mol^−1^ and −13.7 kcal·mol^−1^, respectively.

In summary, by comparing these results, it can be expected that **36** exhibits a potentially superior bioactivity to **23**, which has shown activity similar to that of brassinolide in the RLIT [29].

## 3. Materials and Methods

### 3.1. General Chemicals and Methods

All reagents were purchased from commercial suppliers and used without further purification. Melting points were measured on an SMP3 apparatus (Stuart-Scientific, now Merck KGaA, Darmstadt, Germany) and are uncorrected. ^1^H, ^13^C{^1^H}, ^13^C{^1^H} DEPT-135, gs 2D ^1^H-^13^C HSQC, and gs 2D ^1^H-^13^C HMBC NMR spectra were recorded in CDCl_3_ solutions on an Avance Neo 400 Digital NMR spectrometer (Bruker, Rheinstetten, Germany) operating at 400.1 MHz for ^1^H and 100.6 MHz for ^13^C and are referenced to the residual peaks of CHCl_3_ at δ = 7.26 ppm and δ = 77.00 ppm for ^1^H and ^13^C{^1^H}, respectively. Chemical shifts are reported in δ ppm, and coupling constants (*J*) are given in Hz; multiplicities are reported as follows: singlet (s), broad singlet (bs), doublet (d), broad doublet (bd), doublet of doublets (dd), doublet of triplets (dt), triplet (t), triplet of doublets (td), broad triplet (bt), quartet (q), doublet of quartets (dq), doublet of double doublets (ddd), triplet of triplets (tt), multiplet (m). IR spectra were recorded as KBr disks in a FT-IR 6700 spectrometer (Nicolet, Thermo Scientific, San Jose, CA, USA), and frequencies are reported in cm^−1^. High-resolution mass spectra (HRMS-ESI) were recorded in a Bruker Daltonik. The analysis for the reaction products was performed with the following relevant parameters: dry temperature, 180 °C; nebulizer, 0.4 Bar; dry gas, 4 L/min; and spray voltage, 4.5 kV at positive mode. The accurate mass measurements were performed at a resolving power: 140,000 FWHM at an *m*/*z* range of 50–1300. For analytical TLC, silica gel 60 in a 0.25 mm layer was used, and TLC spots were detected by heating after spraying with 25% H_2_SO_4_ in H_2_O. Chromatographic separations were carried out by a conventional column on silica gel 60 (230–400 mesh) using hexane–EtOAc mixtures of increasing polarity. All organic extracts were dried over anhydrous magnesium sulfate and evaporated under reduced pressure, below 40 °C.

### 3.2. Synthesis

#### 3.2.1. General Procedure of Steglich Esterification

Precursor **32** and Het-COOH were dissolved in benzene. Then *N*, *N*′-dicyclohexylcarbodiimide (DCC) and 4-*N*, *N*-dimethylaminopyridine (DMAP) were added. The mixture was stirred for 24 h at room temperature. The reaction was checked by TLC until all the starting material was consumed in the reaction. The mixture was subsequently poured into water, extracted with diethyl ether, washed with water, saturated by a KHCO_3_ solution, dried over sodium sulfate, and evaporated. The obtained ester was purified by column chromatography (C.C) and underwent elution with hexane/EtOAc (9:1).

*6-Oxo-23,24-dinor-5α-Cholan-2-en-22-yl-1H-pyrrole-2-carboxylate (**33a**)*.

Compound **32** (0.14 g, 0.421 mmol), benzene (20 mL), DMAP (182 mg), DCC (74.5 mg, 0.361 mmol), 1*H*-pyrrole-2-carboxylic acid (0.285 g, 2.591 mmol). Compound **33a** (0.13 g, 0.31 mmol, 73.1% yield) was obtained as a colorless solid (m.p. = 207.7–208.6 °C, Et_2_O/EtOAc). FT-IR_νmax_ (cm^−1^): 3019 (=C-H); 2965, 2931 and 2904 (-CH_3_); 2856 (CH_2_-); 1712 (C=O); 1698 (C=O); 1655 and 1553 (C=C); 1465 and 1413 (CH_2_-); 1384 (CH_3_-); 1252, 1235 and 1159 (C-O); 838, 772 and 670 (=C-H). ^1^H NMR (400.1 MHz, CDCl_3_): δ (ppm) = 9.34 (1H, bs, NH); 6.96 (1H, td, *J* = 2.5 and 1.5 Hz, H-3’); 6.91 (1H, ddd, *J* = 3.8, 2.5 and 1.5 Hz, H-5’); 6.26 (1H, dt, *J* = 3.8 and 2.5 Hz, H-4’); 5.70–5.66 (1H, m, H-3); 5.59–5.55 (1H, m, H-2); 4.25 (1H, dd, *J* = 10.7 and 3.5 Hz, H-22a); 4.00 (1H, dd, *J* = 10.7 and 7.2 Hz, H-22b); 2.38–2.33 (2H, m, H-5 and H-7); 2.30–2.22 (1H, m, H-4); 1.15 (1H, dd, *J* = 11.7, and 6.0 Hz, H-15); 1.09 (3H, d, *J* = 6.6 Hz, H-21); 0.713 (3H, s, H-19); 0.710 (3H, s, H-18). ^13^C{^1^H} NMR (100.6 MHz, CDCl_3_): δ (ppm) = 211.87 (C-6); 161.40 (CO_2_); 124.92 (C-3); 124.45 (C-2); 122.93 (C-2’); 122.79 (C-3’); 114.99 (C-5’); 110,31 (C-4’); 69.11 (C-22); 56.37 (C-14); 53.79 (C-5); 53.31 (C-9); 52.78 (C-17); 46.89 (C-7); 42.97 (C-13); 39.96 (C-10); 39.96 (C-1); 39.28 (C-12); 37,64 (C-20); 35.99 (C-8); 27.49 (C-16); 24.00 (C-15); 21.67 (C-4); 21.06 (C-11); 17.21 (C-21); 13.46 (C-19); 11.96 (C-18) (Figures S1–S5, Supplementary Material).

*6-Oxo-23,24-dinor-5α-Cholan-2-en-22-yl-1H-indole-2-carboxylate (**36a**)*.

Compound **32** (0.15 g, 0.454 mmol), benzene (25 mL), DMAP (195 mg), DCC (80.5 mg, 0.390 mmol), 1*H*-indole-2-carboxylic acid (0.308 g, 1.922 mmol). Compound **36a** (0.153 g, 0.32 mmol, 71.2% yield) was obtained as a colorless solid (m.p. = 223.9–224.5 °C, Et_2_O/EtOAc). FT-IR_νmax_ (cm^−1^): 3052 and 3023 (=C-H); 2970, 2966 and 2906 (-CH_3_); 2869, 2850 and 2828 (CH_2_-); 1720 (C=O); 1705 (C=O); 1619 and 1531 (C=C); 1430 (CH_2_-); 1375 (CH_3_-); 1273 and 1141 (C-O); 820, 773 and 666 (=C-H). ^1^H NMR (400.1 MHz, CDCl_3_): δ (ppm) = 8.98 (1H, bs, NH); 7.70 (1H, dd, *J* = 8.2 and 1.1 Hz, H-4’); 7.44 (1H, dd, *J* = 8.3 and 1.0 Hz, H-7’); 7.33 (1H, ddd, *J* = 8.2, 7.0 and 1.1 Hz, H-5’); 7.23 (1H, dd, *J* = 2.1 and 1.0 Hz, H-3’); 7.16 (1H, ddd, *J* = 8.3, 7.0 and 1.0 Hz, H-6’); 5.71–5.67 (1H, m, H-3); 5.60–5.55 (1H, m, H-2); 4.35 (1H, dd, *J* = 10.7 and 3.5 Hz, H-22a); 4.11 (1H, dd, *J* = 10.7 and 7.2 Hz, H-22b); 2.39–2.33 (2H, m, H-5 and H-7); 2.30–2.22 (1H, m, H-4); 1.76 (1H, ddd, *J* = 14.8, 10.7 and 4.1 Hz, H-20); 1.19–1.09 (1H, m, H-15); 1.14 (3H, d, *J* = 6.6 Hz, H-21); 0.738 (3H, s, H-19); 0.721 (3H, s, H-18). ^13^C{^1^H} NMR (100.6 MHz, CDCl_3_): δ (ppm) = 211.87 (C-6); 162.19 (CO_2_); 136.80 (C-8’); 127.46 (C-9’); 127.45 (C-2’); 125.36 (C-5’); 124.96 (C-3); 124.46 (C-2); 122.53 (C-4’); 120.83 (C-6’); 111.86 (C-7’); 108.51 (C-3’); 69.82 (C-22); 56.40 (C-14); 53.81 (C-5); 53.32 (C-9); 52.80 (C-17); 46.91 (C-7); 42.03 (C-13); 39.99 (C-10); 39.33 (C-1); 39.32 (C-12); 37.66 (C-20); 36.01 (C-8); 27.55 (C-16); 24.04 (C-15); 21.70 (C-4); 21.08 (C-11); 17.26 (C-21); 13.49 (C-19); 12.00 (C-18) (Figures S6–S10, Supplementary Material).

#### 3.2.2. Esterification Reaction with Heterocyclic Acid Chlorides of Compounds **34a**, **35a**, and **37a**

General procedure: Precursor **32** was dissolved in CH_2_Cl_2_ and pyridine. Later, DMAP and Het-COCl were added with slow stirring at room temperature. The end of the reaction was verified by TLC (2 h), solvent volume was reduced to about 10 mL, and then EtOAc (30 mL) was added. The organic layer was washed with 5% KHSO_4_ (2 × 15 mL) and water (2 × 15 mL), dried over Na_2_SO_4_, and filtered. The solvent was evaporated under reduced pressure. The crude was redissolved in CH_2_Cl_2_ (3 mL) and chromatographed on silica gel with hexane/EtOAc mixtures of increasing polarity (9:1).

*6-Oxo-23,24-dinor-5α-Cholan-2-en-22-yl-furan-2-carboxylate (**34a**)*.

Precursor **32** (0.10 g, 0.303 mmol), DCM (30 mL), Py (1.20 mL), DMAP (50 mg), furan-2-carbonyl chloride (0.15 mL, 1.403 mmol). Compound **34a** (0.90 g, 0.21 mmol, 70.0% yield) was obtained as a colorless solid (m.p. = 142.0–142.5 °C, Et_2_O/EtOAc). FT-IR_νmax_ (cm^−1^): 3137 and 3018 (=C-H); 2964 and 2945 (-CH_3_); 2869 and 2849 (CH_2_-); 1714 (C=O); 1573 (C=C); 1432 (CH_2_-); 1385 (CH_3_-); 1234 and 1183 (C-O); 884, 778 and 670 (=C-H). ^1^H NMR (400.1 MHz, CDCl_3_): δ (ppm) = 7.55 (1H, bt, *J* = 1.6 Hz, H-3’); 7.14 (1H, d, *J* = 3.4 Hz, H-5’); 6.48 (1H, dd, *J* = 3.4 and 1.6 Hz, H-4’); 5.67–5.63 (1H, m, H-3); 5.56–5.52 (1H, m, H-2); 4.25 (1H, dd, *J* = 10.6 and 3.5 Hz, H-22a); 4.01 (1H, dd, *J* = 10.6 and 7.5 Hz, H-22b); 2.35–2.30 (2H, m, H-5 and H-7); 2.26–2.18 (1H, m, H-4); 1.12 (1H, dt, *J* = 14.8, 10.6 and 6.6 Hz, H-15); 1.07 (3H, d, *J* = 6.6 Hz, H-21); 0.689 (3H, s, H-19); 0.682 (3H, s, H-18). ^13^C{^1^H} NMR (100.6 MHz, CDCl_3_): δ (ppm) = 211.66 (C-6); 158.81 (CO_2_); 146.13 (C-3’); 144.75 (C-2’); 124.85 (C-3); 124.37 (C-2); 117.54 (C-5’); 111.67 (C-4’); 69.61 C-22); 56.27 (C-14); 53.70 (C-5); 53.22 (C-9); 52.65 (C-17); 46.81 (C-7); 42.93 (C-13); 39.87 (C-10); 39.24 (C-1); 39.20 (C-12); 37.56 (C-20); 35.85 (C-8); 27.41 (C-16); 23.94 (C-15); 21.61 (C-4); 20.99 (C-11); 17.06 (C-21); 13.39 (C-19); 11.90 (C-18) (Figures S11–S15, Supplementary Material).

*6-Oxo-23,24-dinor-5α-Cholan-2-en-22-yl-thiophene-2-carboxylate (**35a**)*.

Precursor **32** (0.10 g, 0.303 mmol), DCM (30 mL), Py (1.20 mL), DMAP (50 mg), thiophene-2-carbonyl chloride (0.15 mL, 1.403 mmol). Compound **35a** (0.07 g, 0.159 mmol, 52.5% yield) was obtained as a colorless solid (m.p. = 152.9–153.2 °C, Et_2_O/EtOAc). FT-IR_νmax_ (cm^−1^): 3108 and 3019 (=C-H); 2966, 2936 and 2911 (-CH_3_); 2870 and 2823 (CH_2_-); 1712 (C=O); 1527 (C=C); 1417 (CH_2_-); 1357 (CH_3_-); 1288 and 1231 (C-O); 753, 738 and 672 (=C-H). ^1^H NMR (400.1 MHz, CDCl_3_): δ (ppm) = 7.79 (1H, dd, *J* = 3.8 and 1.2 Hz, H-3’); 7.54 (1H, dd, *J* = 5.0 and 1.2 Hz, H-5’); 7.09 (1H, dd, *J* = 5.0 and 3.8 Hz, H-4’); 5.69–5.65 (1H, m, H-3); 5.58–5.54 (1H, m, H-2); 4.27 (1H, dd, *J* = 10.7 and 3.7 Hz, H-22a); 4.02 (1H, dd, *J* = 10.7 and 7.2 Hz, H-22b); 2.37–2.32 (2H, m, H-5 and H-7); 2.29–2.20 (1H, m, H-4); 1.13 (1H, ddd, *J* = 11.4, 7.2 and 5.8 Hz, H-15); 1.10 (3H, d, *J* = 6.6 Hz, H-21); 0.712 (3H, s, H-19); 0.703 (3H, s, H-18). ^13^C{^1^H} NMR (100.6 MHz, CDCl_3_): δ (ppm) = 211.75 (C-6); 162.30 (CO_2_); 134.02 (C-2’); 133.14 (C-3’); 132.13 (C-5’); 127.68 (C-4’); 124.91 (C-3); 124.42 (C-2); 69.91 C-22); 56.35 (C-14); 53.76 (C-5); 53.28 (C-9); 52.75 (C-17); 46.87 (C-7); 42.95 (C-13); 39.93 (C-10); 39.29 (C-1); 39.27 (C-12); 37.62 (C-20); 35.91 (C-8); 27.47 (C-16); 23.99 (C-15); 21.66 (C-4); 21.04 (C-11); 17.18 (C-21); 13.45 (C-19); 11.95 (C-18) (Figures S16–S20, Supplementary Material).

*6-Oxo-23,24-dinor-5α-Cholan-2-en-22-yl-tetrahydro-2H-pyran-4-carbocarboxylate (**37a**)*.

Precursor **32** (0.15 g, 0.454 mmol), DCM (45 mL), Py (1.80 mL), DMAP (75 mg), tetrahydro-2*H*-pyran-4-carbonyl chloride (0.17 mL, 1.374 mmol). Compound **37a** (0.152 g, 0.343 mmol, 75.5% yield) was obtained as a colorless solid (m.p. = 115.1–115.7 °C, Et_2_O/EtOAc). FT-IR_νmax_ (cm^−1^): 3027 (=C-H); 2951 (-CH_3_); 2867 and 2841 (CH_2_-); 1735 (C=O); 1705 (C=O); 1655 (C=C); 1467 and 1444 (CH_2_-); 1386 (CH_3_-); 1279 and 1169 (C-O); 671 (=C-H). ^1^H NMR (400.1 MHz, CDCl_3_): δ (ppm) = 5.64–5.60 (1H, m, H-3); 5.53–5.48 (1H, m, H-2); 4.03 (1H, dd, *J* = 10.8 and 3.4 Hz, H-22a); 3.90 (2H, dt, *J* = 11.6 and 3.5 Hz, H-2’eq); 3.76 (1H, dd, *J* = 10.8 and 7.2 Hz, H-22b); 3.38 (2H, ddd, *J* = 11.6, 11.2 and 2.8 Hz, H-2’ax); 2.49 (1H, tt, *J* = 10.7 and 4.4 Hz, H-4’); 2.29 (2H, dd, *J* = 12.9 and 4.3 Hz, H-5 and H-7); 2.24–2.15 (1H, m, H-4); 1.38 (1H, dd, *J* = 12.7 and 3.6 Hz, H-11); 1.07 (1H, td, *J* = 11.9 and 6.1 Hz, H-15); 0.963 (3H, d, *J* = 6.7 Hz, H-21); 0.649 (3H, s, H-19); 0.642 (3H, s, H-18). ^13^C{^1^H} NMR (100.6 MHz, CDCl_3_): δ (ppm) = 211.52 (C-6); 174.37 (CO_2_); 124.77 (C-3); 124.31 (C-2); 69.18 (C-22); 66.92 (2 × C-2’); 56.23 (C-14); 53.62 (C-5); 53.13 (C-9); 52.55 (C-17); 46.72 (C-7); 42.76 (C-13); 40.08 (C-4’); 39.79 (C-10); 39.16 (C-1); 39.14 (C-12); 37.48 (C-20); 35.61 (C-8); 28.54 (2 × C-3’); 27.29 (C-16); 23.83 (C-15); 21.54 (C-4); 20.92 (C-11); 16.98 (C-21); 13.31 (C-19); 11.81 (C-18) (Figures S21–S26, Supplementary Material).

#### 3.2.3. Sharpless Dihydroxylation of Olefins **33**–**37**

General procedure for Sharpless dihydroxylation: Osmium tetroxide (OsO_4_) in *t*-butanol (1 g per 20 mL) was added to a solution of olefins **33a**–**37a**, hydroquinidine 4-chloro-benzoate (DHQD-CLB), methanesulfonamide (CH_3_SO_2_NH_2_), potassium carbonate (K_2_CO_3_), and potassium ferricyanide (K_3_[Fe(CN)_6_]) in a mixture of *t*-butanol and water (*t*-BuOH/H_2_O,1:1 *v*/*v*). The reaction mixture was stirred at room temperature for 36 h. A saturated solution of sodium sulfite (Na_2_SO_3_) was then added. After of additional 30 min of stirring, the reaction mixture was diluted with EtOAc (30 mL) and extracted with water (2 × 20 mL). The combined organic fractions were dried over anhydrous magnesium sulfate and evaporated under reduced pressure. Column chromatography on silica gel with hexane/EtOAc/MeOH mixtures of increasing polarity (6:4:0 → 4.8:4.8:0.4) led to the desired product.

*2α,3α-Dihydroxy-6-oxo-23,24-dinor-5α-cholan-22-yl-1H-pyrrole-2-carboxylate (**33**)*.

Olefin **33a** (0.10 g, 0.24 mmol), DHQD-CLB (0.03 g, 0.06 mmol), CH_3_SO_2_NH_2_ (0.04 g, 0.42 mmol), K_2_CO_3_ (0.19 g, 1.36 mmol), K_3_[Fe(CN)_6_] (0.68 g, 2.07 mmol), *t*-BuOH/H_2_O (15.0 mL), OsO_4_ (0.15 mL, 0.03 mmol). Compound **33** (0.09 g, 0.20 mmol, 83.3% yield) was obtained as a colorless solid (m.p. = 190.6–190.9 °C, Et_2_O/MeOH). FT-IR_νmax_ (cm^−1^): 3336 and 33,267 (O-H and N-H); 3020 and 2939 (-CH3); 2867 and 2846 (CH2-); 1697 (C=O); 1579 and 1556 (C=C); 1416 (CH2-); 1313 (CH3-); 1148 and 1080 (C-O); 882 and 779 (=C-H). ^1^H NMR (400.1 MHz, CDCl_3_): δ (ppm) = 9.26 (1H, bs, NH); 6.97 (1H, td, *J* = 2.6 and 1.4 Hz, H-3’); 6.91 (1H, ddd, *J* = 3.9, 2.6 and 1.4 Hz, H-5’); 6.27 (1H, dt, *J* = 3.9 and 2.6 Hz, H-4’); 4.87 (2H, bs, OH); 4.24 (1H, dd, *J* = 10.7 and 3.5 Hz, H-22a); 4.05 (1H, bs, H-3); 3.99 (1H, dd, *J* = 10.7 and 7.2 Hz, H-22b); 3.80–3.74 (1H, m, H-2); 2.68 (1H, dd, *J* = 12.3 and 3.4 Hz, H-5); 2.30 (1H, dd, *J* = 13.1 and 4.5 Hz, H-7α); 1.92 (1H, dt, *J* = 15.1 and 3.5 Hz, H-4β); 1.13 (1H, ddd, *J* = 17.2, 11.2 and 6.4 Hz, H-15); 1.09 (3H, d, *J* = 6.8 Hz, H-21); 0.756 (3H, s, H-19); 0.703 (3H, s, H-18). ^13^C{^1^H} NMR (100.6 MHz, CDCl_3_): δ (ppm) = 211.99 (C-6); 161.45 (CO_2_); 122.92 (C-2’); 122.84 (C-3’); 115.09 (C-5’); 110,39 (C-4’); 69.13 (C-22); 68.38 (C-3); 68.28 (C-2); 56.32 (C-14); 53.65 (C-9); 52.76 (C-17); 50.69 (C-5); 46.69 (C-7); 43.13 (C-13); 42.54 (C-10); 40.17 (C-1); 39.22 (C-12); 37,64 (C-20); 36.00 (C-8); 27.52 (C-16); 26.28 (C-4); 24.04 (C-15); 21.16 (C-11); 17.22 (C-21); 13.54 (C-19); 12.07 (C-18). HRMS-ESI (positive mode): *m*/*z* calculated for C_27_H_39_O_4_N: 457.2828 [M]^+^; found 458.2909 [M + H]^+^ (Figures S27–S32, Supplementary Material).

*2α,3α-Dihydroxy-6-oxo-23,24-dinor-5α-cholan-22-yl-furan-2-carboxylate (**34**)*.

Olefin **34a** (0.0811 g, 0.17 mmol), DHQD-CLB (0.0229 g, 0.0492 mmol), CH_3_SO_2_NH_2_ (0.0328 g, 0.345 mmol), K_2_CO_3_ (0.1455 g, 1.053 mmol), K_3_[Fe(CN)_6_] (0.366 g, 1.1104 mmol), *t*-BuOH/H_2_O (12.0 mL), OsO_4_ (0.10 mL, 0.0197 mmol). Compound **34** (0.05 g, 0.11 mmol, 57.6% yield) was obtained as a colorless solid (m.p. = 204.6–205.4 °C, Et_2_O/MeOH). FT-IR_νmax_ (cm^−1^): 3498 and 3414 (O-H); 3109 and 2958 (-CH_3_); 2897, 2872 and 2846 (CH_2_-); 1727 (C=O); 1704 (C=O); 1568 (C=C); 1475 (CH_2_-); 1397 (CH_3_-); 1238 and 1120 (C-O); 867, 782 and 765 (=C-H). ^1^H NMR (400.1 MHz, CDCl_3_): δ (ppm) = 7.56 (1H, dd, *J* = 1.8 and 0.8 Hz, H-3’); 7.14 (1H, dd, *J* = 3.5 and 0.8 Hz, H-5’); 6.49 (1H, dd, *J* = 3.5 and 1.8 Hz, H-4’); 4.25 (1H, dd, *J* = 10.7 and 3.5 Hz, H-22a); 4.02–3.98 (2H, m, H-3 and H-22b); 3.72 (1H, dt, *J* = 12.0 and 4.1 Hz, H-2); 2.70 (1H, bs, OH); 2.65 (1H, dd, *J* = 12.6 and 3.4 Hz, H-5); 2.58 (1H, bs, OH); 2.26 (1H, dd, *J* = 13.2 and 4.5 Hz, H-7α); 2.04–1.94 (2H, m, H-7β and H-12); 1.87 (1H, dt, *J* = 15.1 and 3.4 Hz, H-4β); 1.10 (1H, ddd, *J* = 12.1, 8.8 and 6.1 Hz, H-15); 1.06 (3H, d, *J* = 6.6 Hz, H-21); 0.724 (3H, s, H-19); 0.677 (3H, s, H-18). ^13^C{^1^H} NMR (100.6 MHz, CDCl_3_): δ (ppm) = 212.25 (C-6); 158.96 (CO_2_); 146.23 (C-3’); 144.68 (C-2’); 117.69 (C-5’); 111.73 (C-4’); 69.67 (C-22); 68.25 (C-3); 68.15 (C-2); 56.21 (C-14); 53.54 (C-9); 52.61 (C-17); 50.70 (C-5); 46.60 (C-7); 43.08 (C-13); 42.47 (C-10); 40.02 (C-1); 39.12 (C-12); 37.57 (C-20); 35.84 (C-8); 27.42 (C-16); 26.24 (C-4); 23.96 (C-15); 21.08 (C-11); 17.07 (C-21); 13.47 (C-19); 12.00 (C-18). HRMS-ESI (positive mode): *m*/*z* calculated for C_27_H_38_O_6_: 458.2668 [M]^+^; found 459.2747 [M + H]^+^ (Figures S33–S38, Supplementary Material).

*2α,3α-Dihydroxy-6-oxo-23,24-dinor-5α-cholan-22-yl-thiophene-2-carboxylate (**35**)*.

Olefin **35a** (0.06 g, 0.166 mmol), DHQD-CLB (0.0168 g, 0.0361 mmol), CH_3_SO_2_NH_2_ (0.0240 g, 0.252 mmol), K_2_CO_3_ (0.107 g, 0.771 mmol), K_3_[Fe(CN)_6_] (0.268 g, 0.813 mmol), *t*-BuOH/H_2_O (10.0 mL), OsO_4_ (0.10 mL, 0.0197 mmol). Compound **35** (0.052 g, 0.11 mmol, 66.3% yield) was obtained as a colorless solid (m.p. = 208.5–208.9 °C, Et_2_O/MeOH). FT-IR_νmax_ (cm^−1^): 3514 and 3465 (O-H); 2947 (-CH_3_); 2899 and 2867 (CH_2_-); 1708 (C=O); 1525 (C=C); 1416 (CH_2_-); 1382 (CH_3_-); 1260 and 1102 (C-O); 752 and 735 (=C-H). ^1^H NMR (400.1 MHz, CDCl_3_): δ (ppm) = 7.79 (1H, dd, *J* = 3.8 and 1.3 Hz, H-3’); 7.55 (1H, dd, *J* = 5.0 and 1.3 Hz, H-5’); 7.10 (1H, dd, *J* = 5.0 and 3.8 Hz, H-4’); 4.28 (1H, dd, *J* = 10.7 and 3.5 Hz, H-22a); 4.05–4.00 (2H, m, H-3 and H-22b); 3.76 (1H, dt, *J* = 11.0 and 3.2 Hz, H-2); 2.68 (1H, dd, *J* = 12.7 and 3.3 Hz, H-5); 2.30 (1H, dd, *J* = 13.1 and 4.6 Hz, H-7α); 1.17–1.12 (1H, m, H-15); 1.10 (3H, d, *J* = 6.6 Hz, H-21); 0.754 (3H, s, H-19); 0.706 (3H, s, H-18). ^13^C{^1^H} NMR (100.6 MHz, CDCl_3_): δ (ppm) = 211.96 (C-6); 162.40 (CO_2_); 134.02 (C-2’); 133.21 (C-3’); 132.21 (C-5’); 127.73 (C-4’); 69.94 (C-22); 68.36 (C-3); 68.25 (C-2); 56.31 (C-14); 53.64 (C-9); 52.73 (C-17); 50.69 (C-5); 46.68 (C-7); 43.12 (C-13); 42.52 (C-10); 40.16 (C-1); 39.21 (C-12); 37.63 (C-20); 35.93 (C-8); 27.50 (C-16); 26.27 (C-4); 24.03 (C-15); 21.15 (C-11); 17.20 (C-21); 13.54 (C-19); 12.07 (C-18). HRMS-ESI (positive mode): *m*/*z* calculated for C_27_H_38_O_5_S: 474.2440 [M]^+^; found 475.2507 [M + H]^+^ (Figures S39–S44, Supplementary Material).

*2α,3α-Dihydroxy-6-oxo-23,24-dinor-5α-cholan-22-yl-1H-indole-2-carboxylate (**36**)*.

Olefin **36a** (0.108 g, 0.229 mmol), DHQD-CLB (0.0275 g, 0.0591 mmol), CH_3_SO_2_NH_2_ (0.0394 g, 0.414 mmol), K_2_CO_3_ (0.174 g, 5.168 mmol), K_3_[Fe(CN)_6_] (0.438 g, 1.33 mmol), *t*-BuOH/H_2_O (17.0 mL), OsO_4_ (0.10 mL, 0.0197 mmol). Compound **36** (0.03 g, 0.06 mmol, 37.1% yield) was obtained as a colorless solid (m.p. = 202.1–202.5 °C, Et_2_O/MeOH). FT-IR_νmax_ (cm^−1^): 3416 (O-H); 2946 (-CH_3_); 2869 (CH_2_-); 1698 (C=O); 1530 (C=C); 1427 (CH_2_-); 1396 and 1380 (CH_3_-); 1245, 1198 and 1148 (C-O); 775 and 750 (=C-H). ^1^H NMR (400.1 MHz, CDCl_3_): δ (ppm) = 9.25 (1H, bs, NH); 7.70 (1H, d, *J* = 8.1 Hz, H-4’); 7.44 (1H, d, *J* = 8.3 Hz, H-7’); 7.32 (1H, dd, *J* = 8.1 and 7.5 Hz, H-5’); 7.23 (1H, d, *J* = 2.1 Hz, H-3’); 7.15 (1H, t, *J* = 7.5 Hz, H-6’); 5.04 (1H, bs, OH); 4.34 (1H, dd, *J* = 10.7 and 3.5 Hz, H-22a); 4.11–4.04 (2H, m, H-22a and H-3); 3.78–3.73 (1H, m, H-2); 2.67 (1H, dd, *J* = 12.9 and 3.3 Hz, H-5); 2.28 (1H, dd, *J* = 13.1 and 4.5 Hz, H-7α); 1.13 (3H, d, *J* = 6.6 Hz, H-21); 0.745 (3H, s, H-19); 0.706 (3H, s, H-18). ^13^C{^1^H} NMR (100.6 MHz, CDCl_3_): δ (ppm) = 212.16 (C-6); 162.38 (CO_2_); 136.92 (C-8’); 127.39 (C-9’); 127.36 (C-2’); 125.36 (C-5’); 122.50 (C-4’); 120.80 (C-6’); 111.95 (C-7’); 108.60 (C-3’); 69.81 (C-22); 68.37 (C-3); 68.27 (C-2); 56.25 (C-14); 53.56 (C-9); 52.70 (C-17); 50.67 (C-5); 46.61 (C-7); 43.11 (C-13); 42.50 (C-10); 40.10 (C-1); 39.19 (C-12); 37.59 (C-20); 35.97 (C-8); 27.52 (C-16); 26.27 (C-4); 23.99 (C-15); 21.12 (C-11); 17.22 (C-21); 13.51 (C-19); 12.04 (C-18). HRMS-ESI (positive mode): *m*/*z* calculated for C_31_H_41_NO_5_: 507.2985 [M]^+^; found 508.3069 [M + H]^+^ (Figures S45–S50, Supplementary Material).

*2α,3α-Dihydroxy-6-oxo-23,24-dinor-5α-cholan-22-yl-2H-pyran-4-carbocarboxylate (**37**)*.

Olefin **37a** (0.137 g, 0.308 mmol), DHQD-CLB (0.0370 g, 0.0796 mmol), CH_3_SO_2_NH_2_ (0.0530 g, 0.557 mmol), K_2_CO_3_ (0.235 g, 1.70 mmol), K_3_[Fe(CN)_6_] (0.891 g, 2.707 mmol), *t*-BuOH/H_2_O (20.0 mL), OsO_4_ (0.10 mL, 0.0197 mmol). Compound **37** (0.105 g, 0.22 mmol, 71.4% yield) was obtained as a colorless solid (m.p. = 153.0–154.0 °C, Et_2_O/MeOH). FT-IR_νmax_ (cm^−1^): 3520 and 3389 (O-H); 2946 (-CH_3_); 2886 and 2849 (CH_2_-); 1728 (C=O); 1709 (C=O); 1463 (CH_2_-); 1387 (CH_3_-); 1263 and 1118 (C-O); 932 (=C-H). ^1^H NMR (400.1 MHz, CDCl_3_): δ (ppm) = 4.03 (1H, dd, *J* = 10.7 and 3.4 Hz, H-22a); 4.03 (1H, bs, H-3); 3.96 (2H, dt, *J* = 11.6 and 3.6 Hz, H-2’eq); 3.80 (1H, dd, *J* = 10.7 and 7.1 Hz, H-22b); 3.76–3.71 (1H, m, H-2); 3.43 (2H, td, *J* = 11.4 and 2.9 Hz, H-2’ax); 2.67 (1H, dd, *J* = 12.7 and 3.4 Hz, H-5); 2.54 (1H, tt, *J* = 10.7 and 4.4 Hz, H-4’); 2.33 (1H, bs, OH); 2.28 (1H, dd, *J* = 13.1 and 4.6 Hz, H-7α); 2.10 (1H, bd, *J* = 5.9 Hz, OH); 1.90 (1H, dt, *J* = 15.2 and 3.5 Hz, H-4β); 1.11 (1H, td, *J* = 12.0 and 6.1 Hz, H-15); 1.01 (3H, d, *J* = 6.6 Hz, H-21); 0.743 (3H, s, H-19); 0.675 (3H, s, H-18). ^13^C{^1^H} NMR (100.6 MHz, CDCl_3_): δ (ppm) = 212.00 (C-6); 174.62 (CO_2_); 69.36 (C-22); 68.34 (C-3); 68.20 (C-2); 67.10 (2 × C-2’); 56.30 (C-14); 53.61 (C-9); 52.64 (C-17); 50.69 (C-5); 46.66 (C-7); 43.05 (C-13); 42.51 (C-10); 40.24 (C-4’); 40.14 (C-1); 39.19 (C-12); 37.61 (C-20); 35.74 (C-8); 28.67 (2 × C-3’); 27.43 (C-16); 26.27 (C-4); 23.97 (C-15); 21.13 (C-11); 17.10 (C-21); 13.52 (C-19); 12.04 (C-18) HRMS-ESI (positive mode): *m*/*z* calculated for C_28_H_44_O_6_: 476.3138 [M]^+^; found 477.3207 [M + H]^+^ (Figures S51–S56, Supplementary Material).

### 3.3. Molecular Docking

The tridimensional structure of BRI1-BAK1 was obtained from the Protein Data Bank (PDB ID: 4M7E) [35]. The crystallized ligand (BLD808) was removed by using ArgusLab software 4.0.1 (Mark Thompson and Planaria Software LLC, Seattle, WA, USA, 1997–2004). Then, this structure was loaded into AutoDock Tools 1.5.6 (MGLTools,). Here, polar H atoms were added, Gasteiger–Hückel charges were assigned, and the results were saved in .pdbqt format. Ligands were designed in ChemDraw 3D 15.1.0.144 (PerkinElmer Informatics, Inc., Waltham, WA, USA, 1998–2016), making sure that all chiral centers were correctly assigned. These structures were exported in .mol2 format and processed in AutoDock Tools 1.5.6.

The coupling site was defined as the location of the native ligand, using the box center coordinates x = −10.54, y = −30.20, z = −27.77 and dimensions of 34 Å × 16 Å × 40 Å along the x, y, and z axes, respectively. These configurations were then used in the molecular coupling simulations performed with AutoDock Vina 1.1.2 [36]. The 20 best conformations for each tested compound were registered. The obtained results were then visually analyzed by using PyMOL™ 1.7.5.4 Edu (Schrödinger, LLC, New York, NY, USA). Molecular docking of compounds **33**–**37** in the active site of BRI1–BAK1, 3D visualization, and 2D representations of the most important interactions are shown in Figures S59–S63 (Supplementary Material).

## 4. Conclusions

The synthesis and characterization of five new BR analogs with heterocyclic acyl groups attached to C-22 are described. The acylation reaction at C-22 was carried out by two different methods, namely acylation with heterocyclic acid chlorides and Steglich esterification reaction. In both cases, the acyl derivatives were obtained with good yields (52.5–75.5%).

Molecular docking results indicate that the complex formed by **36** within the active site of BRI1–BAK1 is more stable than that formed by brassinolide (**1**). The calculated binding energies are −13.7 kcal/mol and −12.9 kcal/mol for **36** and brassinolide (**1**), respectively. This increase in complex stability can be explained in terms of a more effective hydrogen bonding of the N atoms of the N-H group in the indole with Ser647 or Pro648 amino acids. This effect is a consequence of a special orientation in the active site of BRI1–BAK1 forced by the heterocyclic ring. These conclusions are supported by a comparison of docking results obtained for **36** and **23**. Both molecules have similar poses and interactions in the active site of BRI1–BAK1, and the binding energy of **36** is lower than that obtained for **23**. Based on this data, and considering that **23** has shown bioactivity comparable to that shown by brassinolide, it is concluded that **36** should be as active as **1**.

Confirmation of this predictive behavior would ensure that theoretical studies of biological activities in plant growth stimulation could be relevant to designing new brassinosteroid analogs. Measurements of biological activities in different bioassays are currently in progress.

## Figures and Tables

**Figure 5:**
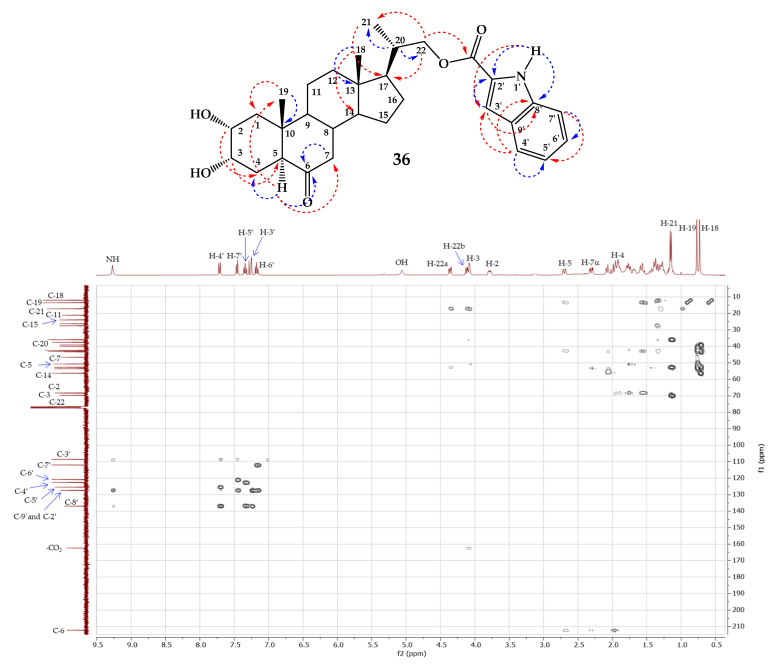
Top: structure of compound **36**; bottom: 2D ^1^H-^13^C HMBC spectrum. Main ^2^*J*_HC_ (blue) and ^3^*J*_HC_ (red) correlations observed for indole moiety, A/B rings, and side chain fragments.

## Data Availability

The original contributions presented in this study are included in the article/Supplementary Material. Further inquiries can be directed to the corresponding author.

## Supplementary Materials

The following supporting information can be downloaded at https://www.mdpi.com/article/10.3390/molecules30194011/s1: Figures S1–S26: ^1^H, ^13^C{^1^H}, ^13^C{^1^H} DEPT-135, 2D ^1^H-^13^C HSQC, and 2D ^1^H-^13^C HMBC NMR spectra for compounds **33a**–**37a**. Figures S27–S56: ^1^H, ^13^C{^1^H}, ^13^C{^1^H} DEPT-135, 2D ^1^H-^13^C HSQC, and 2D^1^H-^13^C HMBC NMR and HRMS spectra for compounds **33**–**37**. Figures S57–S65: Results of molecular docking of compounds **33**–**37**.

## References

[1] Peres A.L.G.L., Soares J.S., Tavares R.G., Righetto G., Zullo M.A.T., Mandava N.B., Menossi M. (2019). Brassinosteroids, the Sixth Class of Phytohormones: A Molecular View from the Discovery to Hormonal Interactions in Plant Development and Stress Adaptation. Int. J. Mol. Sci..

[2] Bajguz A., Chmur M., Gruszka D. (2020). Comprehensive Overview of the Brassinosteroid Biosynthesis Pathways: Substrates, Products, Inhibitors, and Connections. Front. Plant Sci..

[3] Bajguz A. (2007). Metabolism of brassinosteroids in plants. Plant Physiol. Biochem..

[4] Garrido-Auñón F., Puente-Moreno J., García-Pastor M.E., Serrano M., Valero D. (2024). Brassinosteroids: An Innovative Compound Family That Could Affect the Growth, Ripening, Quality, and Postharvest Storage of Fleshy Fruits. Plants.

[5] Bajguz A., Hayat S. (2009). Effects of brassinosteroids on the plant responses to environmental stresses. Plant Physiol. Biochem..

[6] Wei Z., Li J. (2016). Brassinosteroids Regulate Root Growth, Development, and Symbiosis. Mol. Plant.

[7] Wendeborn S., Lachia M., Jung P.M.J., Leipner J., Brocklehurst D., De Mesmaeker A., Gaus K., Mondière R. (2017). Biological Activity of Brassinosteroids—Direct Comparison of Known and New Analogs in planta. Helv. Chim. Acta.

[8] Ahanger M.A., Ashraf M., Bajguz A., Ahmad P. (2018). Brassinosteroids Regulate Growth in Plants Under Stressful Environments and Crosstalk with Other Potential Phytohormones. J. Plant Growth Regul..

[9] Nolan T.M., Vukašinović N., Liu D., Russinova E., Yin Y. (2020). Brassinosteroids: Multidimensional Regulators of Plant Growth, Development, and Stress Responses. Plant Cell.

[10] Bajguz A., Piotrowska-Niczyporuk A., Tran L.-S.P., Pal S. (2014). Brassinosteroids Implicated in Growth Stress Responses. Phytohormones: A Window to Metabolism Signaling Biotechnological Applications.

[11] Siddiqui H., Hayat S., Bajguz A. (2018). Regulation of photosynthesis by brassinosteroids in plants. Acta Physiol. Plant.

[12] Manghwar H., Hussain A., Ali Q., Liu F. (2022). Brassinosteroids (BRs) Role in Plant Development and Coping with Different Stresses. Int. J. Mol. Sci..

[13] Fedina E., Yarin A., Mukhitova F., Blufard A., Chechetkin I. (2017). Brassinosteroid-induced changes of lipid composition in leaves of *Pisum sativum* L. during senescence. Steroids.

[14] Bajguz A., Tretyn A. (2003). The chemical characteristic and distribution of brassinosteroids in plants. Phytochemistry.

[15] Yokota T., Ohnishi T., Shibata K., Asahina M., Nomura T., Fujita T., Ishizaki K., Kohchi T. (2017). Occurrence of brassinosteroids in non-flowering land plants, liverwort, moss, lycophyte and fern. Phytochemistry.

[16] Bajguz A., Hayat S., Yusuf M., Bhardwaj R., Bajguz A. (2019). Brassinosteroids in Microalgae: Application for Growth Improvement and Protection Against Abiotic Stresses. Brassinosteroids: Plant Growth and Development.

[17] Zullo M.A.T., Bajguz A., Hayat S., Yusuf M., Bhardwaj R., Bajguz A. (2019). The Brassinosteroids Family—Structural Diversity of Natural Compounds and Their Precursors. Brassinosteroids: Plant Growth and Development.

[18] Hayat S., Ahmad A. (2003). Brassinosteroids: Bioactivity and Crop Productivity.

[19] Temmem O., Uguen D., De Cian A., Gruber N. (2002). Toward a total synthesis of brassinosteroids; structure assessment of the Ireland–Claisen products of geranyl and neryl esters. Tetrahedron Lett..

[20] Temmem O., Zoller T., Uguen D. (2002). Toward a total synthesis of brassinosteroids; stereoselective generation of the hydrindane ring system. Tetrahedron Lett..

[21] Back T.G., Atta-ur R. (1995). Stereoselective synthesis of brassinosteroids. Studies in Natural Products Chemistry.

[22] Kovganko N.V., Ananich S.K. (1997). Advances in the chemical synthesis of brassinosteroids. Chem. Nat. Compd..

[23] Khripach V.A., Zhabinskii V.N., de Groot A.E., Khripach V.A., Zhabinskii V.N., de Groot A.E. (1999). Chapter VI—Basic synthetic methods and formal syntheses. Brassinosteroids. A New Class of Plant Hormones.

[24] Khripach V., Zhabinskii V., Ermolovich Y. (2015). Synthetic Aspects of Brassinosteroids. Stud. Nat. Prod. Chem..

[25] Back T.G., Janzen L., Nakajima S.K., Pharis R.P. (2000). Effect of Chain Length and Ring Size of Alkyl and Cycloalkyl Side-Chain Substituents upon the Biological Activity of Brassinosteroids. Preparation of Novel Analogues with Activity Exceeding that of Brassinolide. J. Org. Chem..

[26] Huang L.F., Zhou W.S. (1994). Studies on Steroidal Plant-Growth Regulators. Part 33. Novel Method for Construction of the Side-Chain of 23-Arylbrassinosteroids Via Heck Arylation and Asymmetric Dihydroxylation As Key Steps. J. Chem. Soc. Perkin Trans. 1.

[27] Kvasnica M., Oklestkova J., Bazgier V., Rárová L., Korinkova P., Mikulík J., Budesinsky M., Béres T., Berka K., Lu Q. (2016). Design, synthesis and biological activities of new brassinosteroid analogues with a phenyl group in the side chain. Org. Biomol. Chem..

[28] Korinkova P., Bazgier V., Oklestkova J., Rarova L., Strnad M., Kvasnica M. (2017). Synthesis of novel aryl brassinosteroids through alkene cross-metathesis and preliminary biological study. Steroids.

[29] Aitken V., Diaz K., Soto M., Olea A.F., Cuellar M.A., Nuñez M., Espinoza-Catalán L. (2024). New Brassinosteroid Analogs with 23,24-Dinorcholan Side Chain, and Benzoate Function at C-22: Synthesis, Assessment of Bioactivity on Plant Growth, and Molecular Docking Study. Int. J. Mol. Sci..

[30] Nuñez M., Wang Y., Russinova E., Estévez-Braun A., Amesty A., Olea A.F., Mellado M., Díaz K., Espinoza Catalán L. (2024). Synthesis, Biological Activity, and Molecular-Docking Studies of New Brassinosteroid Analogs. Int. J. Mol. Sci..

[31] Diachkov M.V., Ferrer K., Oklestkova J., Rarova L., Bazgier V., Kvasnica M. (2021). Synthesis and Biological Activity of Brassinosteroid Analogues with a Nitrogen-Containing Side Chain. Int. J. Mol. Sci..

[32] Rárová L., Sedlák D., Oklestkova J., Steigerová J., Liebl J., Zahler S., Bartůněk P., Kolář Z., Kohout L., Kvasnica M. (2018). The novel brassinosteroid analog BR4848 inhibits angiogenesis in human endothelial cells and induces apoptosis in human cancer cells in vitro. J. Steroid Biochem. Mol. Biol..

[33] Munawar S., Zahoor A.F., Hussain S.M., Ahmad S., Mansha A., Parveen B., Ali K.G., Irfan A. (2024). Steglich esterification: A versatile synthetic approach toward the synthesis of natural products, their analogues/derivatives. Heliyon.

[34] Vert G., Nemhauser J.L., Geldner N., Hong F., Chory J. (2005). Molecular mechanisms of steroid hormone signaling in plants. Annu. Rev. Cell Dev. Biol..

[35] Diaz K., Espinoza L., Carvajal R., Conde-Gonzalez M., Niebla V., Olea A.F., Coll Y. (2020). Biological Activities and Molecular Docking of Brassinosteroids 24-Norcholane Type Analogs. Int. J. Mol. Sci..

[36] Trott O., Olson A.J. (2010). AutoDock Vina: Improving the speed and accuracy of docking with a new scoring function, efficient optimization, and multithreading. J. Comput. Chem..

