# Supplementary material for: Synthesis of New Brassinosteroid Analogs with Androstane Skeleton and Heterocyclic Acyl Side Chains: Preliminary Molecular Docking Studies
Source: Molecules. 2025 Oct 7;30(19):4011. doi: 10.3390/molecules30194011 (PMC12526290; doi:10.3390/molecules30194011)
Supplement: Supplementary file 1 [file molecules-30-04011-s001.zip › molecules-3859383-supplementary.pdf]

Supplementary Material

Synthesis of New Brassinosteroid Analogs with Androstane Skeleton and Heterocyclic Acyl Side Chains. Preliminary Molecular Docking Studies.

Omara Araya [1], María Núñez [1]*, Marco Mellado [2], Andrés F. Olea [3] and Luis Espinoza-Catalán [1]*

[1] Departamento de Química, Universidad Técnica Federico Santa María, Avenida España 1680, Valparaíso 2340000, Chile; omara.araya@usm.cl (O.A.); maria.nunezg@usm.cl (M.N.); luis.espinozac@usm.cl. (L.E.-C.)
[2] Centro de Investigación en Ingeniería de Materiales, Universidad Central de Chile, Santiago 8330507, Chile; marco.mellado@ucentral.cl (M.M.)
[3] Instituto de Ciencias Aplicadas, Facultad de Ingeniería, Universidad Autónoma de Chile, Av. del Valle Sur 534, Santiago, Chile and CP 8580640; andres.olea@uautonoma.cl (A.F.O)
* Correspondence: maria.nunezg@usm.cl (M.N.); luis.espinozac@usm.cl (L.E.-C)

Index **pag.**

Figure S1. ^{1}H NMR spectrum of 6-Oxo-23,24-dinor-5α-Cholan-2-en-22-yl-1*H*-pyrrole-2-carboxylate (**33a**)........ **6**

Figure S2. ^{13}C{^{1}H} NMR spectrum of 6-Oxo-23,24-dinor-5α-Cholan-2-en-22-yl-1*H*-pyrrole-2-carboxylate (**33a**). **6**

Figure S3. ^{13}C{^{1}H} DEPT-135 NMR spectrum of 6-Oxo-23,24-dinor-5α-Cholan-2-en-22-yl-1H-pyrrole-2-carboxylate (**33a**). **7**

Figure S4. 2D ^{1}H-^{13}C HSQC spectrum of 6-Oxo-23,24-dinor-5α-Cholan-2-en-22-yl-1H-pyrrole-2-carboxylate (**33a**). **7**

Figure S5. 2D ^{1}H-^{13}C HMBC spectrum of 6-Oxo-23,24-dinor-5α-Cholan-2-en-22-yl-1H-pyrrole-2-carboxylate (**33a**). **8**

Figure S6. ^{1}H NMR spectrum of 6-Oxo-23,24-dinor-5α-Cholan-2-en-22-yl-1H-indole-2-carboxylate (**36a**). **8**

Figure S7. ^{13}C{^{1}H} NMR spectrum of 6-Oxo-23,24-dinor-5α-Cholan-2-en-22-yl-1H-indole-2-carboxylate (**36a**). **9**

Figure S8. ^{13}C{^{1}H} DEPT-135 NMR spectrum of 6-Oxo-23,24-dinor-5α-Cholan-2-en-22-yl-1H-indole-2-carboxylate (**36a**). **9**

Figure S9. 2D ^{1}H-^{13}C HSQC spectrum of 6-Oxo-23,24-dinor-5α-Cholan-2-en-22-yl-1H-indole-2-carboxylate (**36a**). **10**

Figure S10. 2D ^{1}H-^{13}C HMBC spectrum of 6-Oxo-23,24-dinor-5α-Cholan-2-en-22-yl-1H-indole-2-carboxylate (**36a**). **10**

 https://doi.org/10.3390/xxxxx

Figure S11. ^{1}H NMR spectrum of 6-Oxo-23,24-dinor-5α-Cholan-2-en-22-yl-furan-2-carboxylate (**34a**). …………………………………………………………………… **11**

Figure S12. $^{13}C\{^{1}H\}$ NMR spectrum of 6-Oxo-23,24-dinor-5α-Cholan-2-en-22-yl-furan-2-carboxylate (**34a**). ……………………………………………………………….. **11**

Figure S13. $^{13}C\{^{1}H\}$ DEPT-135 NMR spectrum of 6-Oxo-23,24-dinor-5α-Cholan-2-en-22-yl-furan-2-carboxylate (**34a**). ………………………………………………….. **12**

Figure S14. 2D ^{1}H-^{13}C HSQC spectrum of 6-Oxo-23,24-dinor-5α-Cholan-2-en-22-yl-furan-2-carboxylate (**34a**). ………………………………………………………… **12**

Figure S15. 2D ^{1}H-^{13}C HMBC spectrum of 6-Oxo-23,24-dinor-5α-Cholan-2-en-22-yl-furan-2-carboxylate (**34a**). ………………………………………………………… **13**

Figure S16. ^{1}H NMR spectrum of 6-Oxo-23,24-dinor-5α-Cholan-2-en-22-yl-thiophene-2-carboxylate (**35a**). ………………………………………………………………… **13**

Figure S17. $^{13}C\{^{1}H\}$ NMR spectrum of 6-Oxo-23,24-dinor-5α-Cholan-2-en-22-yl-thiophene-2-carboxylate (**35a**). ……………………………………………………….. **14**

Figure S18. $^{13}C\{^{1}H\}$ DEPT-135 NMR spectrum of 6-Oxo-23,24-dinor-5α-Cholan-2-en-22-yl-thiophene-2-carboxylate (**35a**). …………………………………………….. **14**

Figure S19. 2D ^{1}H-^{13}C HSQC spectrum of 6-Oxo-23,24-dinor-5α-Cholan-2-en-22-yl-thiophene-2-carboxylate (**35a**). ……………………………………………… **15**

Figure S20. 2D ^{1}H-^{13}C HMBC spectrum of 6-Oxo-23,24-dinor-5α-Cholan-2-en-22-yl-thiophene-2-carboxylate (**35a**). ……………………………………………… **15**

Figure S21. ^{1}H NMR spectrum of 6-Oxo-23,24-dinor-5α-Cholan-2-en-22-yl-tetrahydro-2H-pyran-4-carbocarboxylate (**37a**). ………………………………………… **16**

Figure S22. $^{13}C\{^{1}H\}$ NMR spectrum of 6-Oxo-23,24-dinor-5α-Cholan-2-en-22-yl-tetrahydro-2H-pyran-4-carbocarboxylate (**37a**). ………………………………... **16**

Figure S23. $^{13}C\{^{1}H\}$ DEPT-135 NMR spectrum of 6-Oxo-23,24-dinor-5α-Cholan-2-en-22-yl-tetrahydro-2H-pyran-4-carbocarboxylate (**37a**). ………………………….. **17**

Figure S24. 2D HSQC ^{1}H-^{13}C spectrum of 6-Oxo-5α-androst-2-en-23,24-dinor-22-tetrahydro-2*H*-pyran-4-carbocarboxylate-22-yl (**37a**). ……………………………. **17**

Figure S25. 2D ^{1}H-^{13}C HMBC spectrum of 6-Oxo-23,24-dinor-5α-Cholan-2-en-22-yl-tetrahydro-2H-pyran-4-carbocarboxylate (**37a**). ………………………………... **18**

Figure S26. 1D selective NOESY NMR spectrum of 6-Oxo-23,24-dinor-5α-Cholan-2-en-22-yl-tetrahydro-2H-pyran-4-carbocarboxylate (**37a**). ……………………….. **18**

Figure S27. ^{1}H NMR spectrum of 2α,3α-Dihydroxy-6-oxo-23,24-dinor-5α-cholan-22-yl-1H-pyrrole-2-carboxylate (**33**). ……………………………………………… **19**

Figure S28. $^{13}C\{^{1}H\}$ NMR spectrum of 2α,3α-Dihydroxy-6-oxo-23,24-dinor-5α-cholan-22-yl-1H-pyrrole-2-carboxylate (**33**). ………………………………………… **19**

Figure S29. $^{13}C\{^{1}H\}$ DEPT-135 NMR spectrum of 2α,3α-Dihydroxy-6-oxo-23,24-dinor-5α-cholan-22-yl-1H-pyrrole-2-carboxylate (**33**). …………………………….. 20

Figure S30. 2D ^{1}H-^{13}C HSQC spectrum of 2α,3α-Dihydroxy-6-oxo-23,24-dinor-5α-cholan-22-yl-1H-pyrrole-2-carboxylate (**33**). ………………………………….. 20

Figure S31. 2D ^{1}H-^{13}C HMBC spectrum of 2α,3α-Dihydroxy-6-oxo-23,24-dinor-5α-cholan-22-yl-1H-pyrrole-2-carboxylate (**33**). …………………………………. 21

Figure S32. HRMS spectrum of 2α,3α-Dihydroxy-6-oxo-23,24-dinor-5α-cholan-22-yl-1H-pyrrole-2-carboxylate (**33**). ………………………………………………. 21

Figure S33. ^{1}H NMR spectrum of 2α,3α-Dihydroxy-6-oxo-23,24-dinor-5α-cholan-22-yl-furan-2-carboxylate (**34**). ……………………………………………………. 22

Figure S34. $^{13}C\{^{1}H\}$ NMR spectrum of 2α,3α-Dihydroxy-6-oxo-23,24-dinor-5α-cholan-22-yl-furan-2-carboxylate (**34**). ………………………………………………… 22

Figure S35. $^{13}C\{^{1}H\}$ DEPT-135 NMR spectrum of 2α,3α-Dihydroxy-6-oxo-23,24-dinor-5α-cholan-22-yl-furan-2-carboxylate (**34**). …………………………………….. 23

Figure S36. 2D ^{1}H-^{13}C HSQC spectrum of 2α,3α-Dihydroxy-6-oxo-23,24-dinor-5α-cholan-22-yl-furan-2-carboxylate (**34**). ……………………………………….. 23

Figure S37. 2D ^{1}H-^{13}C HMBC spectrum of 2α,3α-Dihydroxy-6-oxo-23,24-dinor-5α-cholan-22-yl-furan-2-carboxylate (**34**). ………………………………………… 24

Figure S38. HRMS spectrum of 2α,3α-Dihydroxy-6-oxo-23,24-dinor-5α-cholan-22-yl-furan-2-carboxylate (**34**). ……………………………………………………….. 24

Figure S39. ^{1}H NMR spectrum of 2α,3α-Dihydroxy-6-oxo-23,24-dinor-5α-cholan-22-yl-thiophene-2-carboxylate (**35**). ………………………………………………….. 25

Figure S40. $^{13}C\{^{1}H\}$ NMR spectrum of 2α,3α-Dihydroxy-6-oxo-23,24-dinor-5α-cholan-22-yl-thiophene-2-carboxylate (**35**). …………………………………………… 25

Figure S41. $^{13}C\{^{1}H\}$ DEPT-135 NMR spectrum of 2α,3α-Dihydroxy-6-oxo-23,24-dinor-5α-cholan-22-yl-thiophene-2-carboxylate (**35**). ……………………………….. 26

Figure S42. 2D ^{1}H-^{13}C HSQC spectrum of 2α,3α-Dihydroxy-6-oxo-23,24-dinor-5α-cholan-22-yl-thiophene-2-carboxylate (**35**). . …………………………………. 26

Figure S43. 2D ^{1}H-^{13}C HMBC spectrum of 2α,3α-Dihydroxy-6-oxo-23,24-dinor-5α-cholan-22-yl-thiophene-2-carboxylate (**35**). …………………………………… 27

Figure S44. HRMS spectrum of 2α,3α-Dihydroxy-6-oxo-23,24-dinor-5α-cholan-22-yl-thiophene-2-carboxylate (**35**). …………………………………………………... 27

Figure S45. ^{1}H NMR spectrum of 2α,3α-Dihydroxy-6-oxo-23,24-dinor-5α-cholan-22-yl-1H-indole-2-carboxylate (**36**). …………………………………………………... 28

Figure S46. $^{13}C\{^{1}H\}$ NMR spectrum of 2α,3α-Dihydroxy-6-oxo-23,24-dinor-5α-cholan-22-yl-1H-indole-2-carboxylate (**36**). …………………………………………… 28

Figure S47. $^{13}C\{^{1}H\}$ DEPT-135 NMR spectrum of 2α,3α-Dihydroxy-6-oxo-23,24-dinor-5α-cholan-22-yl-1H-indole-2-carboxylate (**36**). ……………………………….. 29

Figure S48. 2D ^{1}H-^{13}C HSQC spectrum of 2α,3α-Dihydroxy-6-oxo-23,24-dinor-5α-cholan-22-yl-1H-indole-2-carboxylate (**36**). …………………………………… 29

Figure S49. 2D ^{1}H-^{13}C HMBC spectrum of 2α,3α-Dihydroxy-6-oxo-23,24-dinor-5α-cholan-22-yl-1H-indole-2-carboxylate (**36**). …………………………………… 30

Figure S50. HRMS spectrum of 2α,3α-Dihydroxy-6-oxo-23,24-dinor-5α-cholan-22-yl-1H-indole-2-carboxylate (**36**). ……………………………………………….. 30

Figure S51. ^{1}H NMR spectrum of 2α,3α-Dihydroxy-6-oxo-23,24-dinor-5α-cholan-22-yl-2H-pyran-4-carbocarboxylate (**37**). ……………………………………………. 31

Figure S52. $^{13}C\{^{1}H\}$ NMR spectrum of 2α,3α-Dihydroxy-6-oxo-23,24-dinor-5α-cholan-22-yl-2H-pyran-4-carbocarboxylate (**37**). …………………………………….. 31

Figure S53. $^{13}C\{^{1}H\}$ DEPT-135 NMR spectrum of 2α,3α-Dihydroxy-6-oxo-23,24-dinor-5α-cholan-22-yl-2H-pyran-4-carbocarboxylate (**37**). …………………………. 32

Figure S54. 2D ^{1}H-^{13}C HSQC spectrum of 2α,3α-Dihydroxy-6-oxo-23,24-dinor-5α-cholan-22-yl-2H-pyran-4-carbocarboxylate (**37**). …………………………….. 32

Figure S55. 2D ^{1}H-^{13}C HMBC spectrum of 2α,3α-Dihydroxy-6-oxo-23,24-dinor-5α-cholan-22-yl-2H-pyran-4-carbocarboxylate (**37**). …………………………….. 33

Figure S56. HRMS spectrum of 2α,3α-Dihydroxy-6-oxo-23,24-dinor-5α-cholan-22-yl-2H-pyran-4-carbocarboxylate (**37**). ……………………………………………. 33

Figure S57. Redocking of brassinolide into the active site of the BRI1–BAK1. BRI1–BAK1 in cartoon mode and ligands in stick mode…………………………….. 34

Figure S58. Results of molecular docking for new BRs analogs. BRI1–BAK1 in cartoon mode and ligands in stick mode………………………………………………… 34

Figure S59 Results of molecular docking of compound **33** in the active site of BRI1–BAK1. A. 3D visualization and B. 2D representations of the most important interactions. ………………………………………………………………………… 34

Figure S60. Results of molecular docking of compound **34** in the active site of BRI1–BAK1. A. 3D visualization and B. 2D representations of the most important interactions. ………………………………………………………………………… 35

Figure S61. Results of molecular docking of compound **35** in the active site of BRI1–BAK1. A. 3D visualization and B. 2D representations of the most important interactions. ………………………………………………………………………… 35

Figure S62. Results of molecular docking of compound **36** in the active site of BRI1–BAK1. A. 3D visualization and B. 2D representations of the most important interactions. ………………………………………………………………………… 35

Figure S63. Results of molecular docking of compound **37** in the active site of BRI1–BAK1. A. 3D visualization and B. 2D representations of the most important interactions. **36**

Figure S64 Results of molecular docking for compound **36** and brassinolide within the active site of BRI1–BAK1. A. 3D visualization of compound **36**; B. 2D representation of interactions of **36** with residues in BRI1–BAK1; C. 3D visualization of brassinolide docked to the active site; D. 2D representation of interactions of brassinolide with residues in BRI1–BAK1........ **36**

Figure S65 Molecular docking results for **33** and **36** in the active site of BRI1–BAK1, showing the aminoacids and distance for potential hydrogen bonding. Color code: Blue: C atoms of **33**; White: C atoms compound **33**; Green: C atomos of BRI1–BAK1........ **37**

Table S1 Binding energies and interactions obtained for BRs analogs (**33-37**) and brassinolide........ **38**

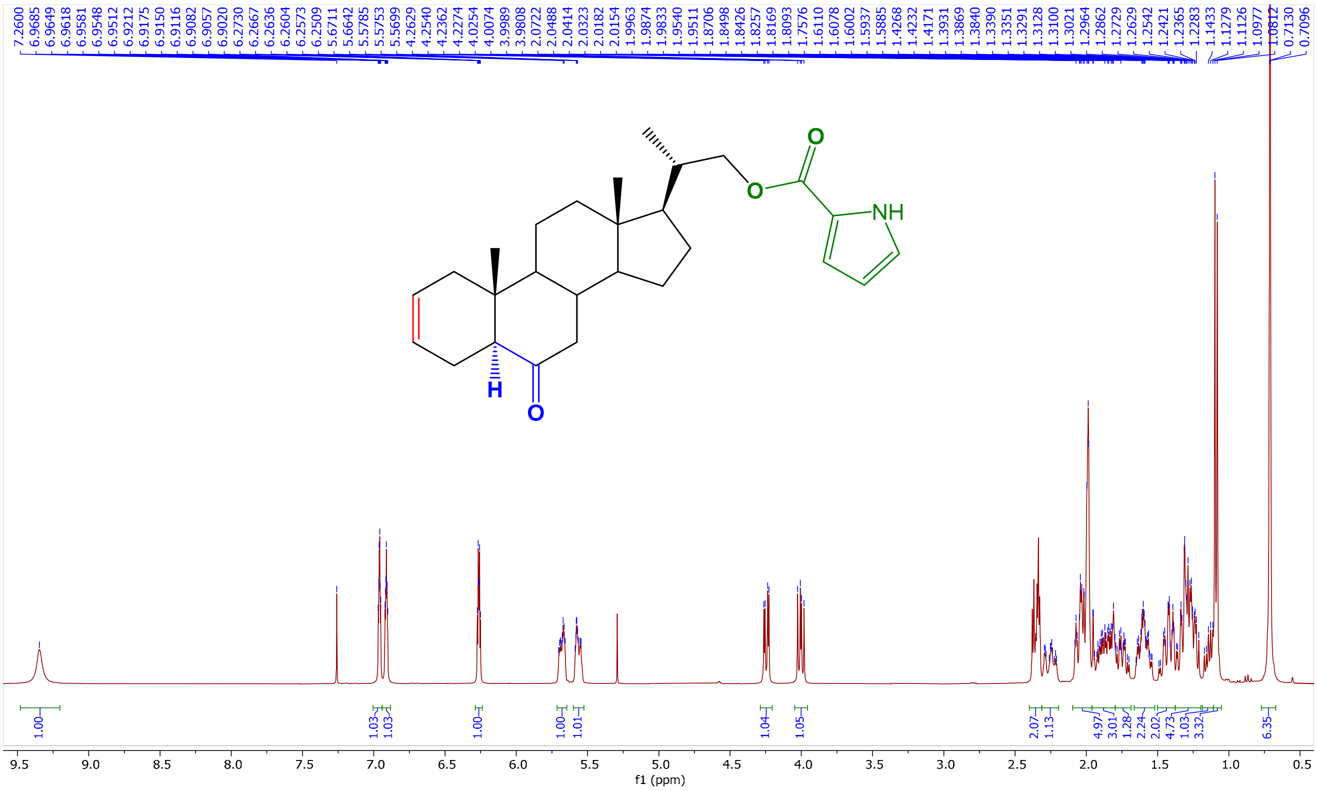

Figure S1. ^{1}H NMR spectrum of 6-Oxo-23,24-dinor-5α-Cholan-2-en-22-yl-1*H*-pyrrole-2-carboxylate (**33a**).

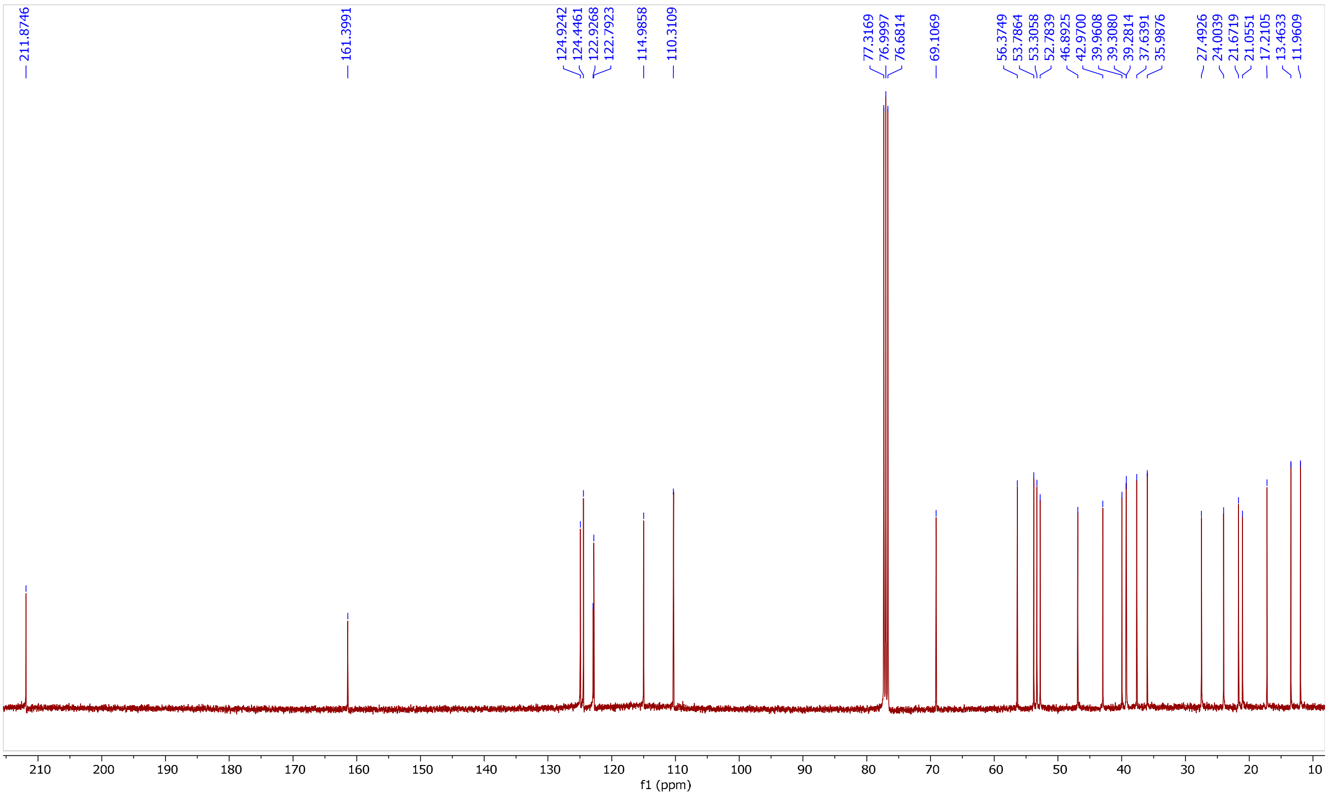

Figure S2. $^{13}C\{^{1}H\}$ NMR spectrum of 6-Oxo-23,24-dinor-5α-Cholan-2-en-22-yl-1*H*-pyrrole-2-carboxylate (**33a**).

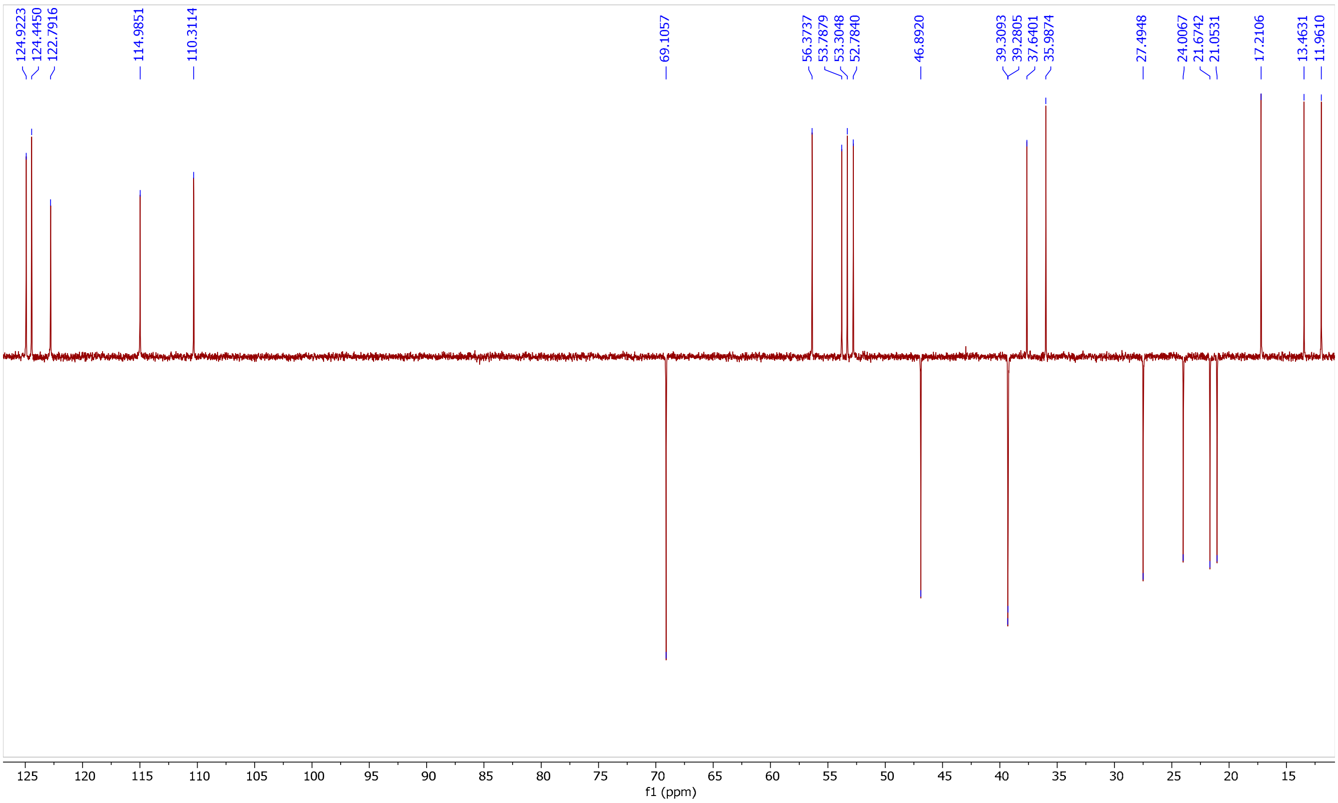

Figure S3. $^{13}C\{^{1}H\}$ DEPT-135 NMR spectrum of 6-Oxo-23,24-dinor-5*α*-Cholan-2-en-22-yl-1*H*-pyrrole-2-carboxylate (**33a**).

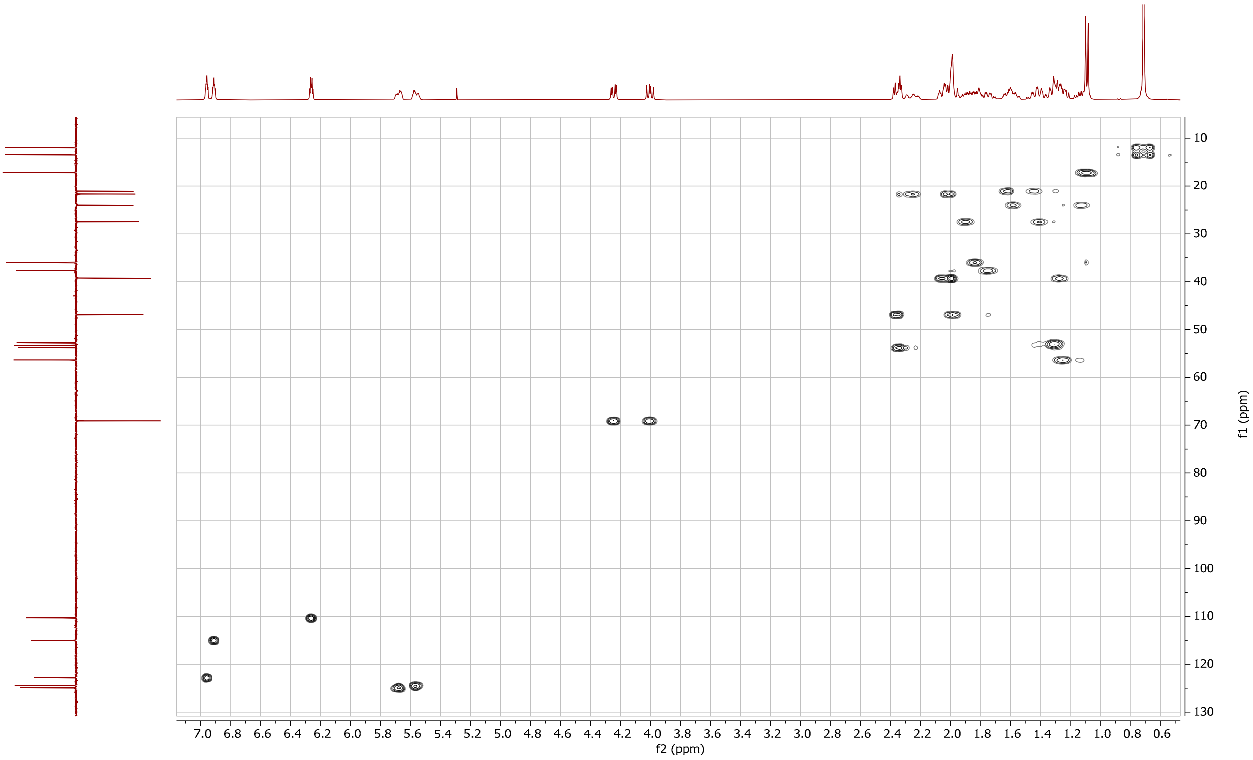

Figure S4. 2D ^{1}H-^{13}C HSQC spectrum of 6-Oxo-23,24-dinor-5*α*-Cholan-2-en-22-yl-1*H*-pyrrole-2-carboxylate (**33a**).

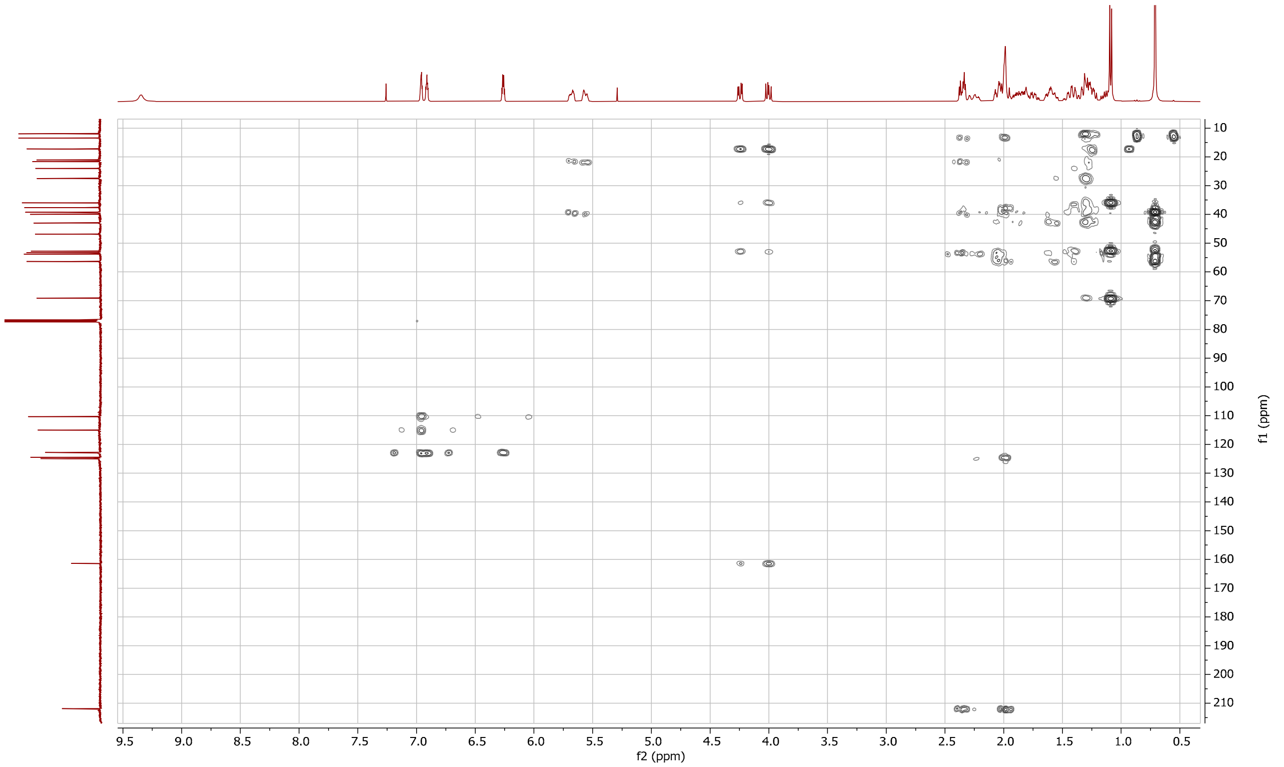

Figure S5. 2D ^{1}H-^{13}C HMBC spectrum of 6-Oxo-23,24-dinor-5α-Cholan-2-en-22-yl-1*H*-pyrrole-2-carboxylate (**33a**).

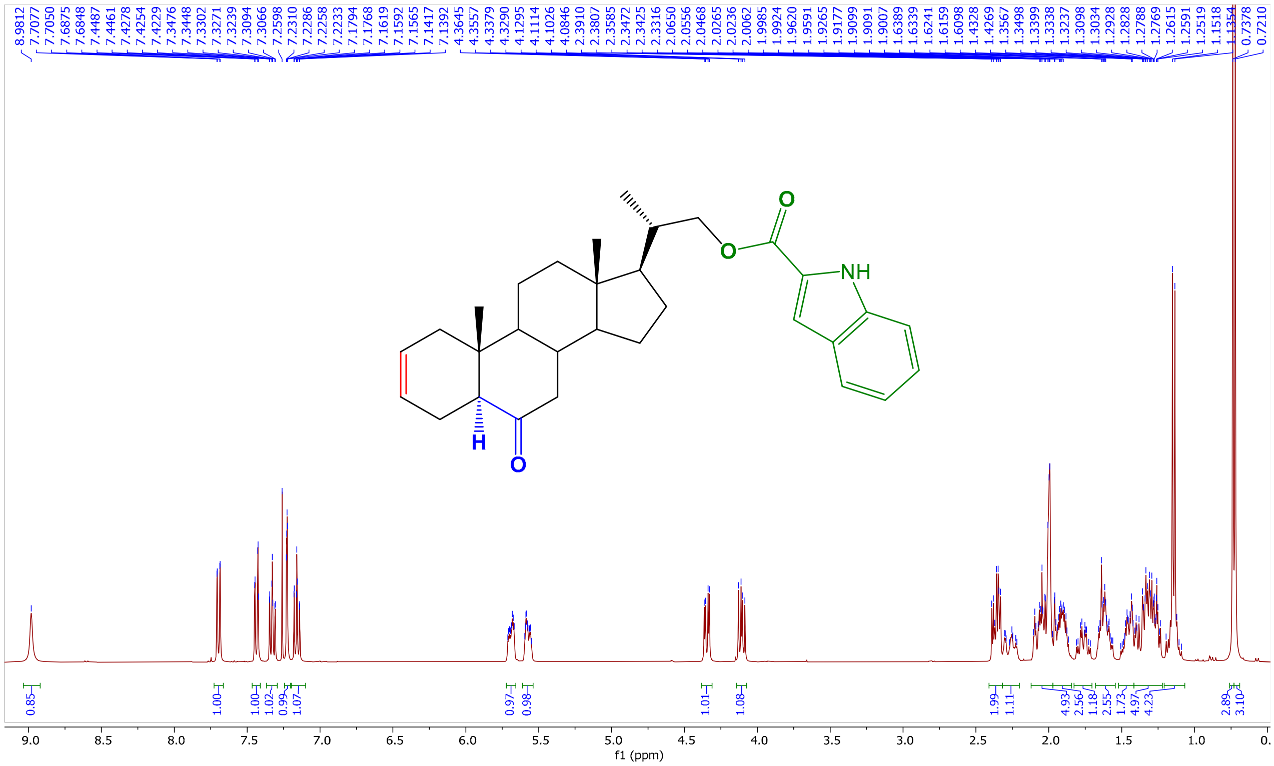

Figure S6. ^{1}H NMR spectrum of 6-Oxo-23,24-dinor-5α-Cholan-2-en-22-yl-1*H*-indole-2-carboxylate (**36a**)

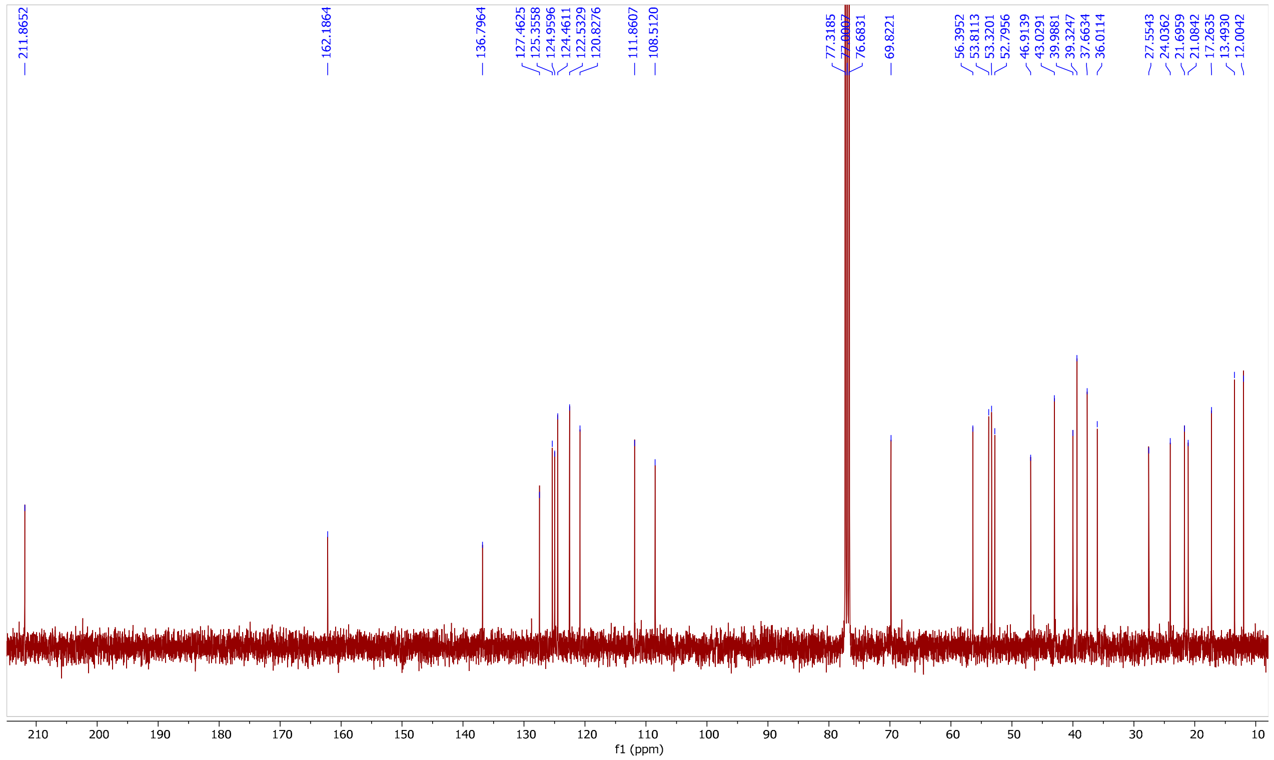

Figure S7. $^{13}C\{^{1}H\}$ NMR spectrum of 6-Oxo-23,24-dinor-5*α*-Cholan-2-en-22-yl-1*H*-indole-2-carboxylate (**36a**)

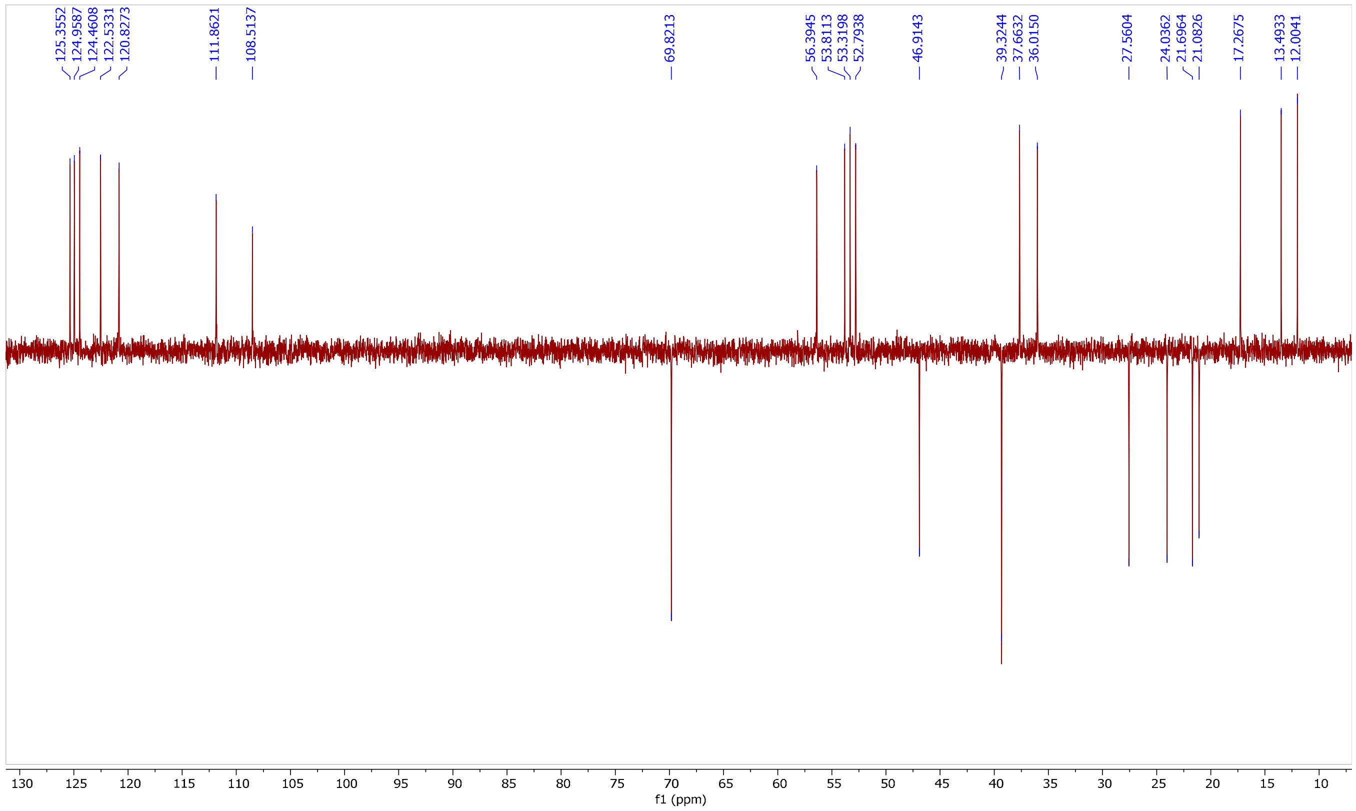

Figure S8. $^{13}C\{^{1}H\}$ DEPT-135 NMR spectrum of 6-Oxo-23,24-dinor-5*α*-Cholan-2-en-22-yl-1*H*-indole-2-carboxylate (**36a**)

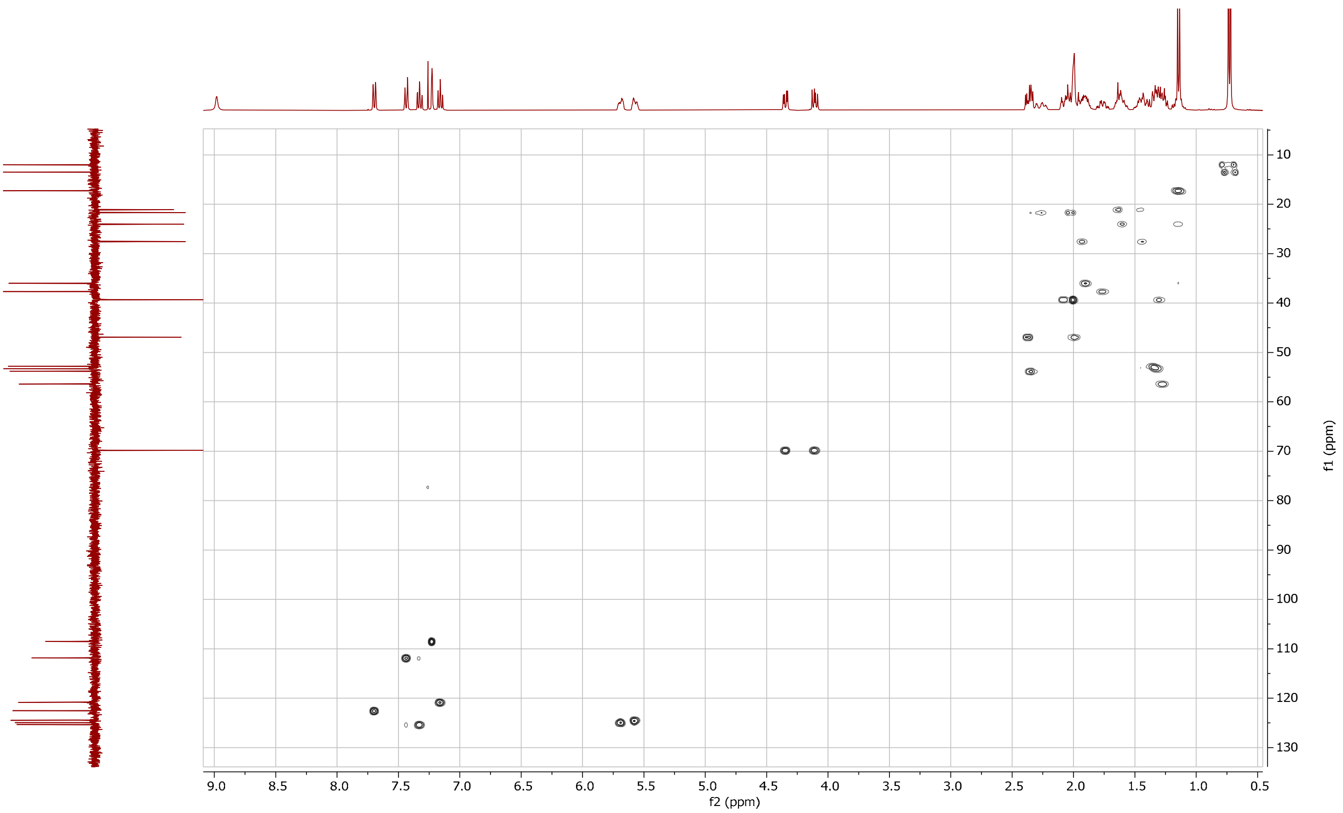

Figure S9. 2D ^{1}H-^{13}C HSQC spectrum of 6-Oxo-23,24-dinor-5α-Cholan-2-en-22-yl-1*H*-indole-2-carboxylate (**36a**)

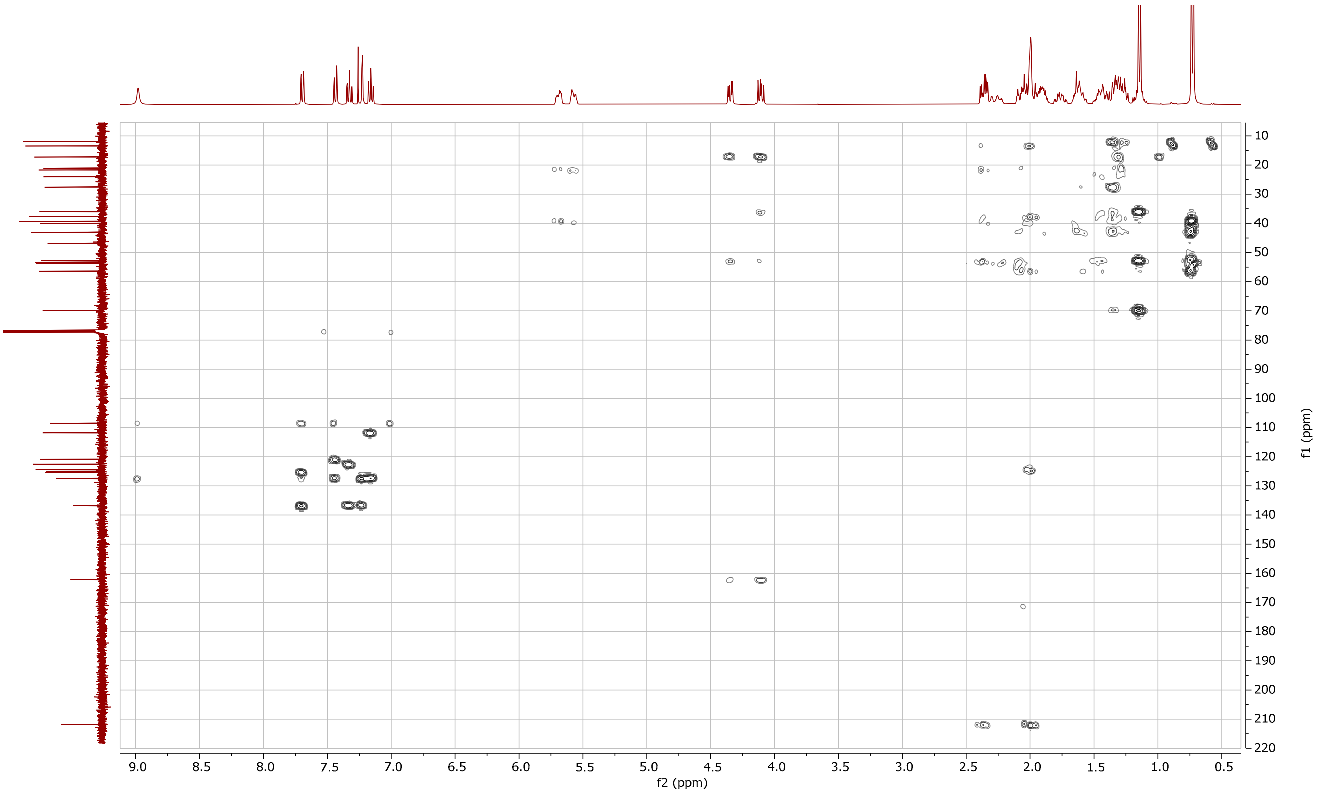

Figure S10. 2D ^{1}H-^{13}C HMBC spectrum of 6-Oxo-23,24-dinor-5α-Cholan-2-en-22-yl-1*H*-indole-2-carboxylate (**36a**)

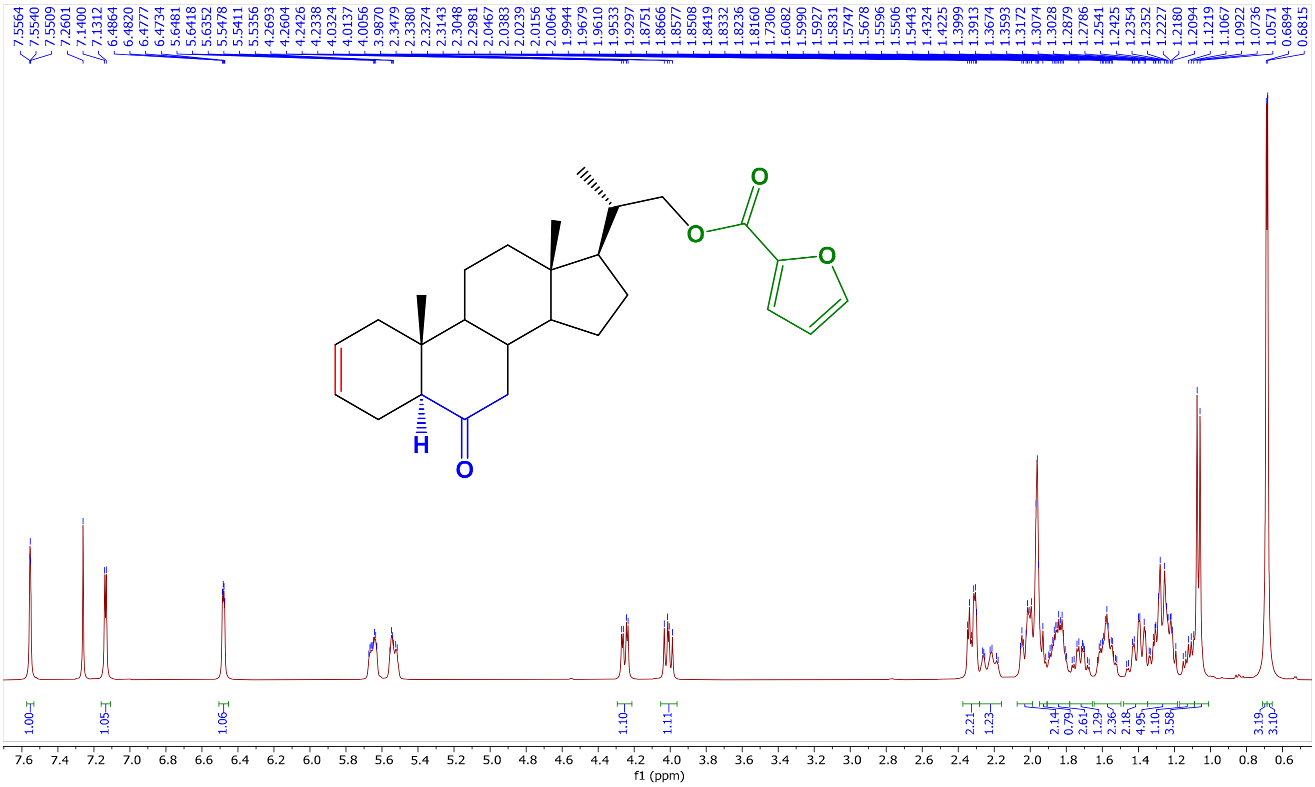

Figure S11. ^{1}H NMR spectrum of 6-Oxo-23,24-dinor-5α-Cholan-2-en-22-yl-furan-2-carboxylate (**34a**)

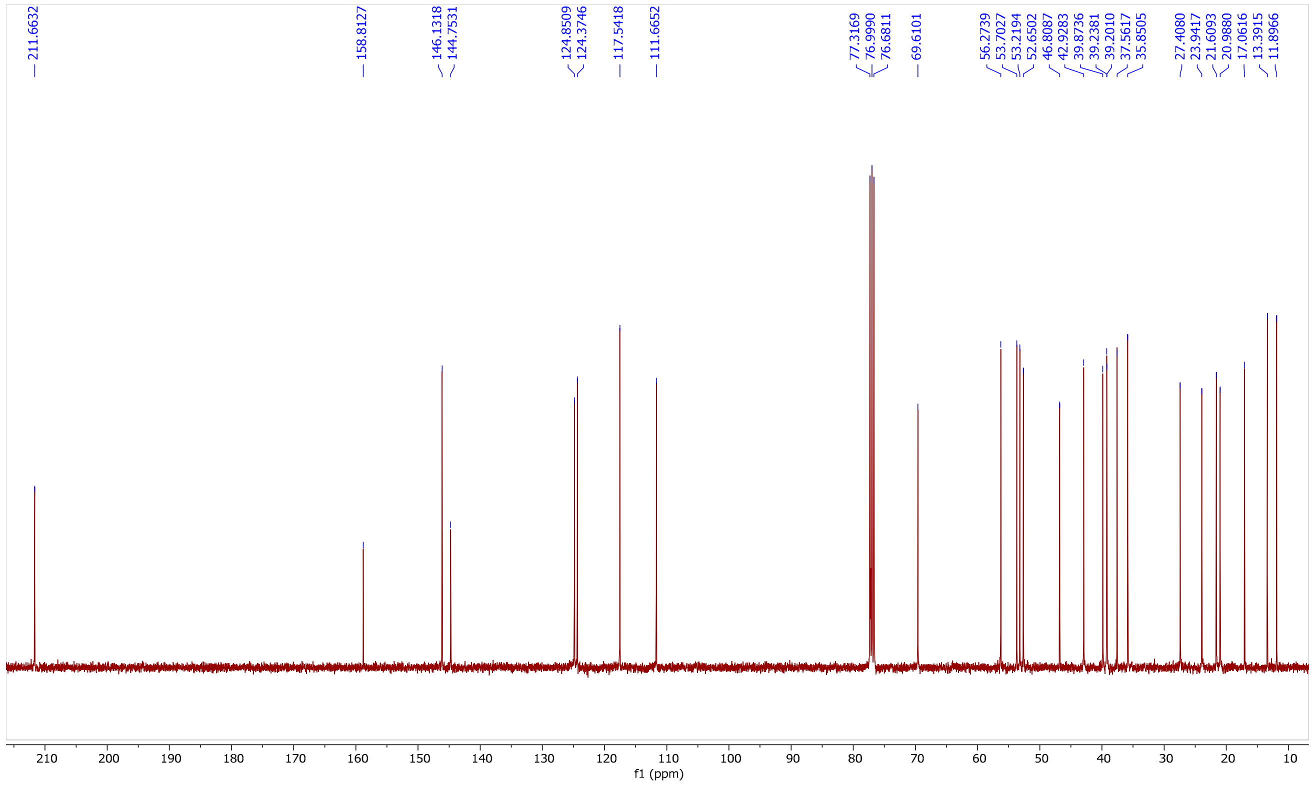

Figure S12. ^{13}C{^{1}H} NMR spectrum of 6-Oxo-23,24-dinor-5α-Cholan-2-en-22-yl-furan-2-carboxylate (**34a**)

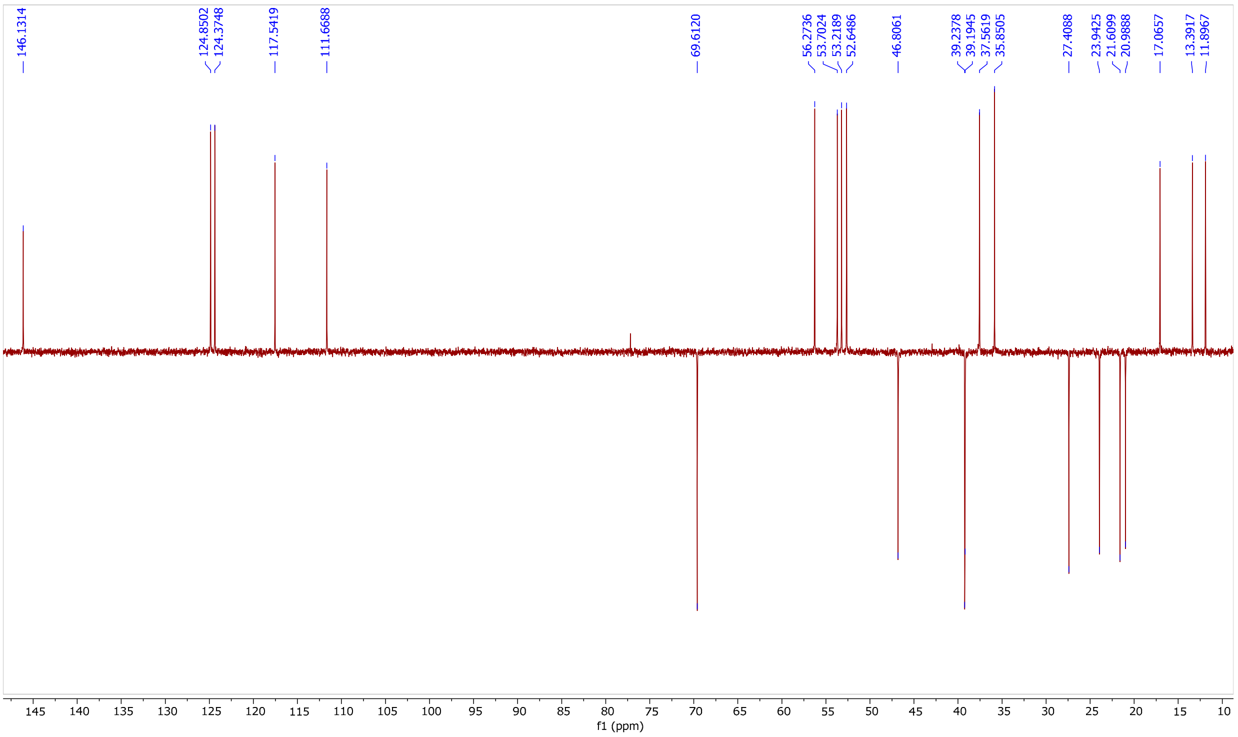

Figure S13. $^{13}C\{^{1}H\}$ DEPT-135 NMR spectrum of 6-Oxo-23,24-dinor-5α-Cholan-2-en-22-yl-furan-2-carboxylate (**34a**)

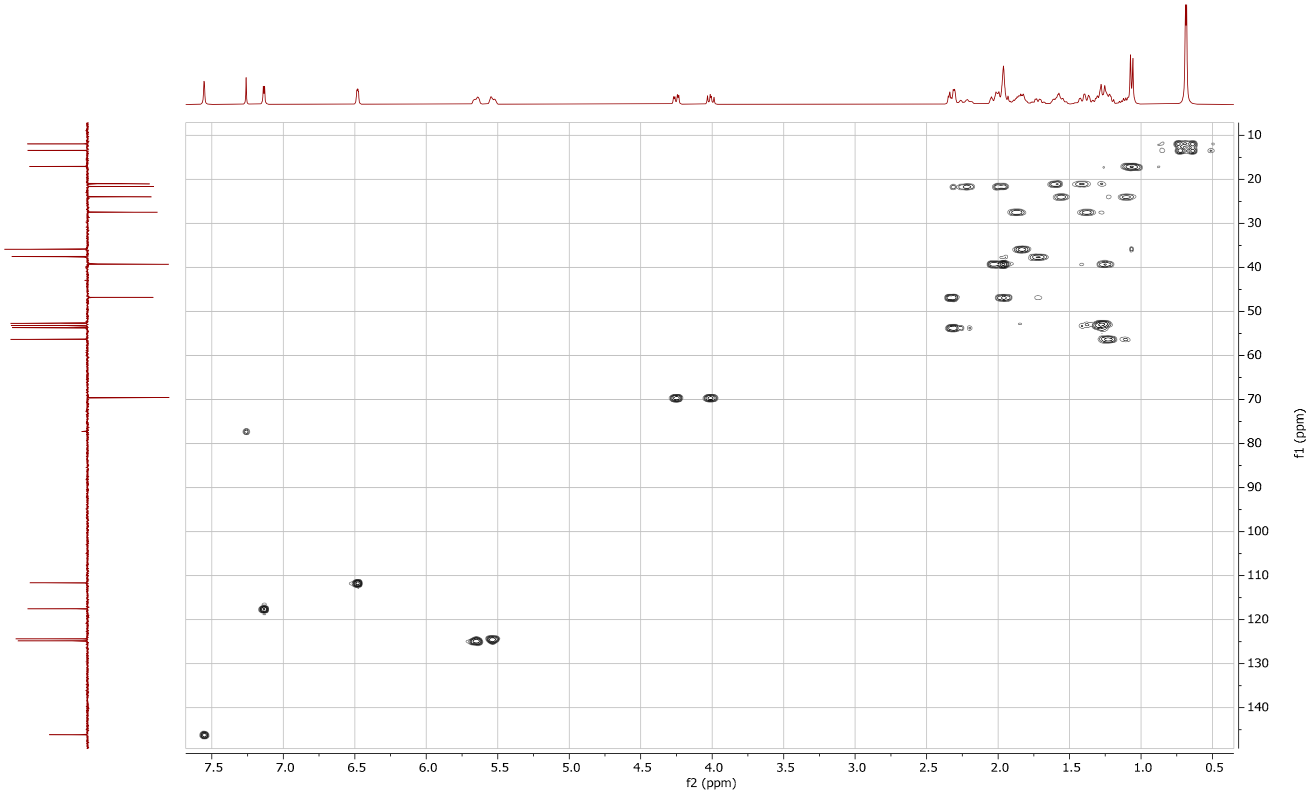

Figure S14. 2D ^{1}H-^{13}C HSQC spectrum of 6-Oxo-23,24-dinor-5α-Cholan-2-en-22-yl-furan-2-carboxylate (**34a**)

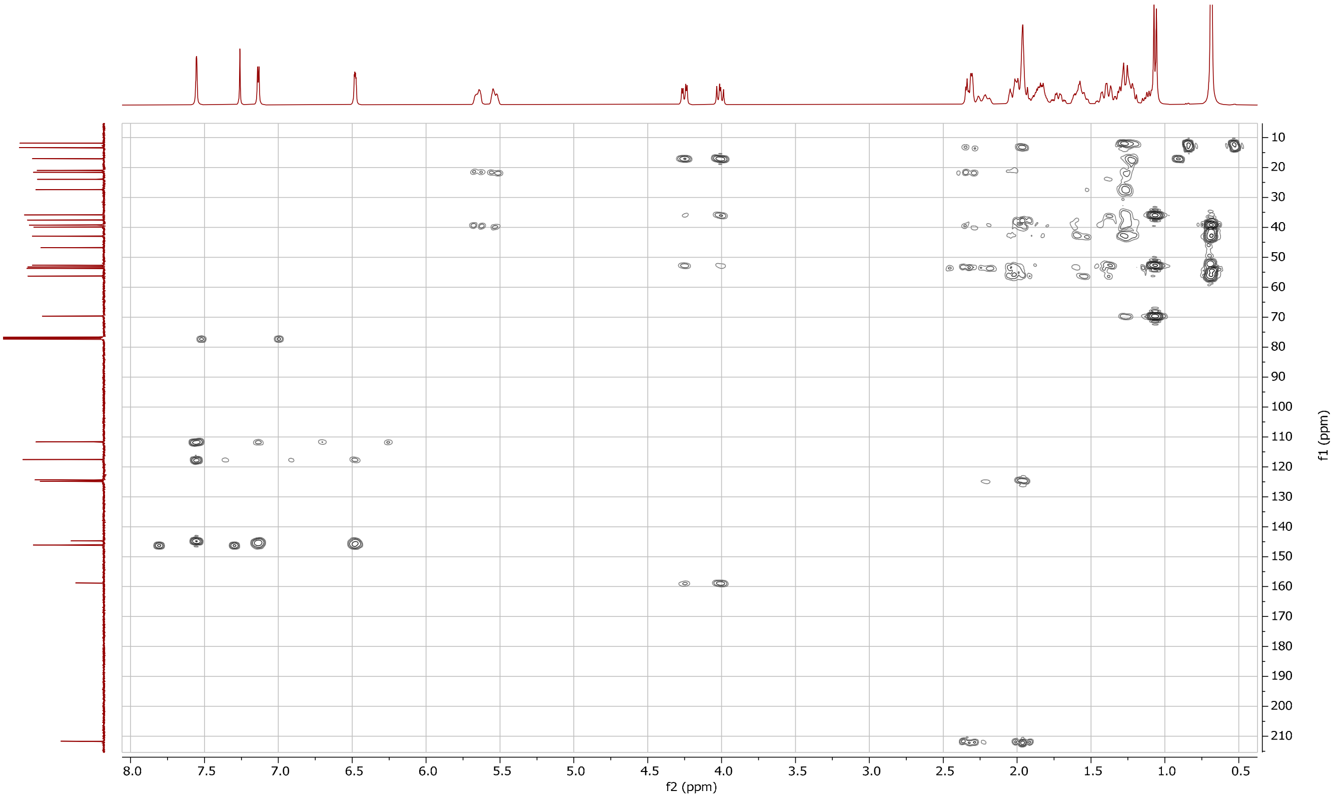

Figure S15. 2D ^{1}H-^{13}C HMBC spectrum of 6-Oxo-23,24-dinor-5α-Cholan-2-en-22-yl-furan-2-carboxylate (**34a**)

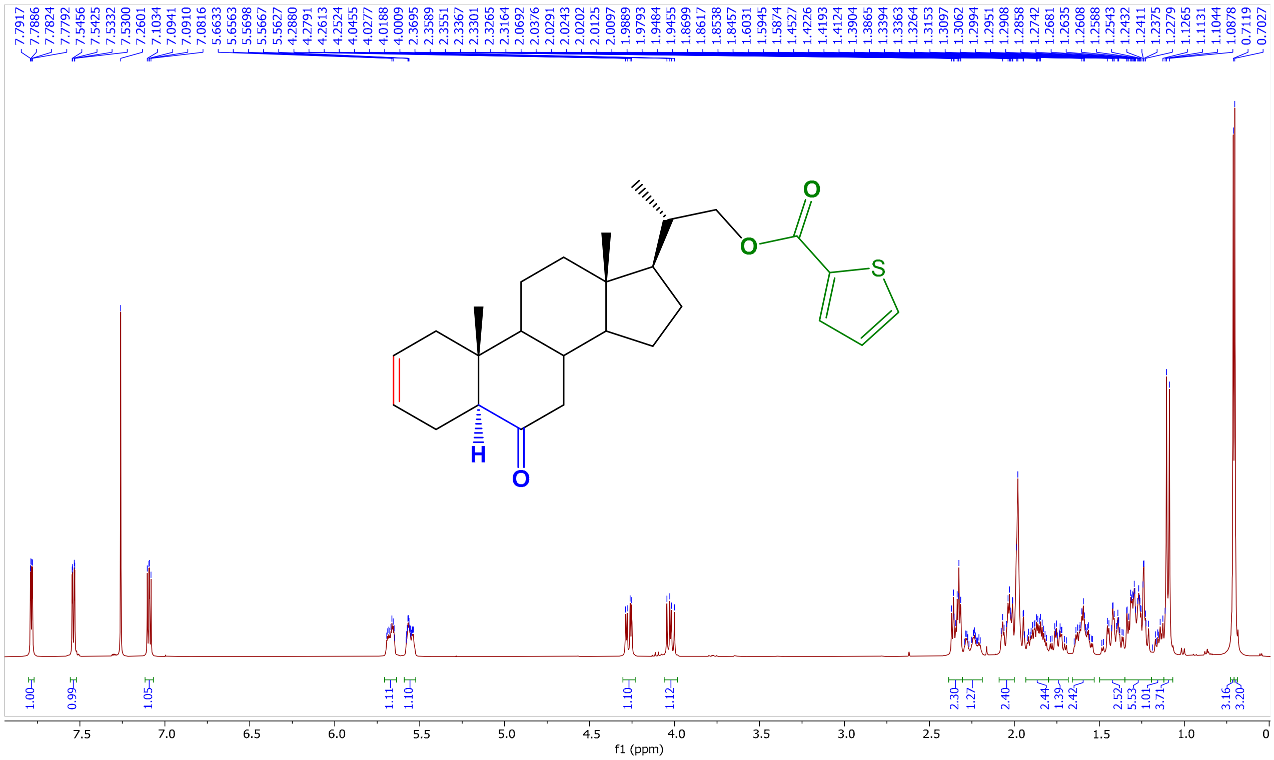

Figure S16. ^{1}H NMR spectrum of 6-Oxo-23,24-dinor-5α-Cholan-2-en-22-yl-thiophene-2-carboxylate (**35a**)

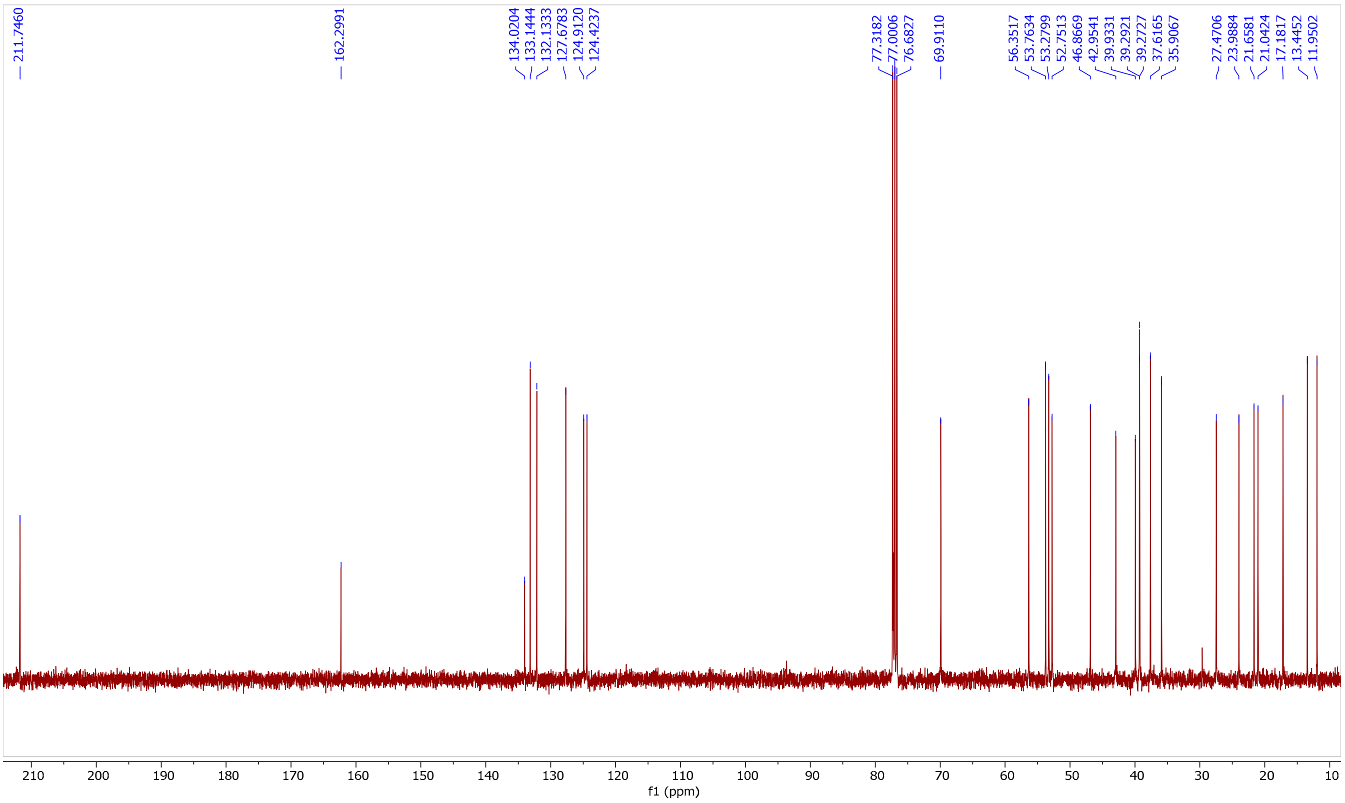

Figure S17. $^{13}C\{^{1}H\}$ NMR spectrum of 6-Oxo-23,24-dinor-5α-Cholan-2-en-22-yl-thiophene-2-carboxylate (**35a**)

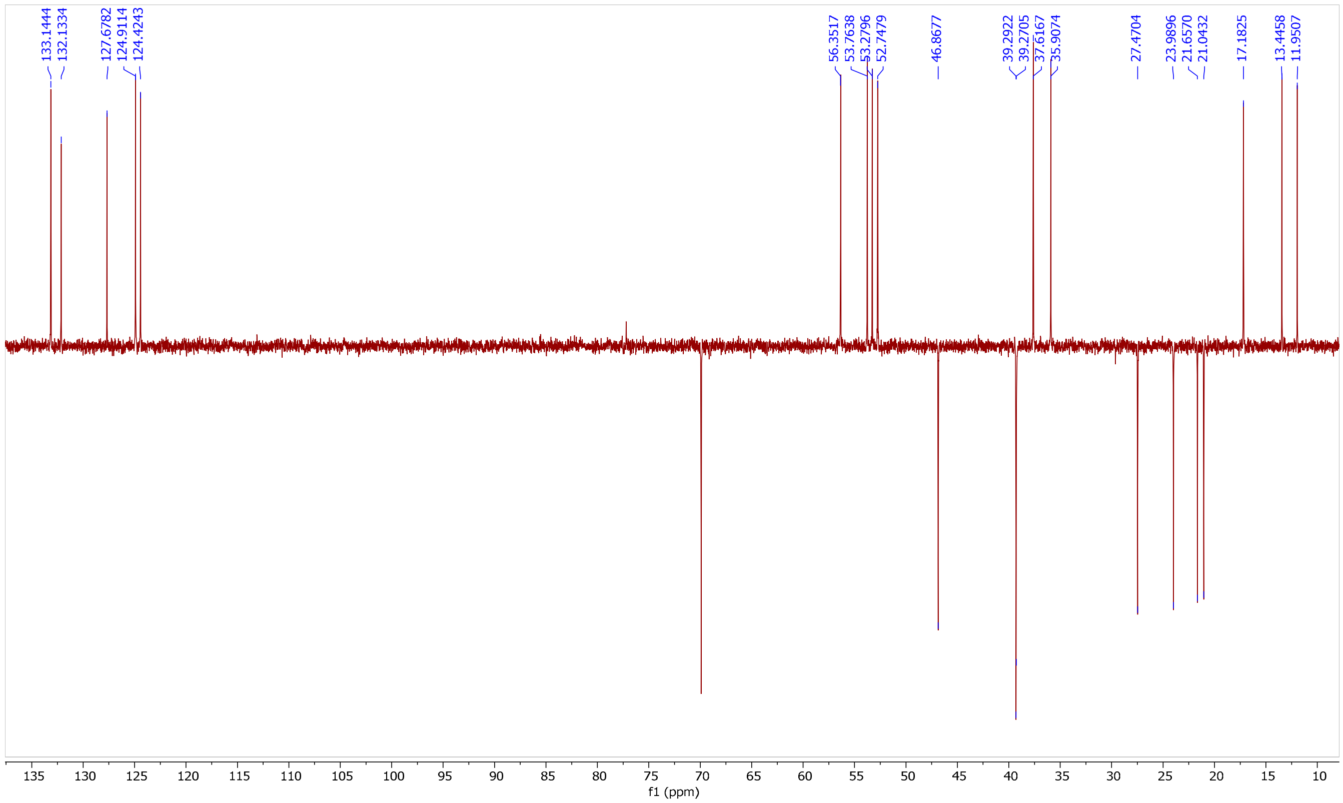

Figure S18. $^{13}C\{^{1}H\}$ DEPT-135 NMR spectrum of 6-Oxo-23,24-dinor-5α-Cholan-2-en-22-yl-thiophene-2-carboxylate (**35a**)

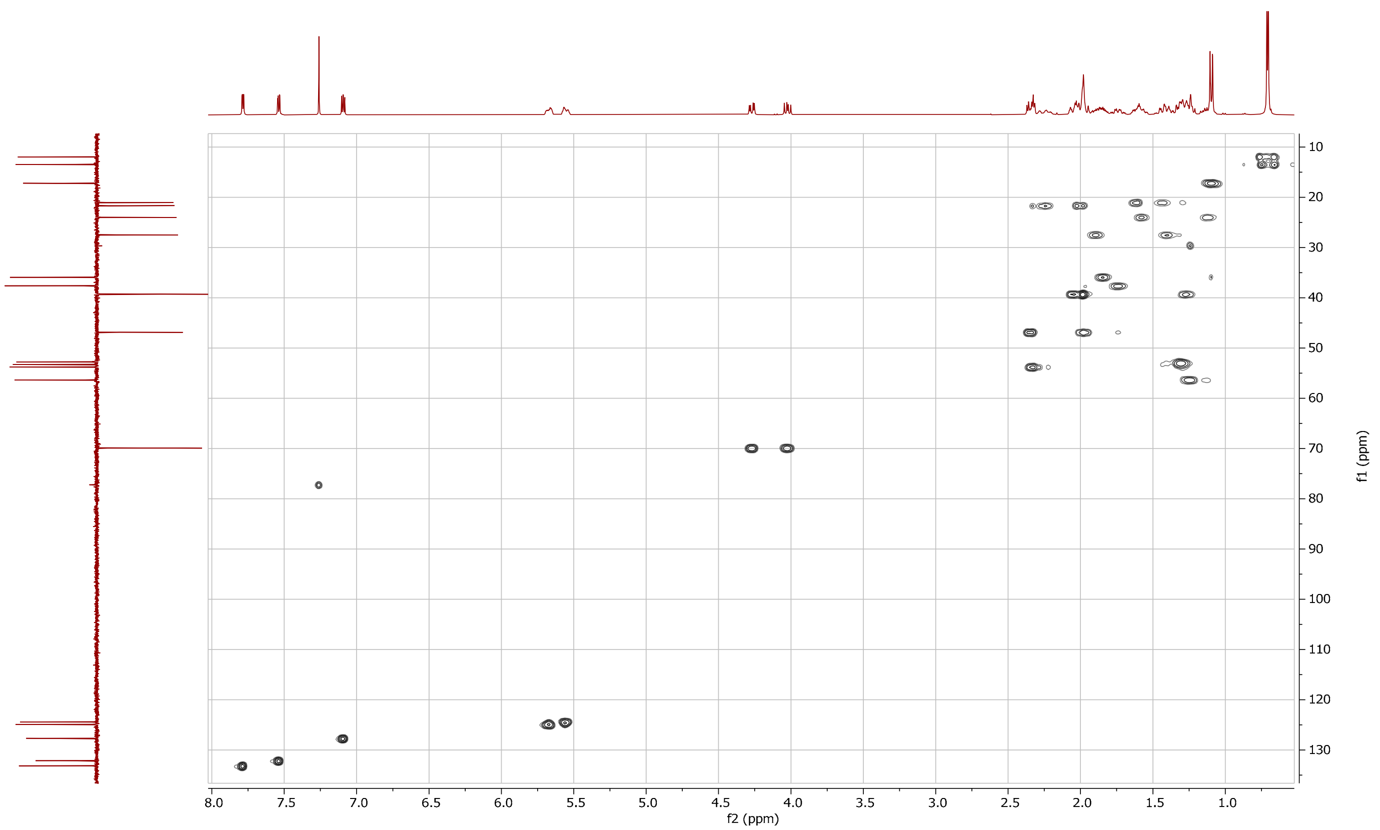

Figure S19. 2D ^{1}H-^{13}C HSQC spectrum of 6-Oxo-23,24-dinor-5α-Cholan-2-en-22-yl-thiophene-2-carboxylate (**35a**)

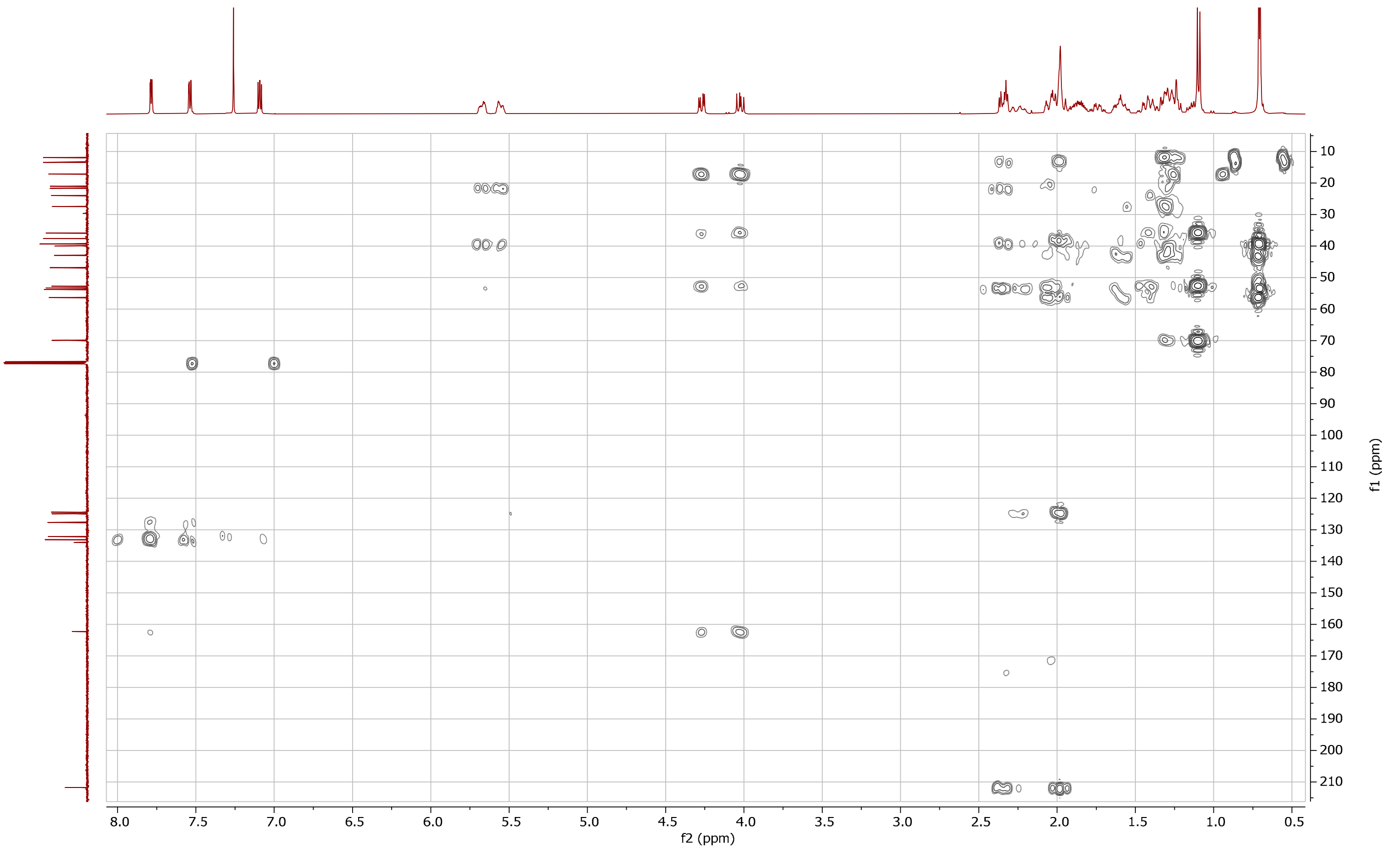

Figure S20. 2D ^{1}H-^{13}C HMBC spectrum of 6-Oxo-23,24-dinor-5α-Cholan-2-en-22-yl-thiophene-2-carboxylate (**35a**)

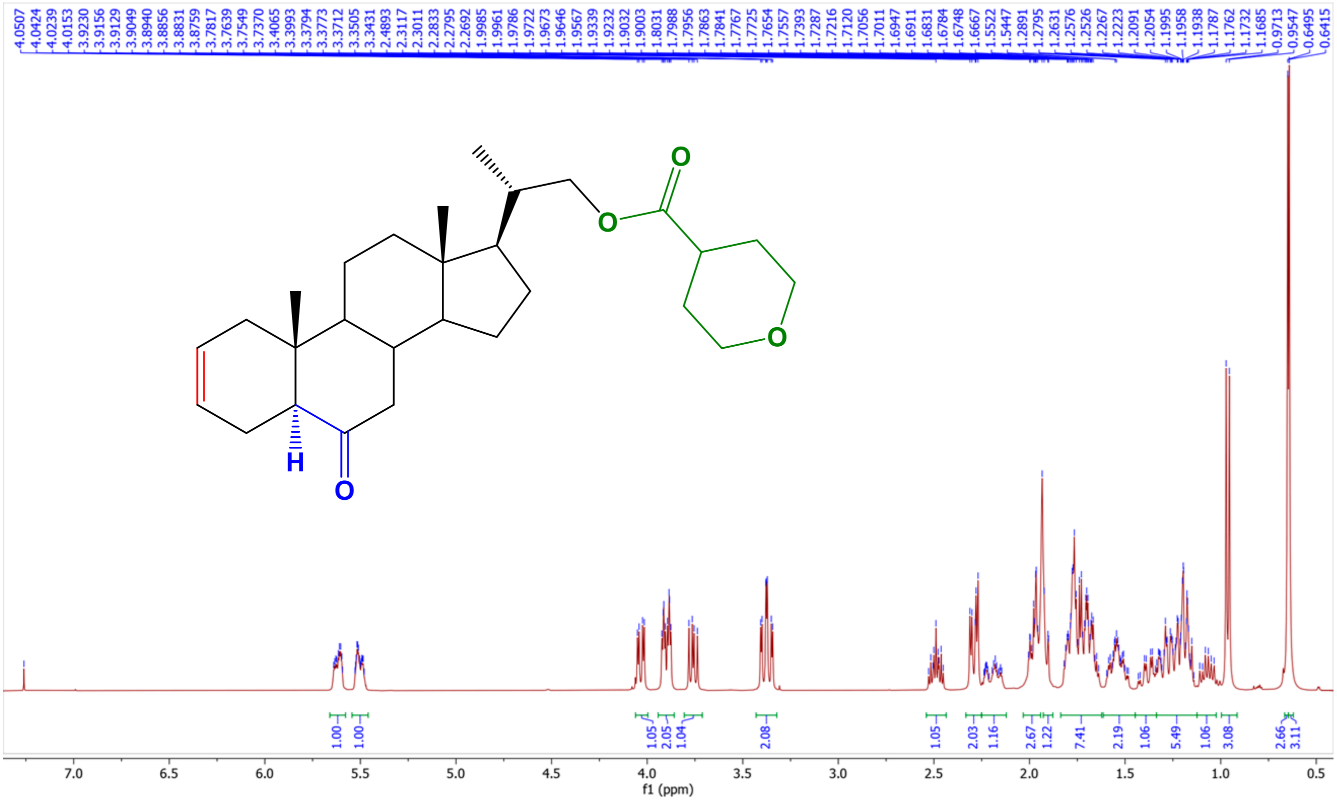

Figure S21. ^{1}H NMR spectrum of 6-Oxo-23,24-dinor-5α-Cholan-2-en-22-yl-tetrahydro-2*H*-pyran-4-carbocarboxylate (**37a**)

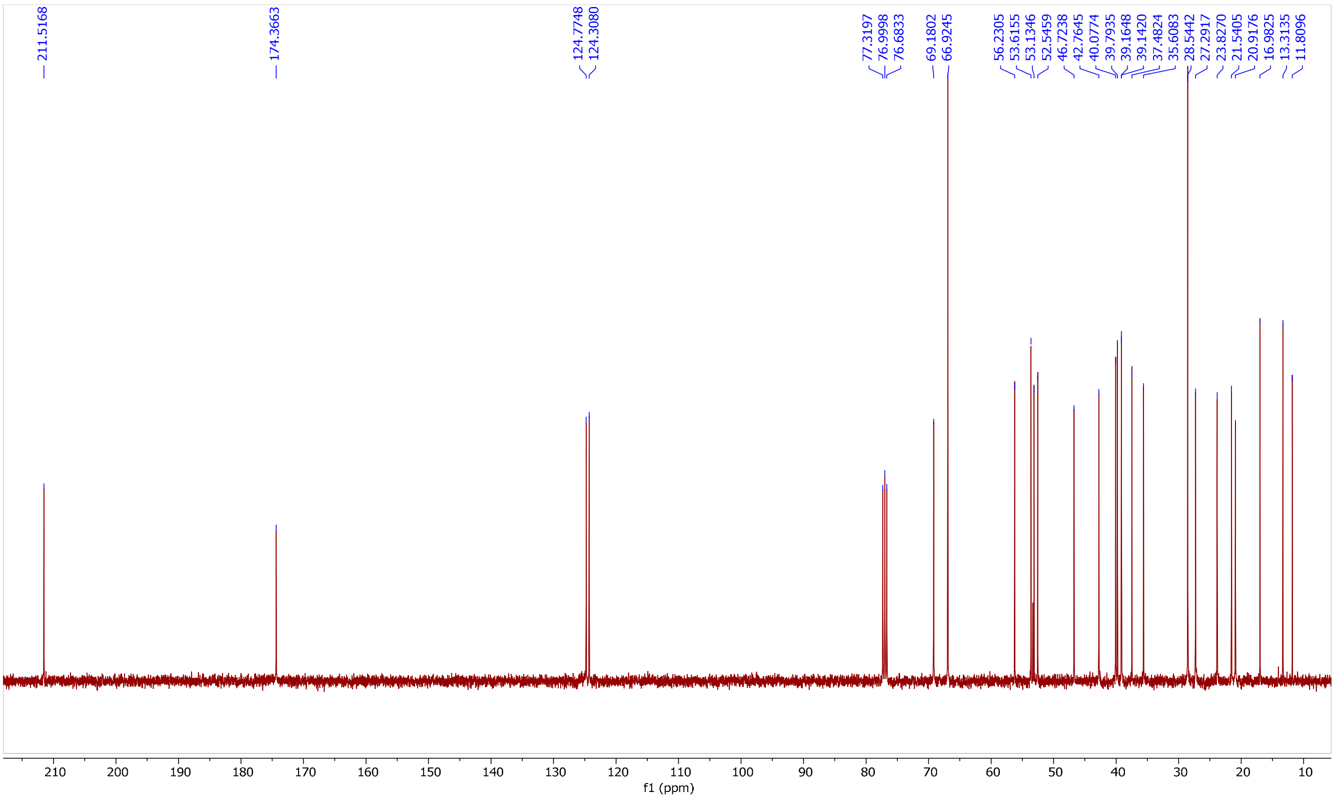

Figure S22. $^{13}C\{^{1}H\}$ NMR spectrum of 6-Oxo-23,24-dinor-5α-Cholan-2-en-22-yl-tetrahydro-2*H*-pyran-4-carbocarboxylate (**37a**)

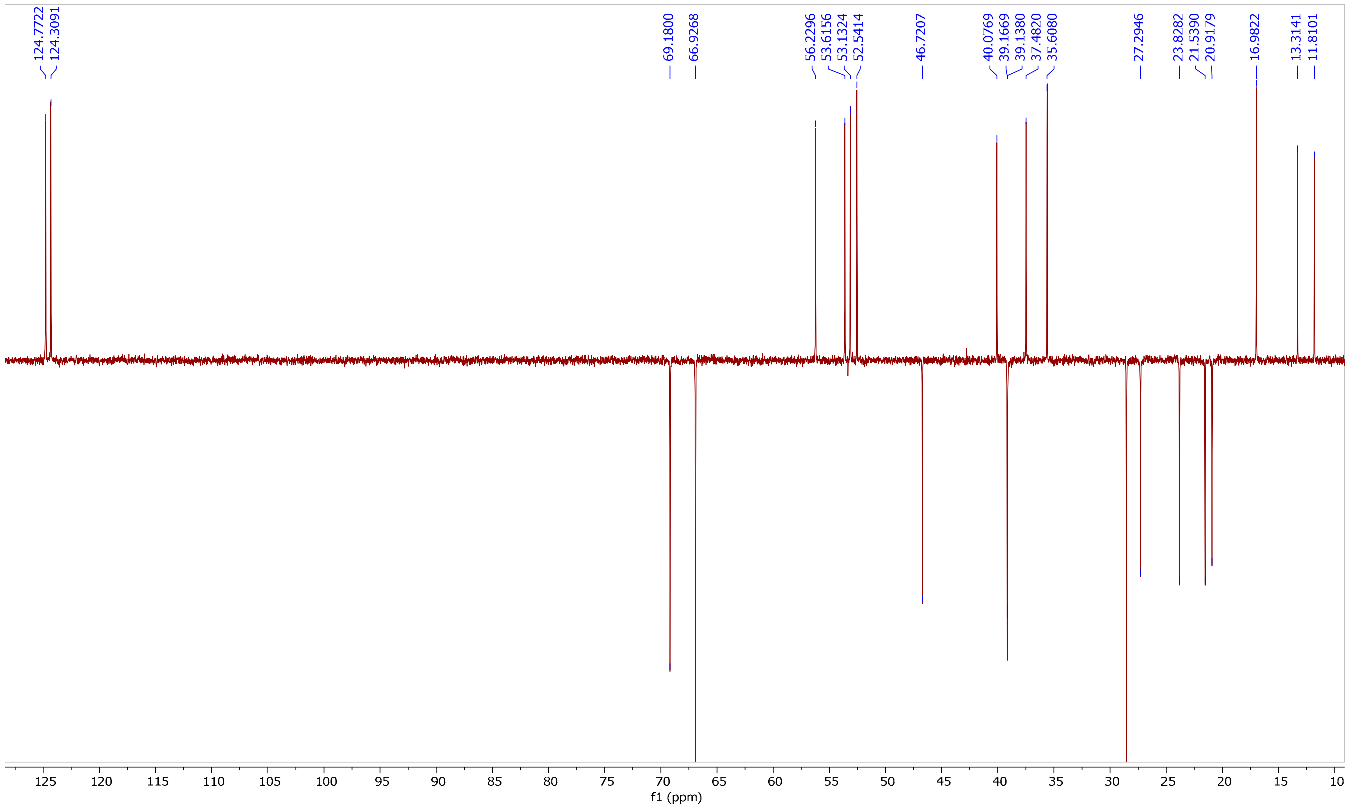

Figure S23. $^{13}C\{^{1}H\}$ DEPT-135 NMR spectrum of 6-Oxo-23,24-dinor-5α-Cholan-2-en-22-yl-tetrahydro-2*H*-pyran-4-carbocarboxylate (**37a**)

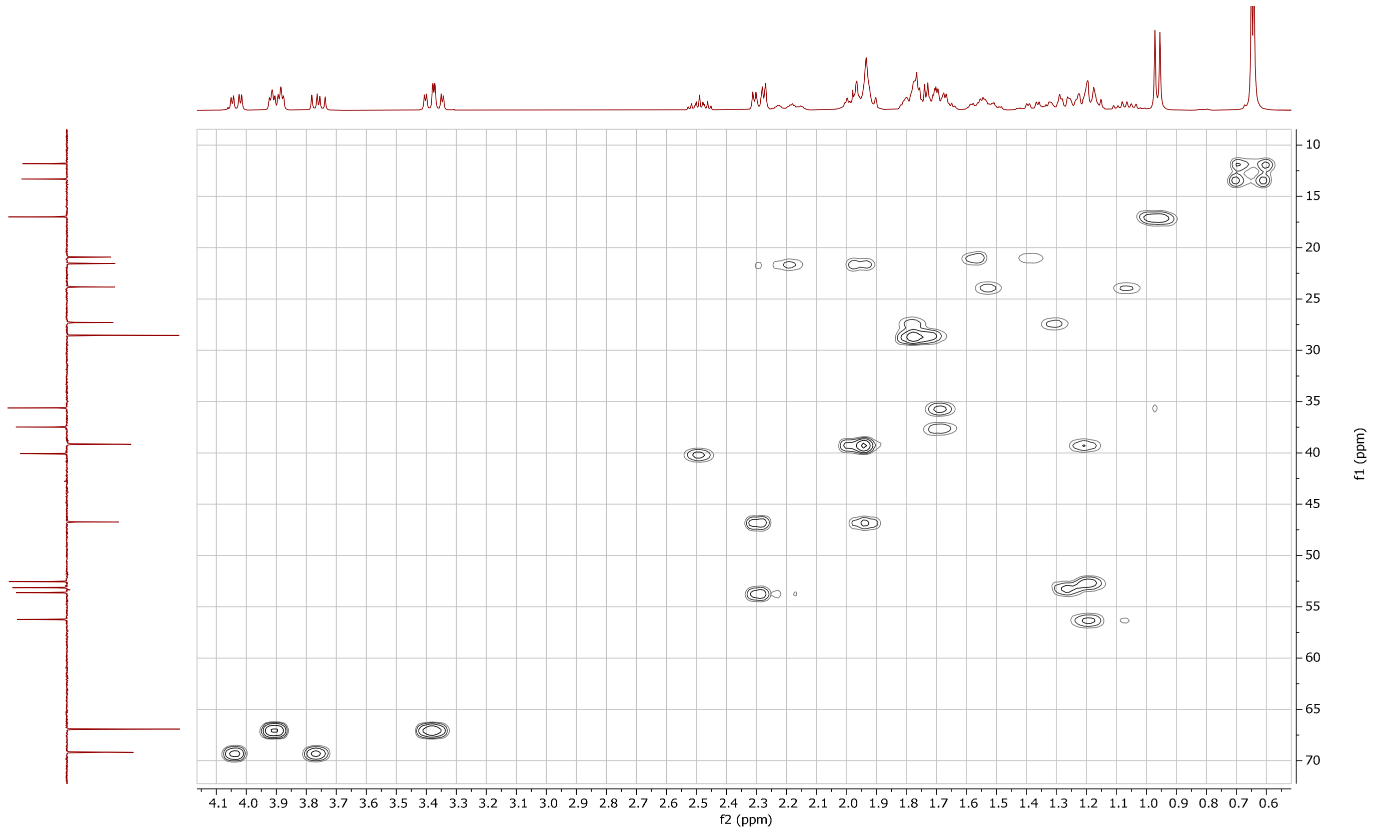

Figure S24. 2D ^{1}H-^{13}C HSQC spectrum of 6-Oxo-23,24-dinor-5α-Cholan-2-en-22-yl-tetrahydro-2*H*-pyran-4-carbocarboxylate (**37a**)

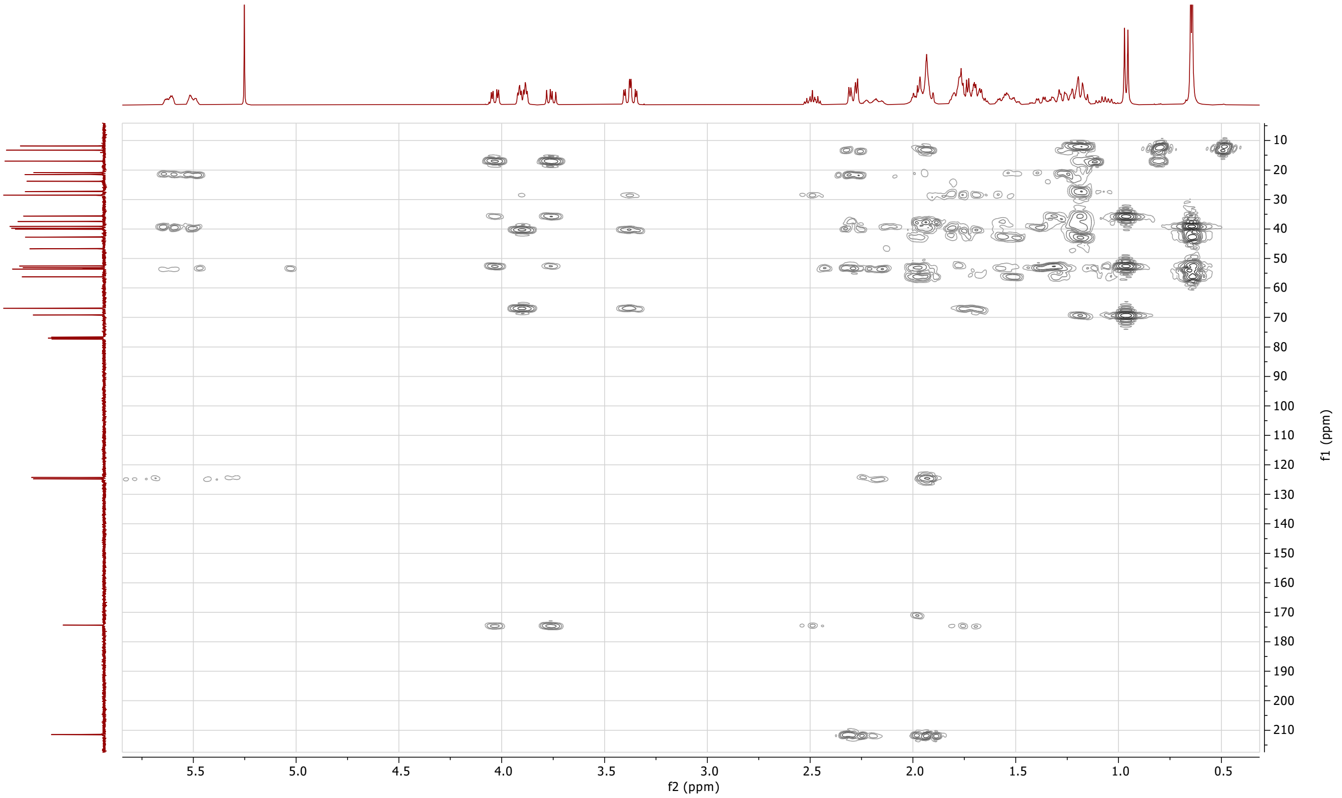

Figure S25. 2D ^{1}H-^{13}C HMBC spectrum of 6-Oxo-23,24-dinor-5*α*-Cholan-2-en-22-yl-tetrahydro-2*H*-pyran-4-carbocarboxylate (**37a**)

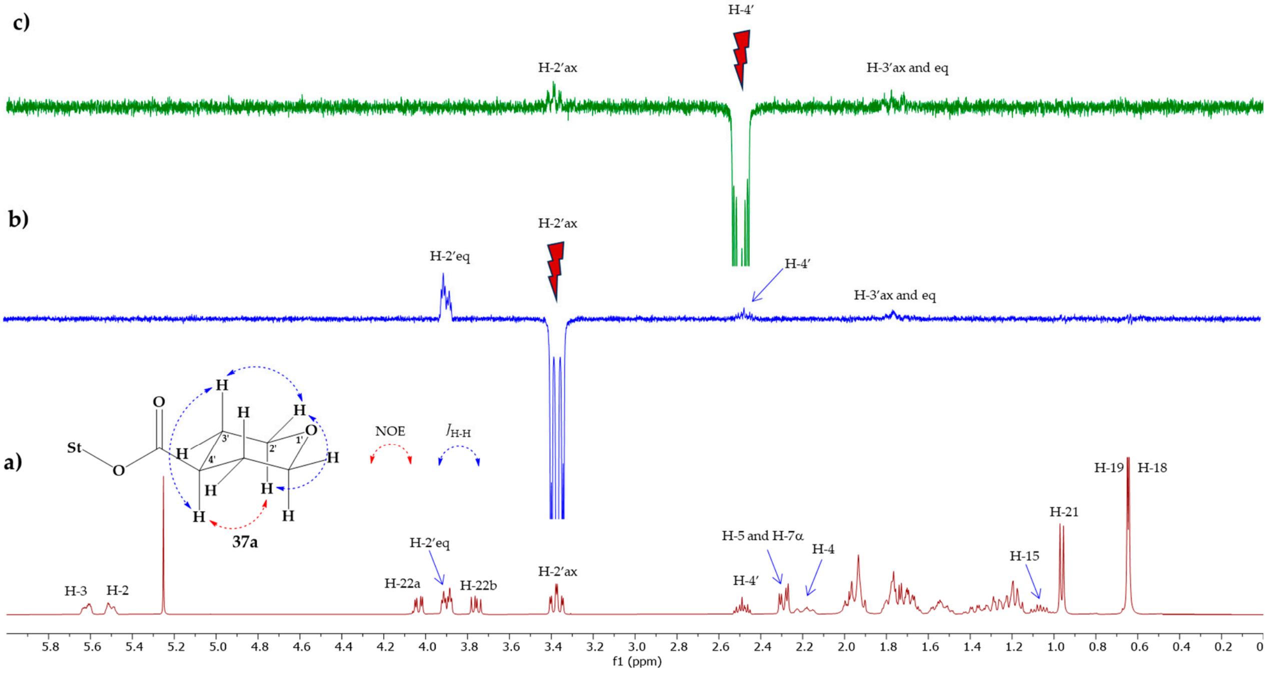

Figure S26. 1D selective NOESY NMR spectrum 6-Oxo-23,24-dinor-5*α*-Cholan-2-en-22-yl-tetrahydro-2*H*-pyran-4-carbocarboxylate (**37a**)

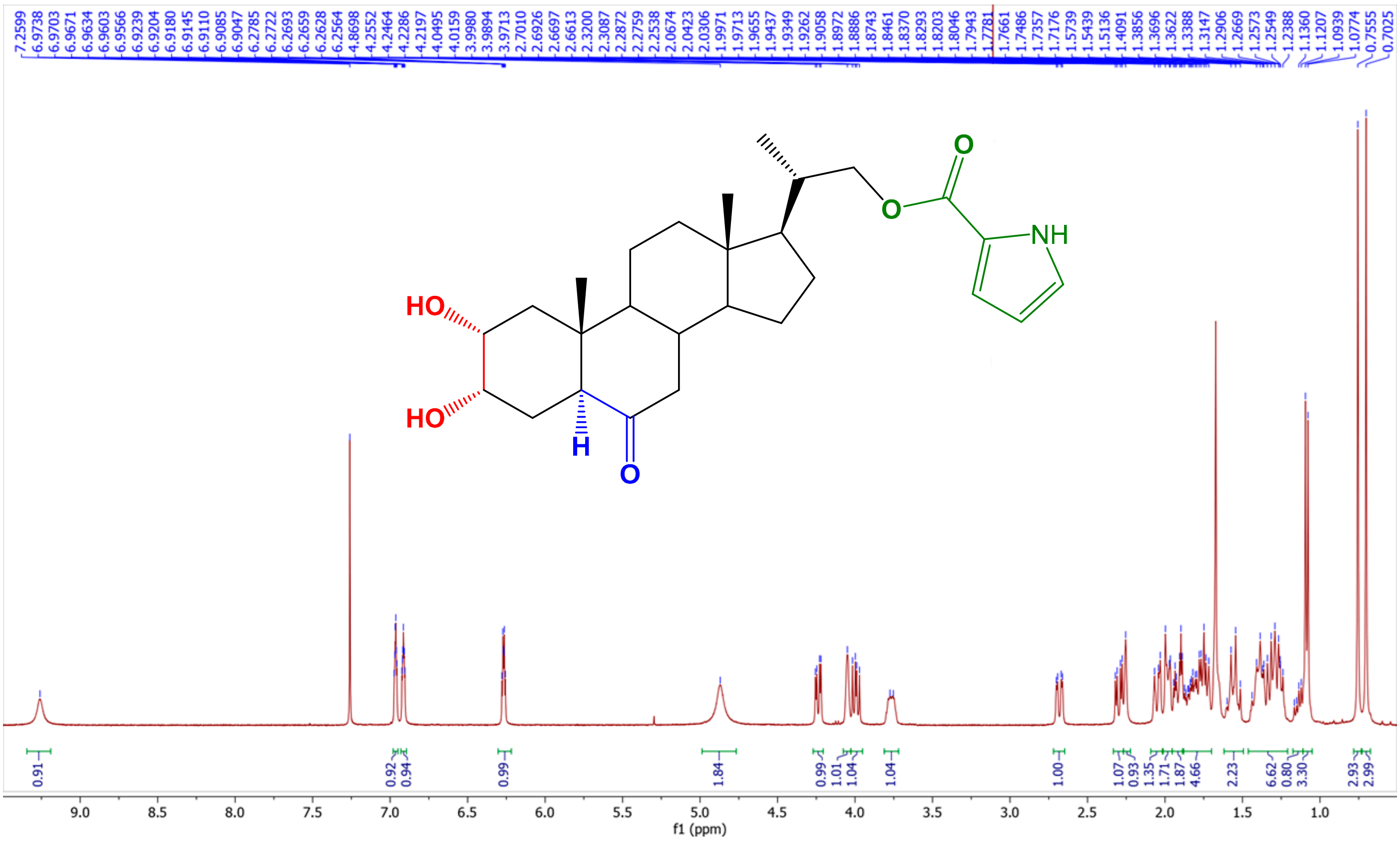

Figure S27. ^{1}H NMR spectrum of 2*α*,3*α*-Dihydroxy-6-oxo-23,24-dinor-5*α*-cholan-22-yl-1*H*-pyrrole-2-carboxylate (**33**)

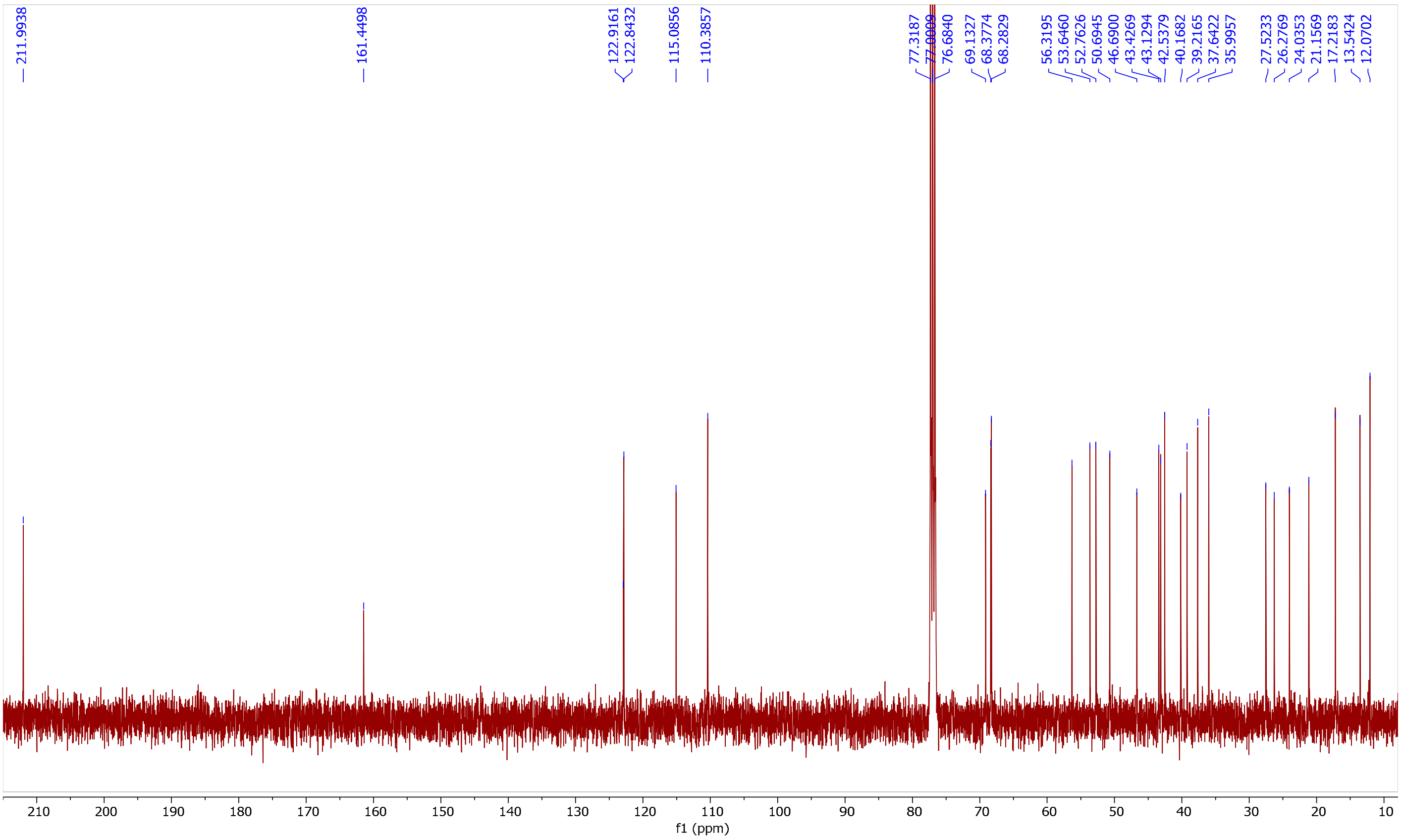

Figure S28. $^{13}C\{^{1}H\}$ NMR spectrum of 2*α*,3*α*-Dihydroxy-6-oxo-23,24-dinor-5*α*-cholan-22-yl-1*H*-pyrrole-2-carboxylate (**33**)

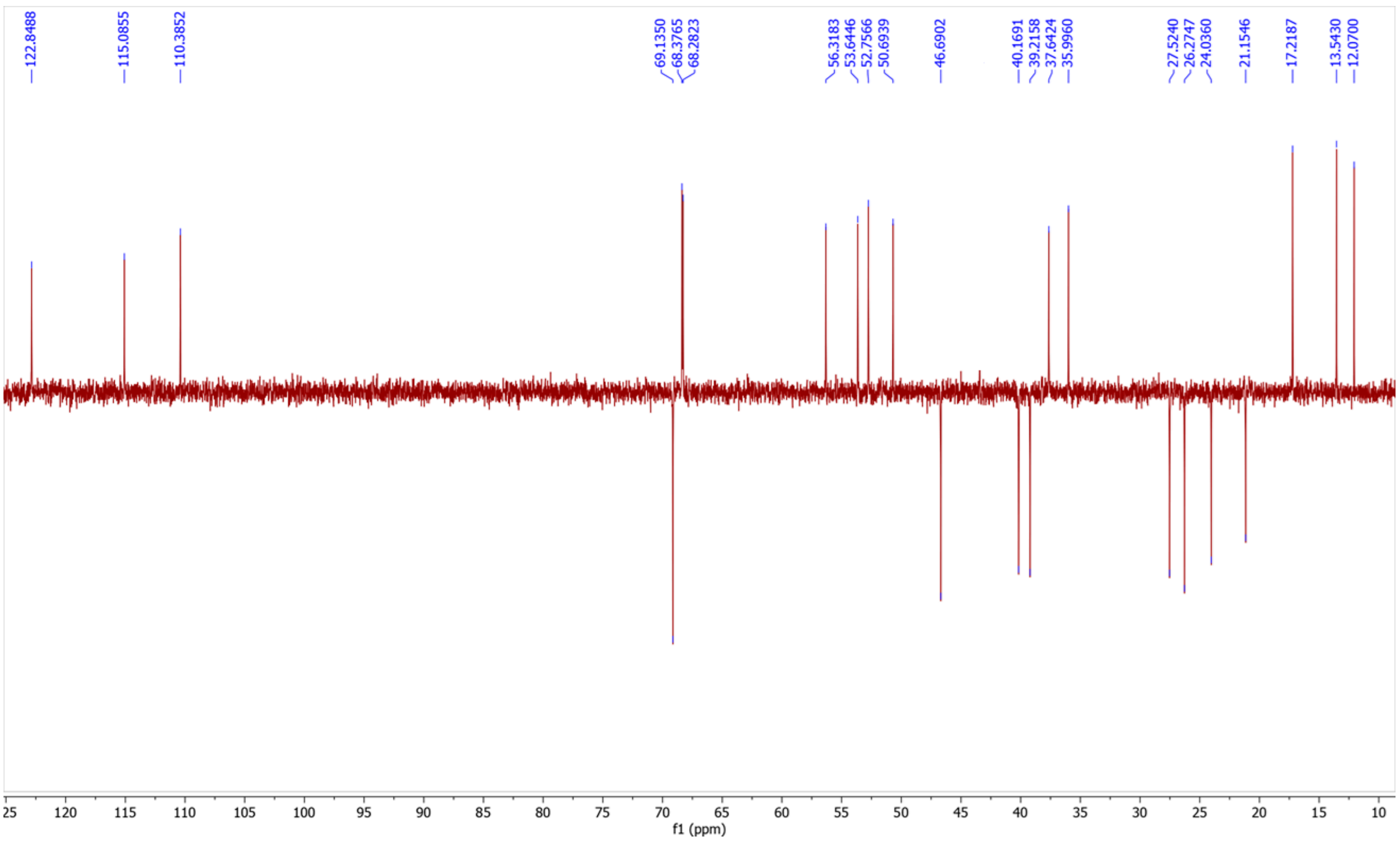

Figure S29. $^{13}C\{^{1}H\}$ DEPT-135 NMR spectrum of 2*α*,3*α*-Dihydroxy-6-oxo-23,24-dinor-5*α*-cholan-22-yl-1*H*-pyrrole-2-carboxylate (**33**)

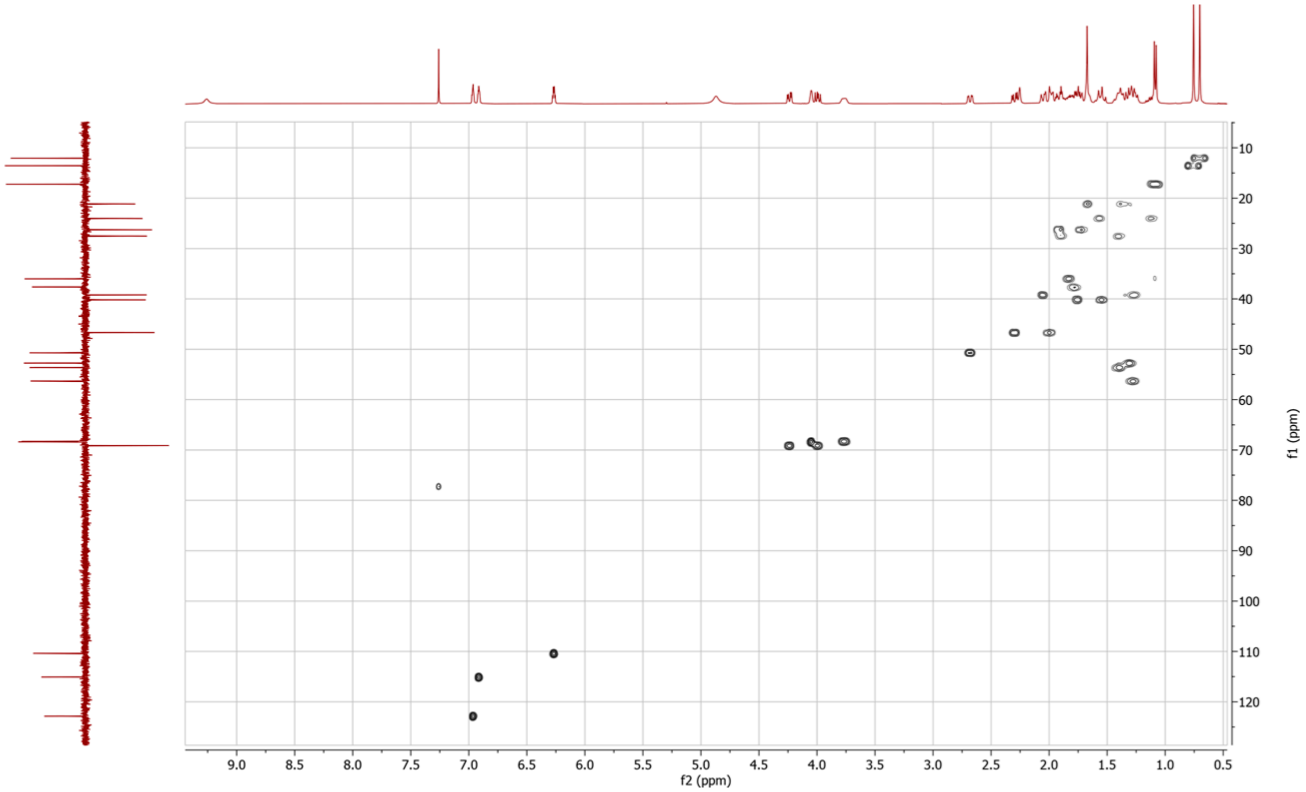

Figure S30. 2D ^{1}H-^{13}C HSQC spectrum of 2*α*,3*α*-Dihydroxy-6-oxo-23,24-dinor-5*α*-cholan-22-yl-1*H*-pyrrole-2-carboxylate (**33**)

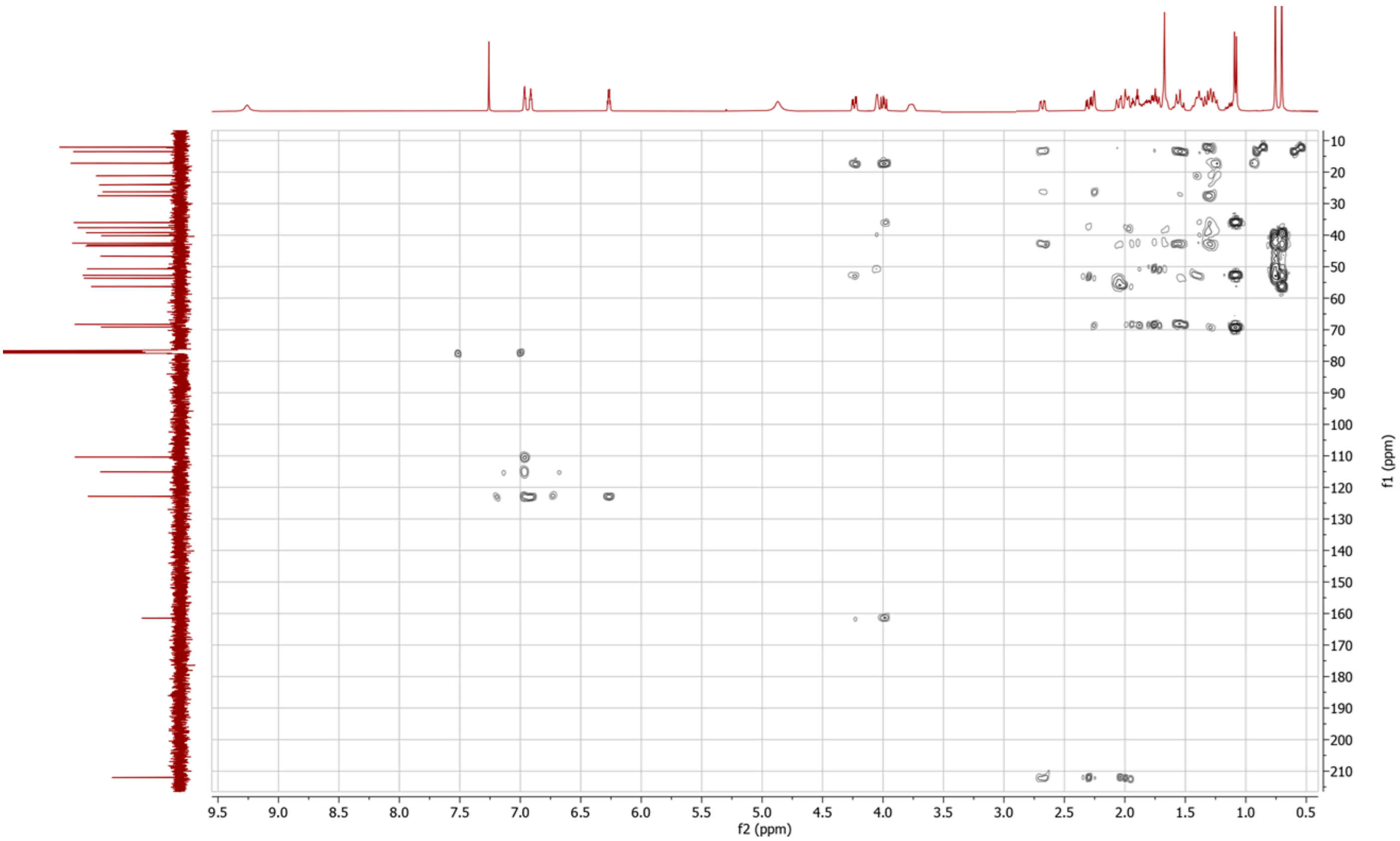

Figure S31. 2D ^{1}H-^{13}C HMBC spectrum of 2*α*,3*α*-Dihydroxy-6-oxo-23,24-dinor-5*α*-cholan-22-yl-1*H*-pyrrole-2-carboxylate (**33**)

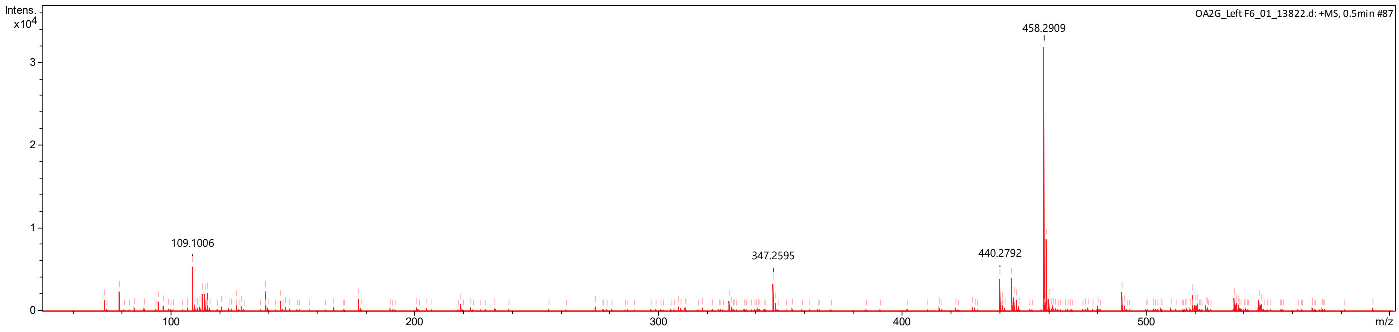

Figure S32. HRMS spectrum of 2*α*,3*α*-Dihydroxy-6-oxo-23,24-dinor-5*α*-cholan-22-yl-1*H*-pyrrole-2-carboxylate (**33**)

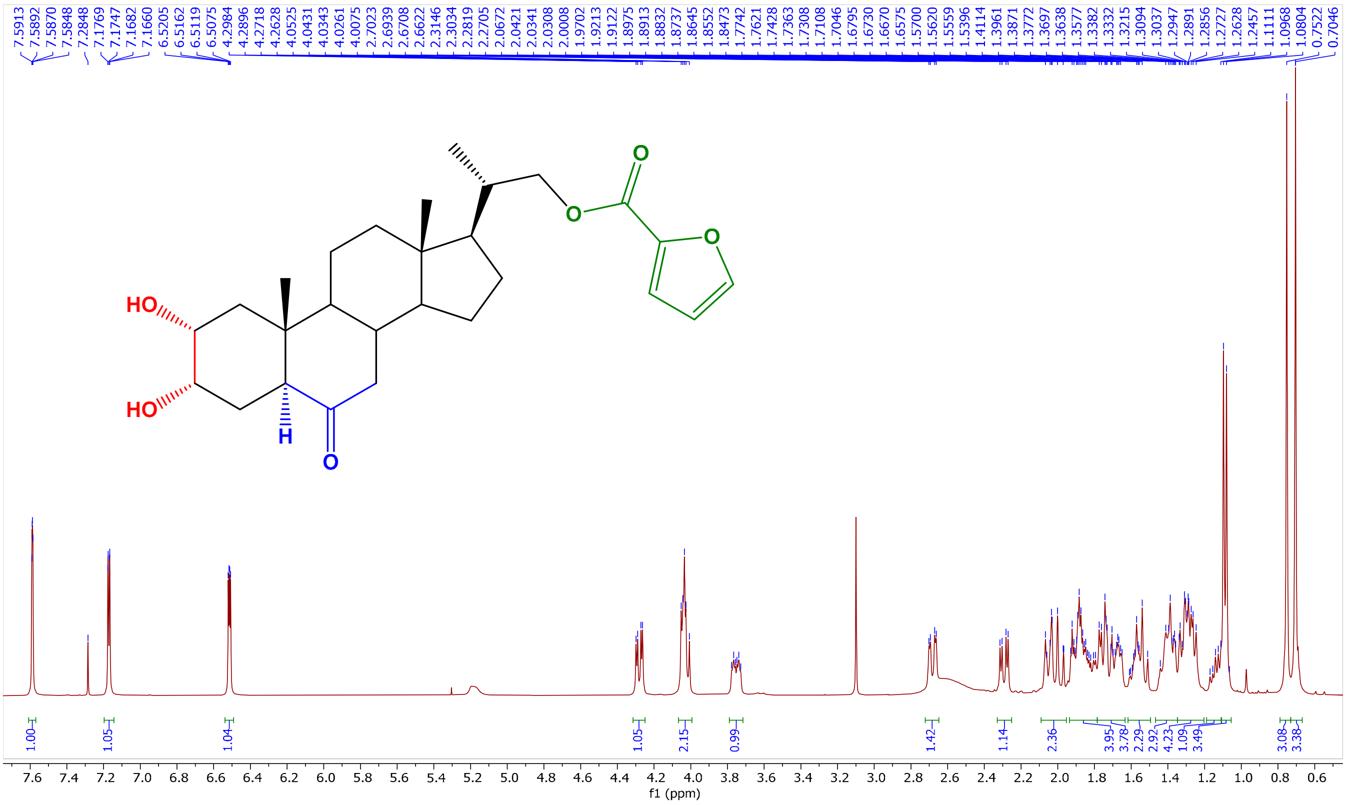

Figure S33. ^{1}H NMR spectrum of 2*α*,3*α*-Dihydroxy-6-oxo-23,24-dinor-5*α*-cholan-22-yl-furan-2-carboxylate (**34**)

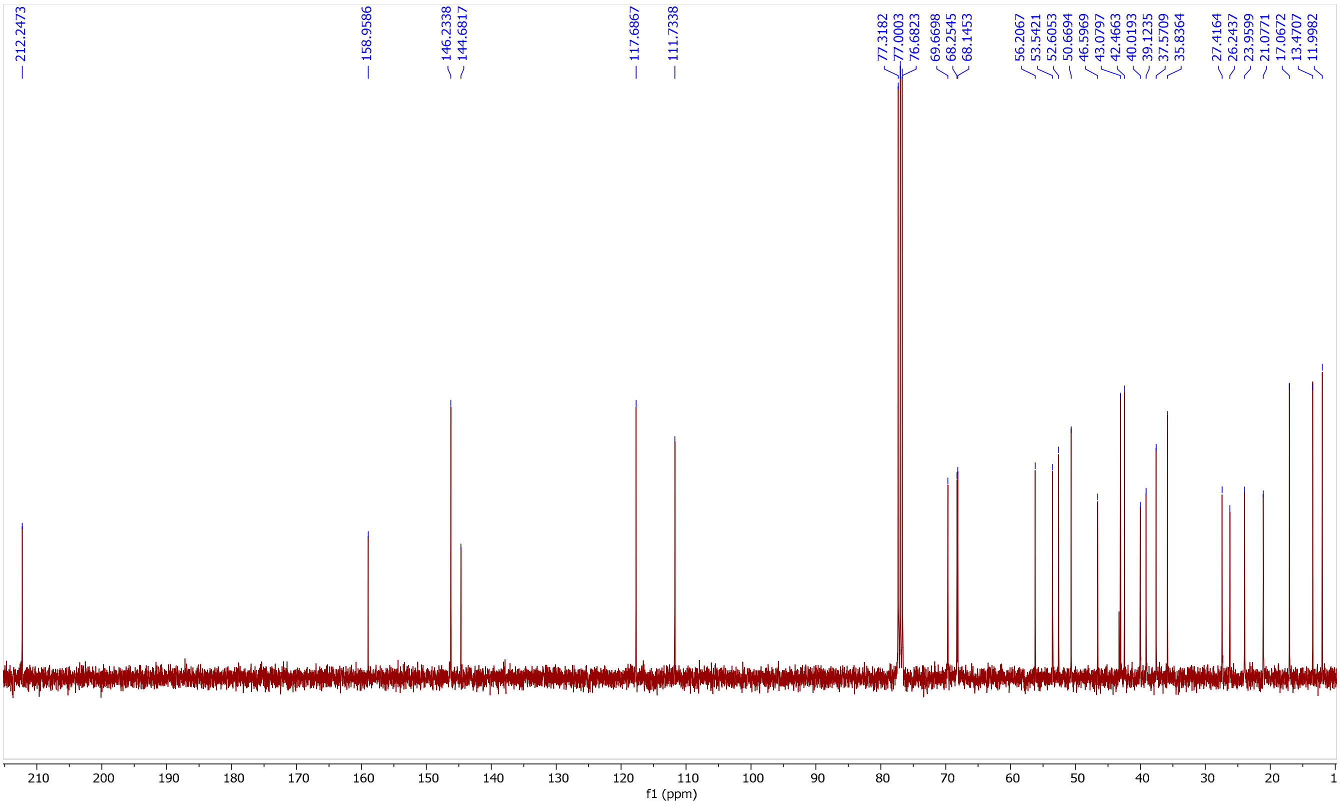

Figure S34. ^{13}C{^{1}H} NMR spectrum of 2*α*,3*α*-Dihydroxy-6-oxo-23,24-dinor-5*α*-cholan-22-yl-furan-2-carboxylate (**34**)

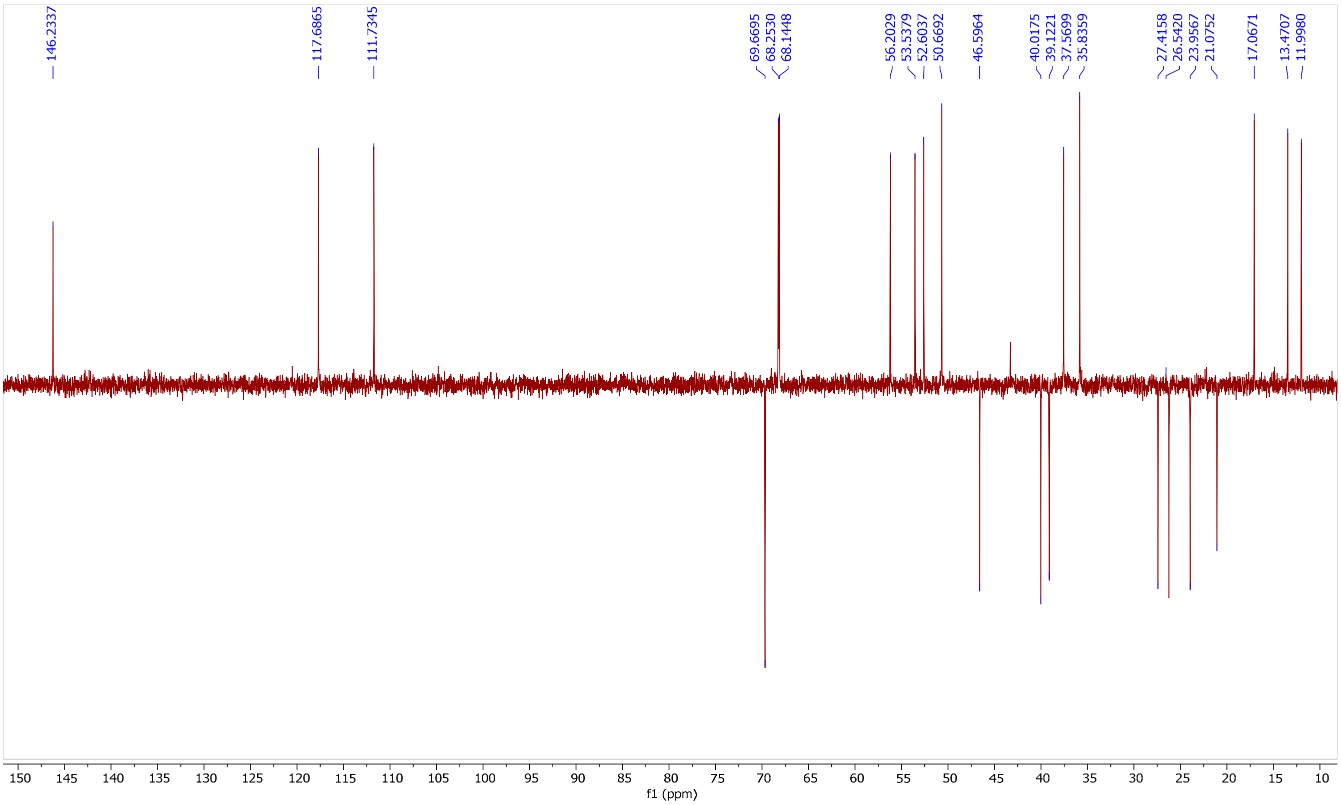

Figure S35. $^{13}C\{^{1}H\}$ DEPT-135 NMR spectrum of 2*α*,3*α*-Dihydroxy-6-oxo-23,24-dinor-5*α*-cholan-22-yl-furan-2-carboxylate (**34**)

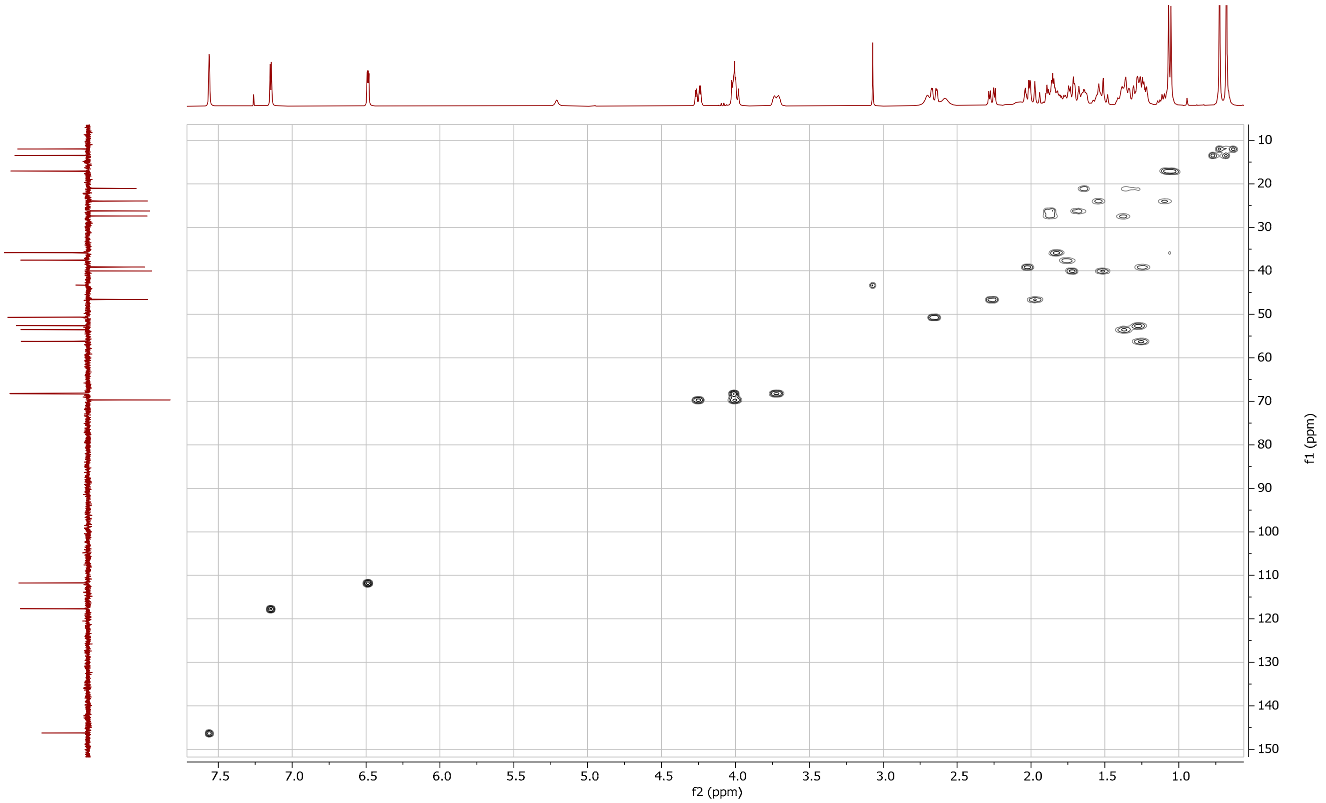

Figure S36. 2D ^{1}H-^{13}C HSQC spectrum of 2*α*,3*α*-Dihydroxy-6-oxo-23,24-dinor-5*α*-cholan-22-yl-furan-2-carboxylate (**34**)

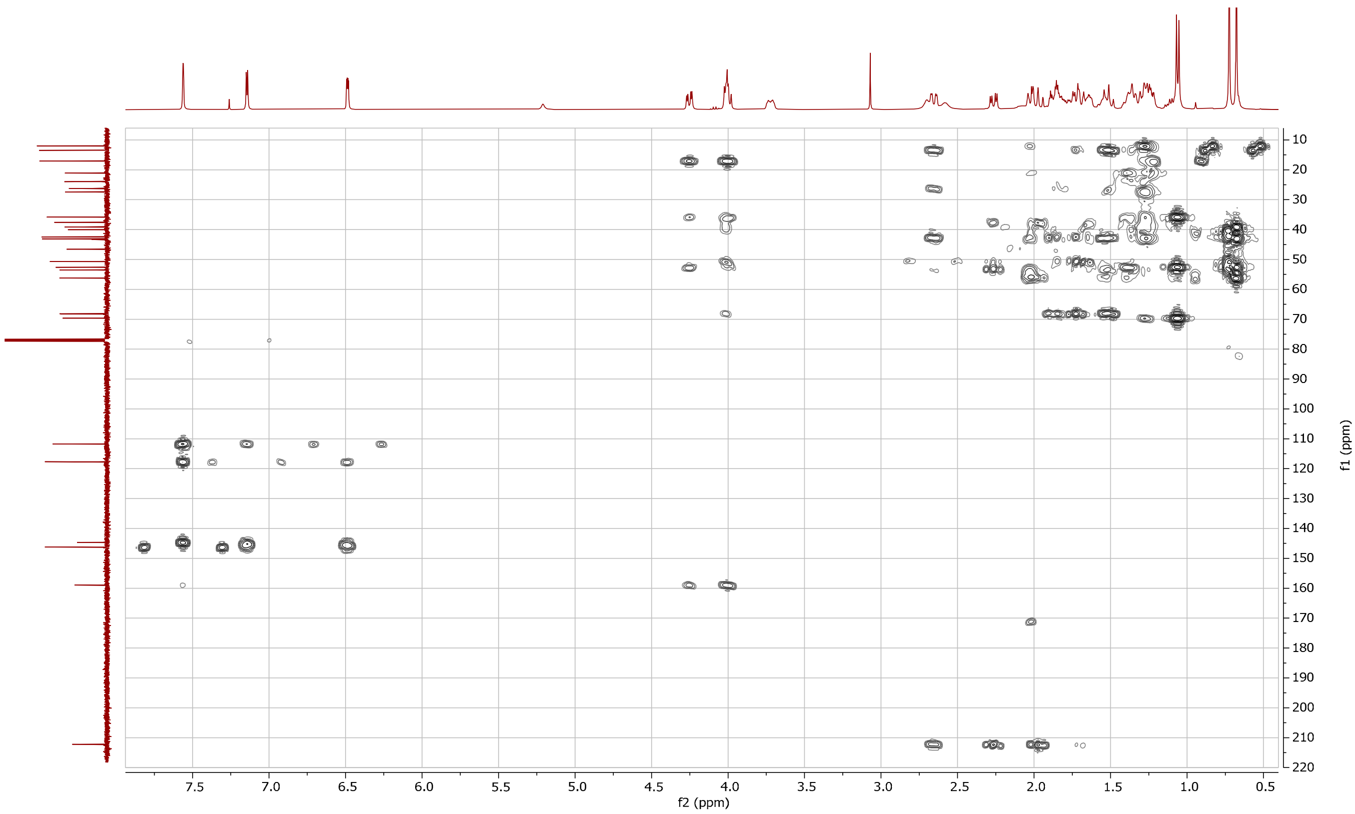

Figure 37. 2D ^{1}H-^{13}C HMBC spectrum of 2α,3α-Dihydroxy-6-oxo-23,24-dinor-5α-cholan-22-yl-furan-2-carboxylate (**34**)

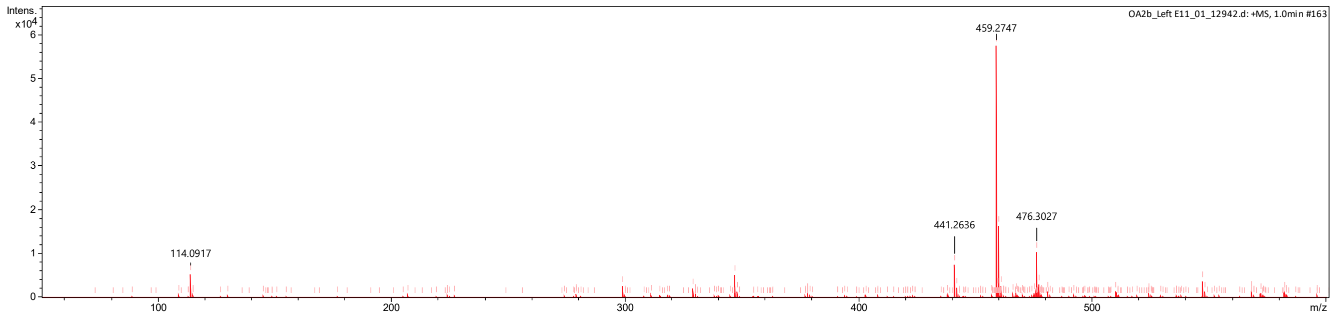

Figure S38. HRMS spectrum of 2α,3α-Dihydroxy-6-oxo-23,24-dinor-5α-cholan-22-yl-furan-2-carboxylate (**34**)

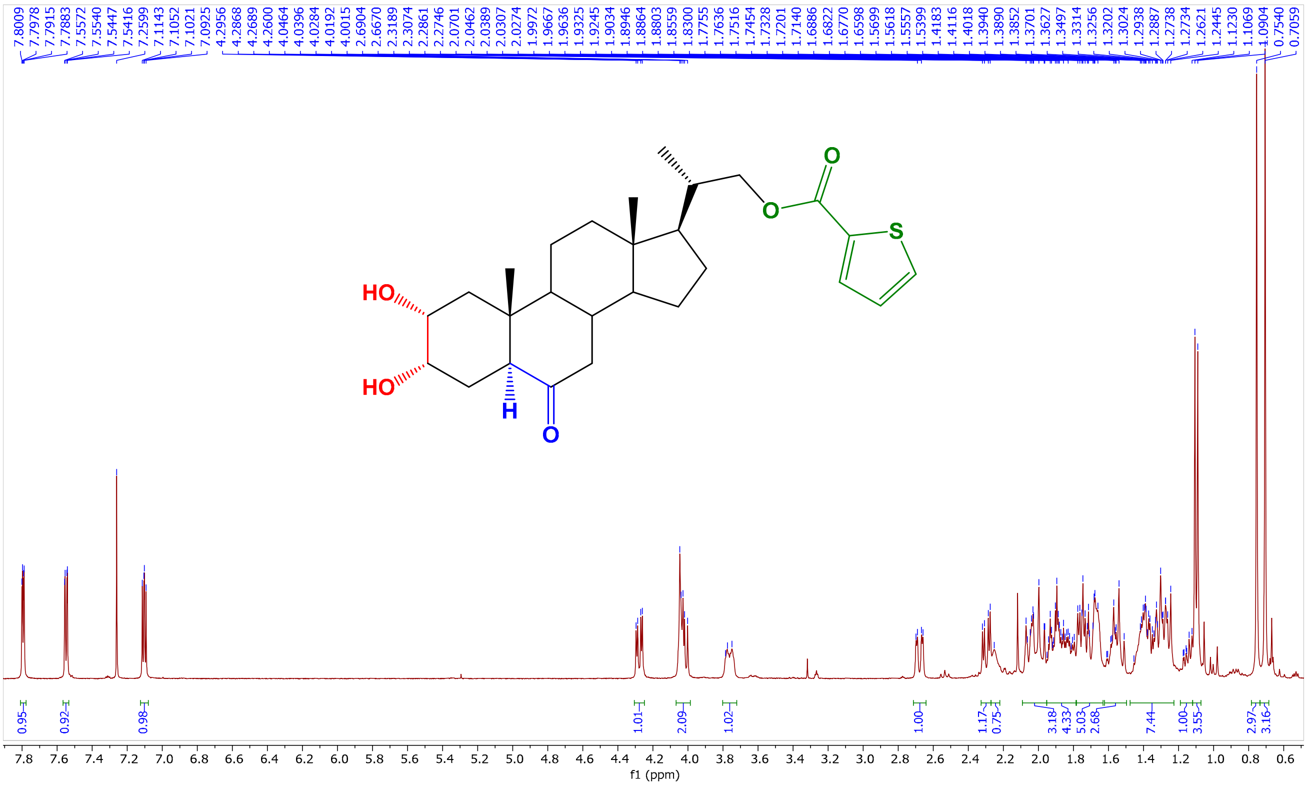

Figure S39. ^{1}H NMR spectrum of 2α,3α-Dihydroxy-6-oxo-23,24-dinor-5α-cholan-22-yl-thiophene-2-carboxylate (**35**)

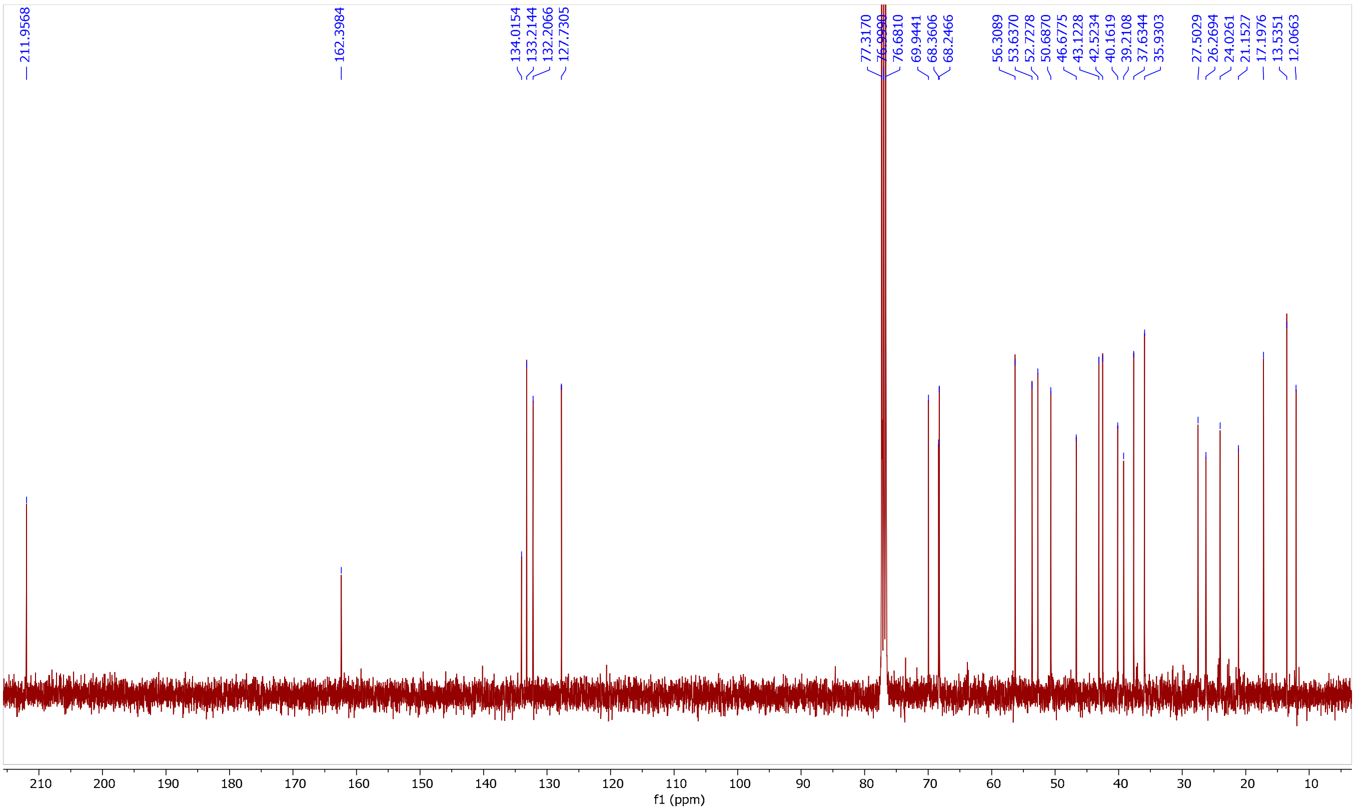

Figure S40. $^{13}C\{^{1}H\}$ NMR spectrum of 2α,3α-Dihydroxy-6-oxo-23,24-dinor-5α-cholan-22-yl-thiophene-2-carboxylate (**35**)

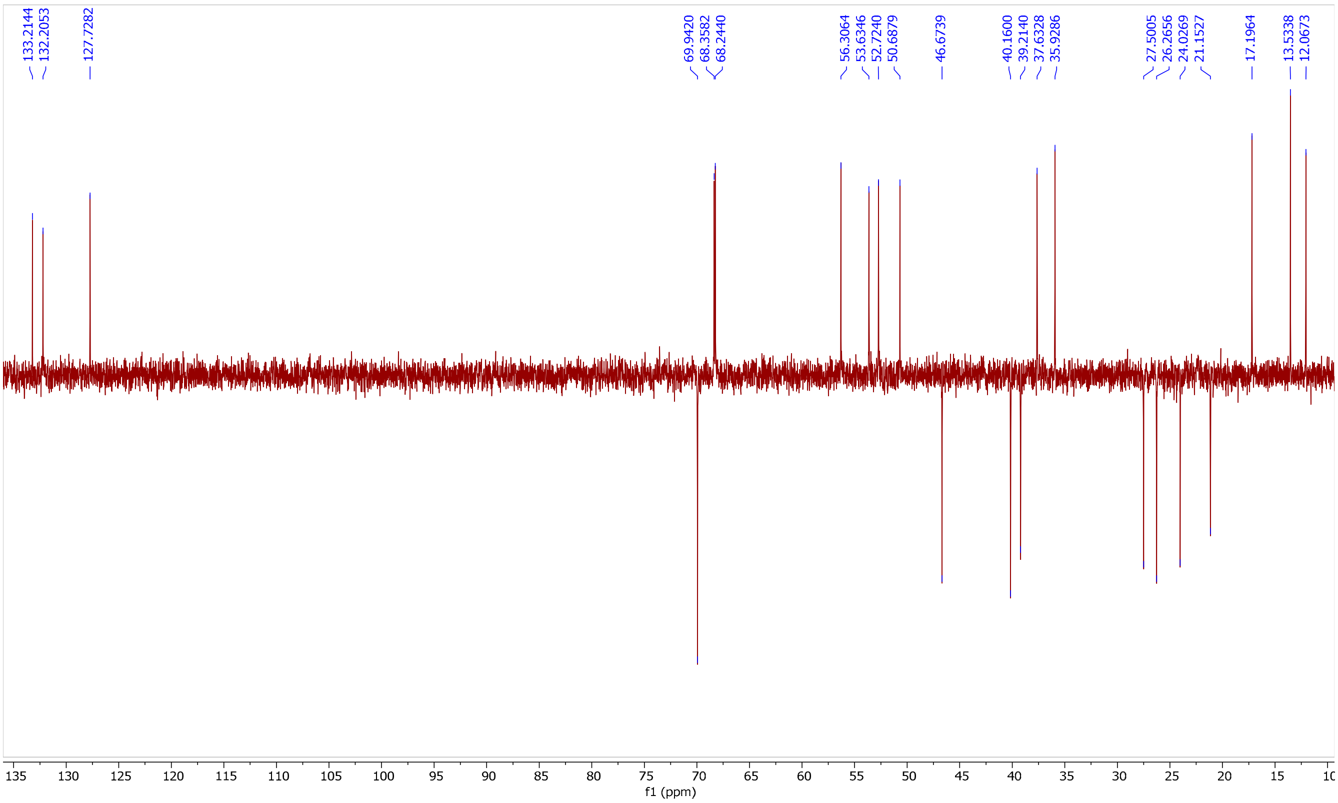

Figure S41. $^{13}C\{^{1}H\}$ DEPT-135 NMR spectrum of 2α,3α-Dihydroxy-6-oxo-23,24-dinor-5α-cholan-22-yl-thiophene-2-carboxylate (**35**)

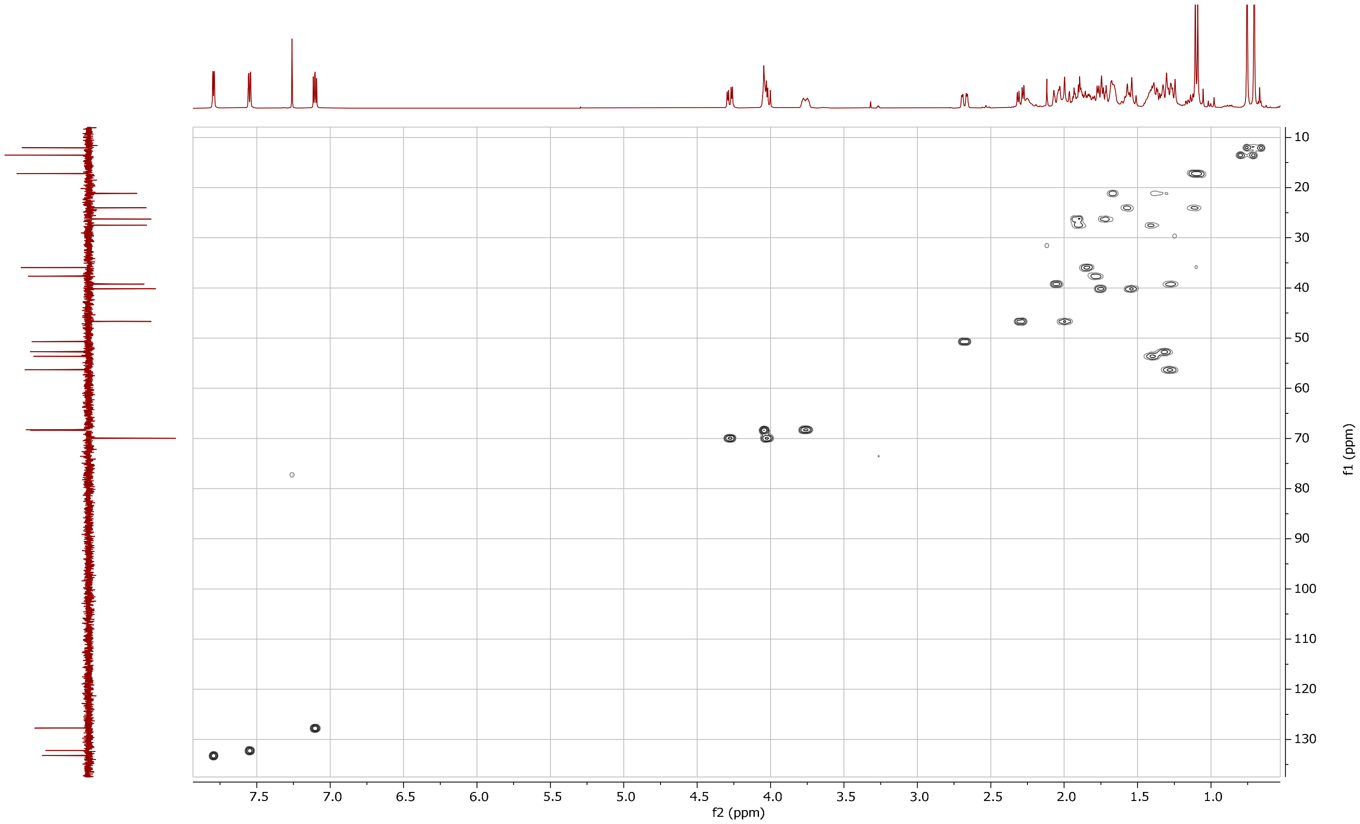

Figure S42. 2D ^{1}H-^{13}C HSQC spectrum of 2α,3α-Dihydroxy-6-oxo-23,24-dinor-5α-cholan-22-yl-thiophene-2-carboxylate (**35**)

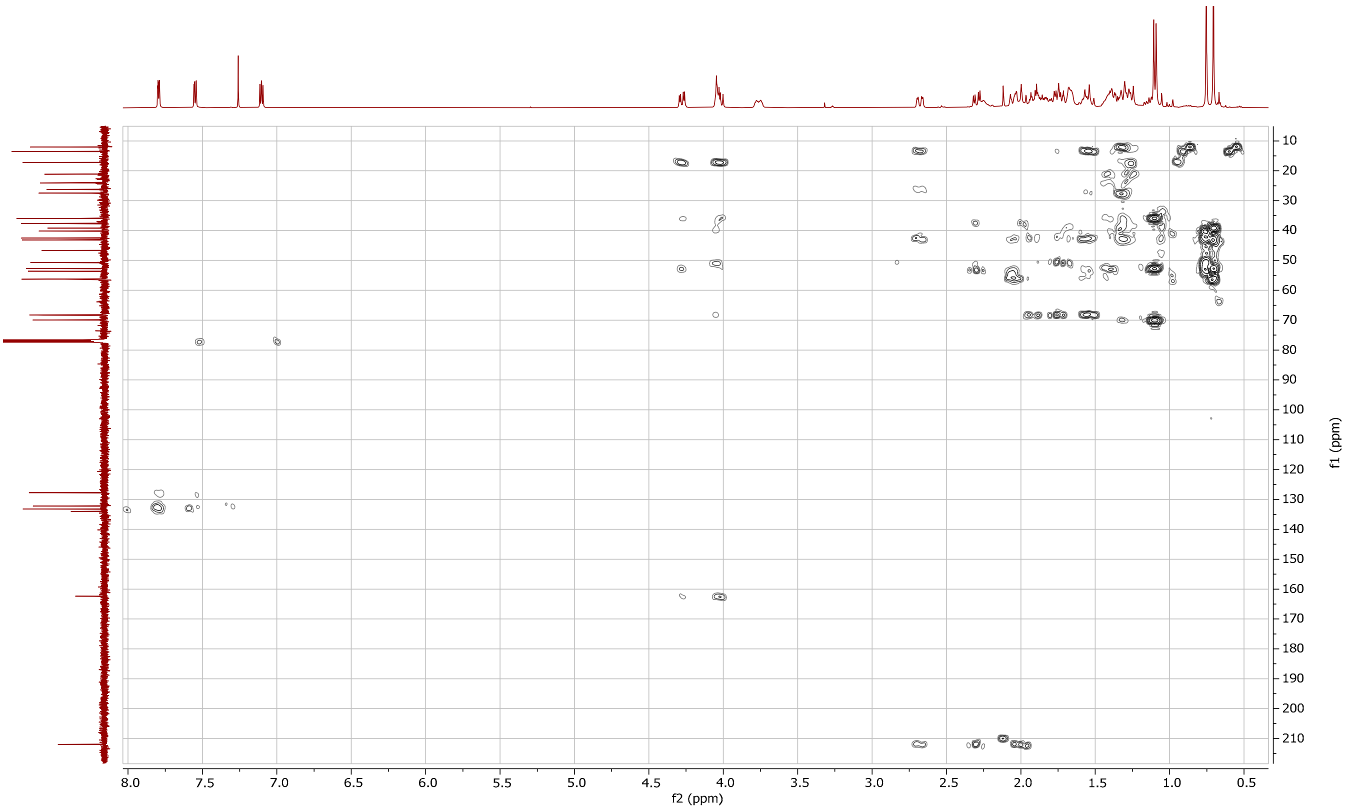

Figure S43. 2D ^{1}H-^{13}C HMBC spectrum of 2α,3α-Dihydroxy-6-oxo-23,24-dinor-5α-cholan-22-yl-thiophene-2-carboxylate (**35**)

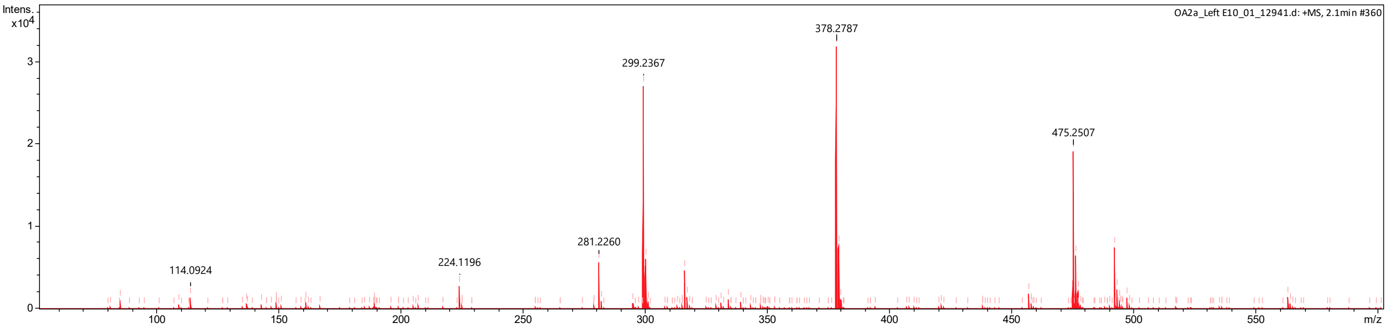

Figure S44. HRMS spectrum of 2α,3α-Dihydroxy-6-oxo-23,24-dinor-5α-cholan-22-yl-thiophene-2-carboxylate (**35**)

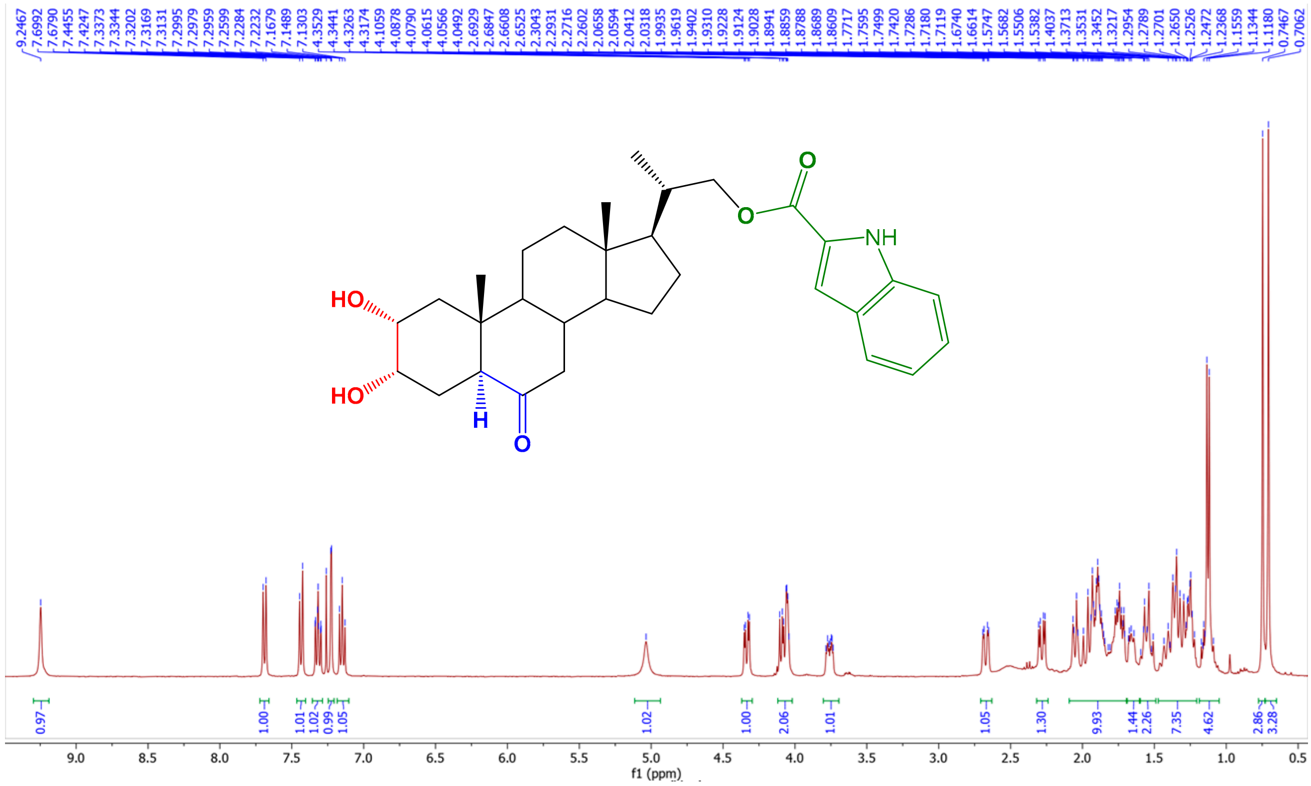

Figure S45. ^{1}H NMR spectrum of 2*α*,3*α*-Dihydroxy-6-oxo-23,24-dinor-5*α*-cholan-22-yl-1*H*-indole-2-carboxylate (**36**)

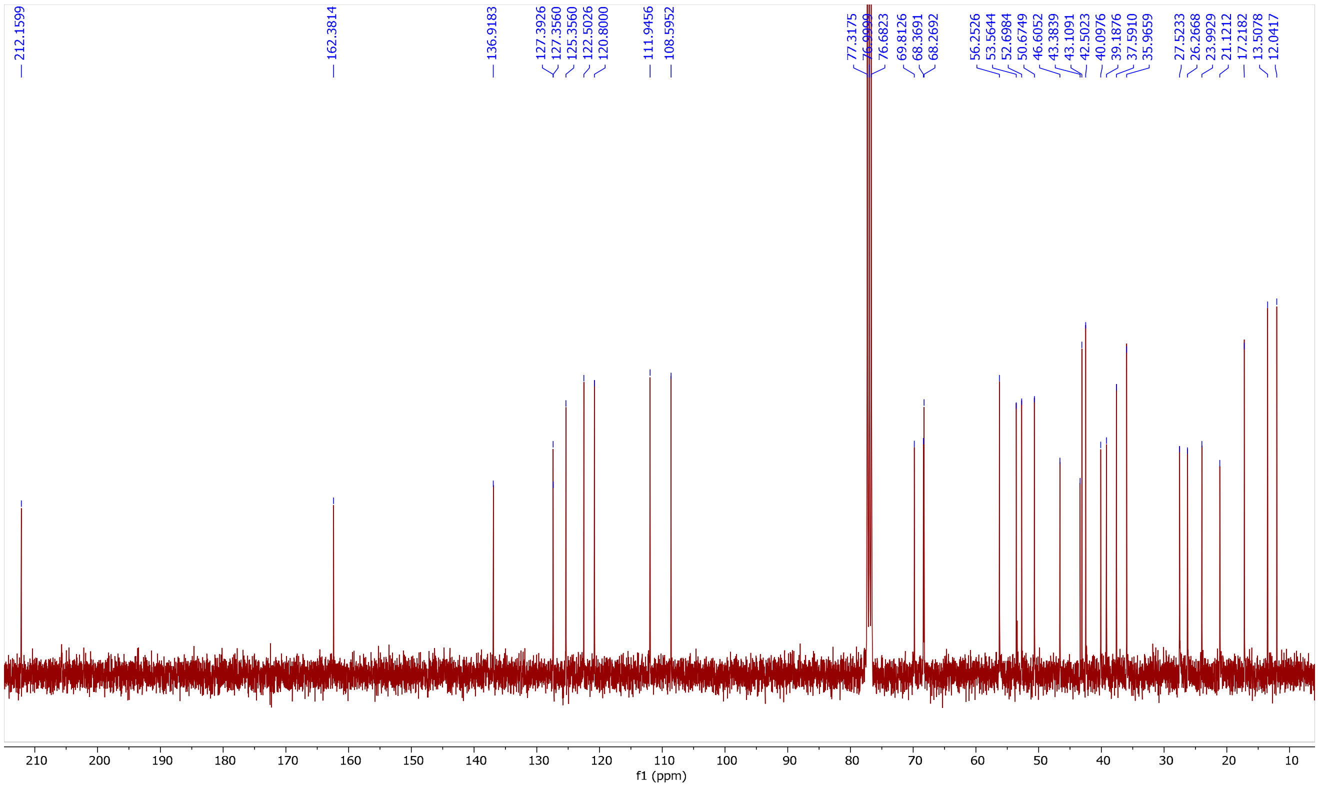

Figure S46. ^{13}C{^{1}H} NMR spectrum of 2*α*,3*α*-Dihydroxy-6-oxo-23,24-dinor-5*α*-cholan-22-yl-1*H*-indole-2-carboxylate (**36**)

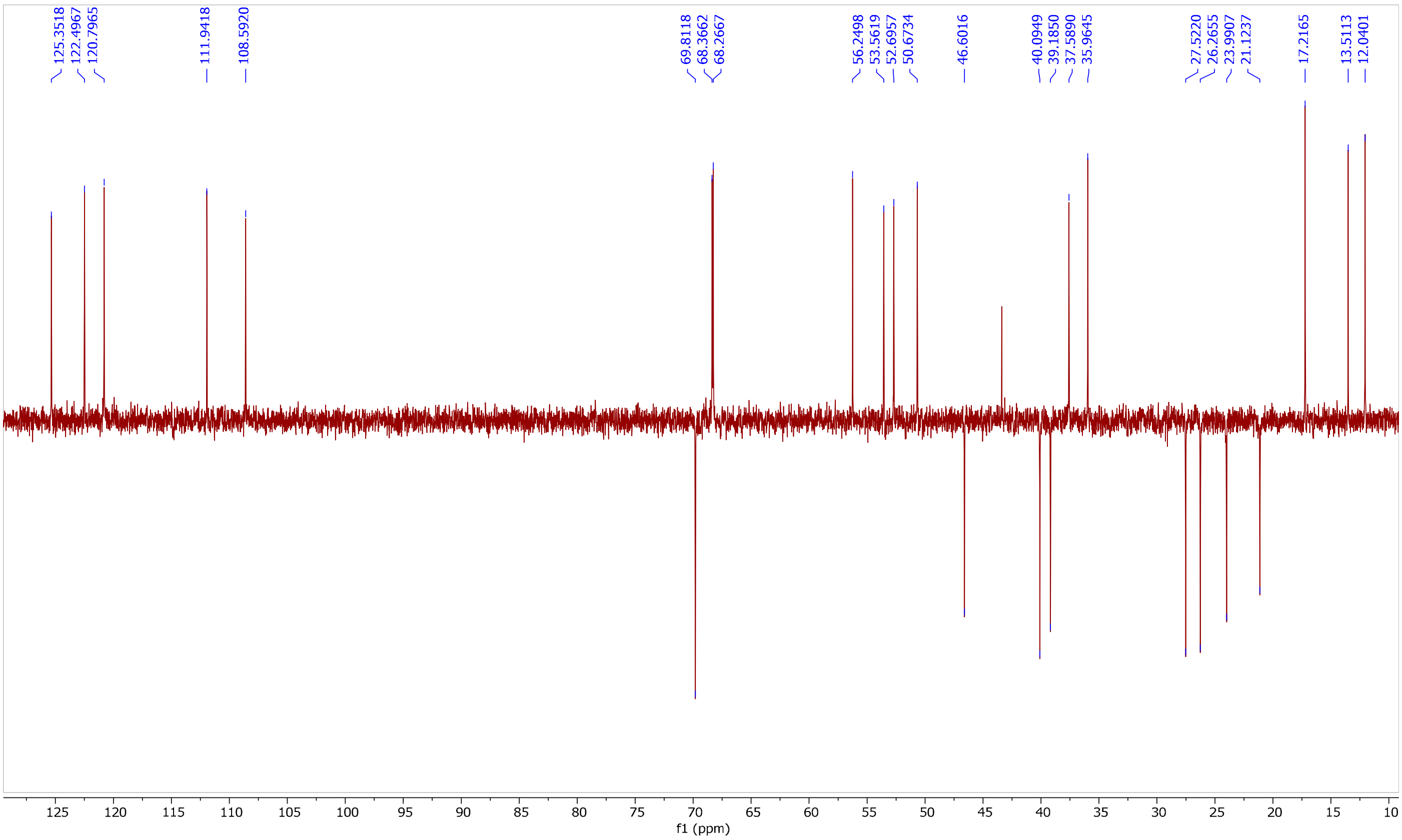

Figure S47. $^{13}C\{^{1}H\}$ DEPT-135 NMR spectrum of 2*α*,3*α*-Dihydroxy-6-oxo-23,24-dinor-5*α*-cholan-22-yl-1*H*-indole-2-carboxylate (**36**)

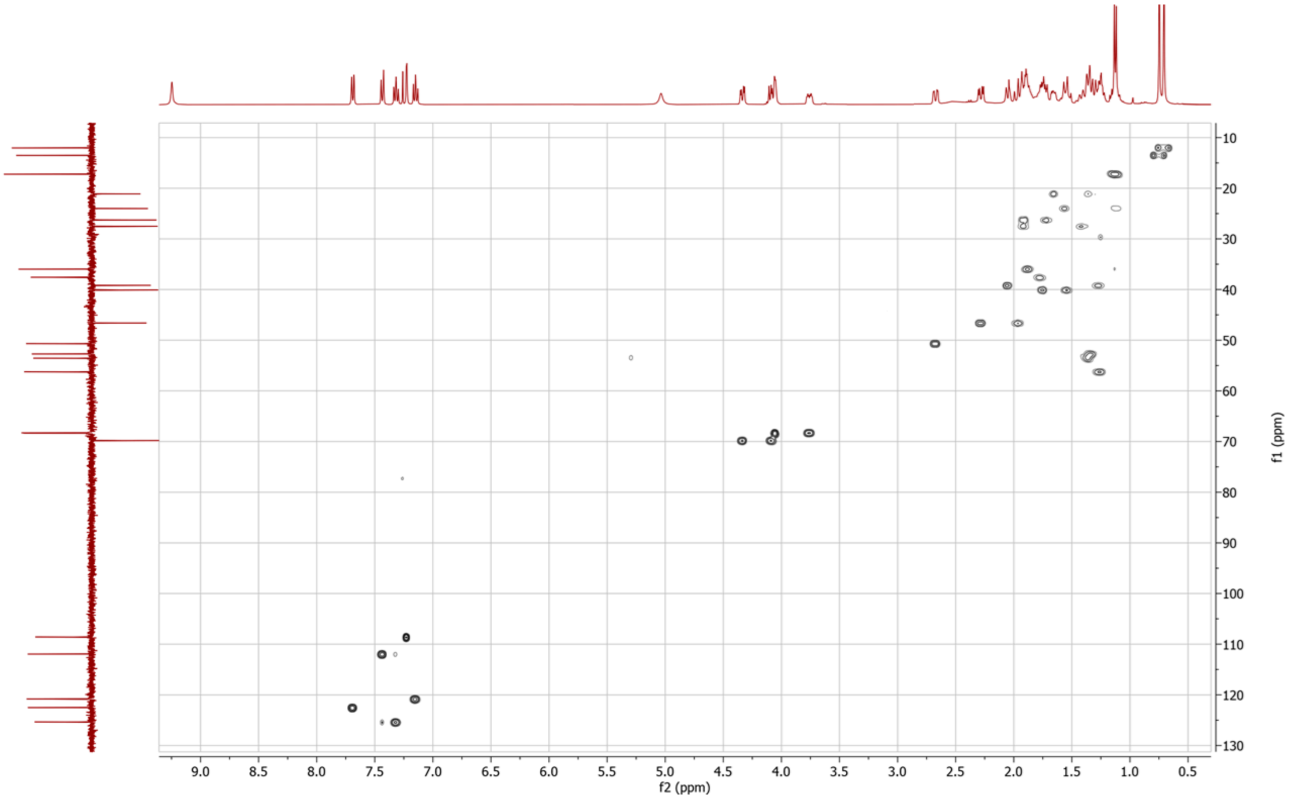

Figure S48. 2D ^{1}H-^{13}C HSQC spectrum of 2*α*,3*α*-Dihydroxy-6-oxo-23,24-dinor-5*α*-cholan-22-yl-1*H*-indole-2-carboxylate (**36**)

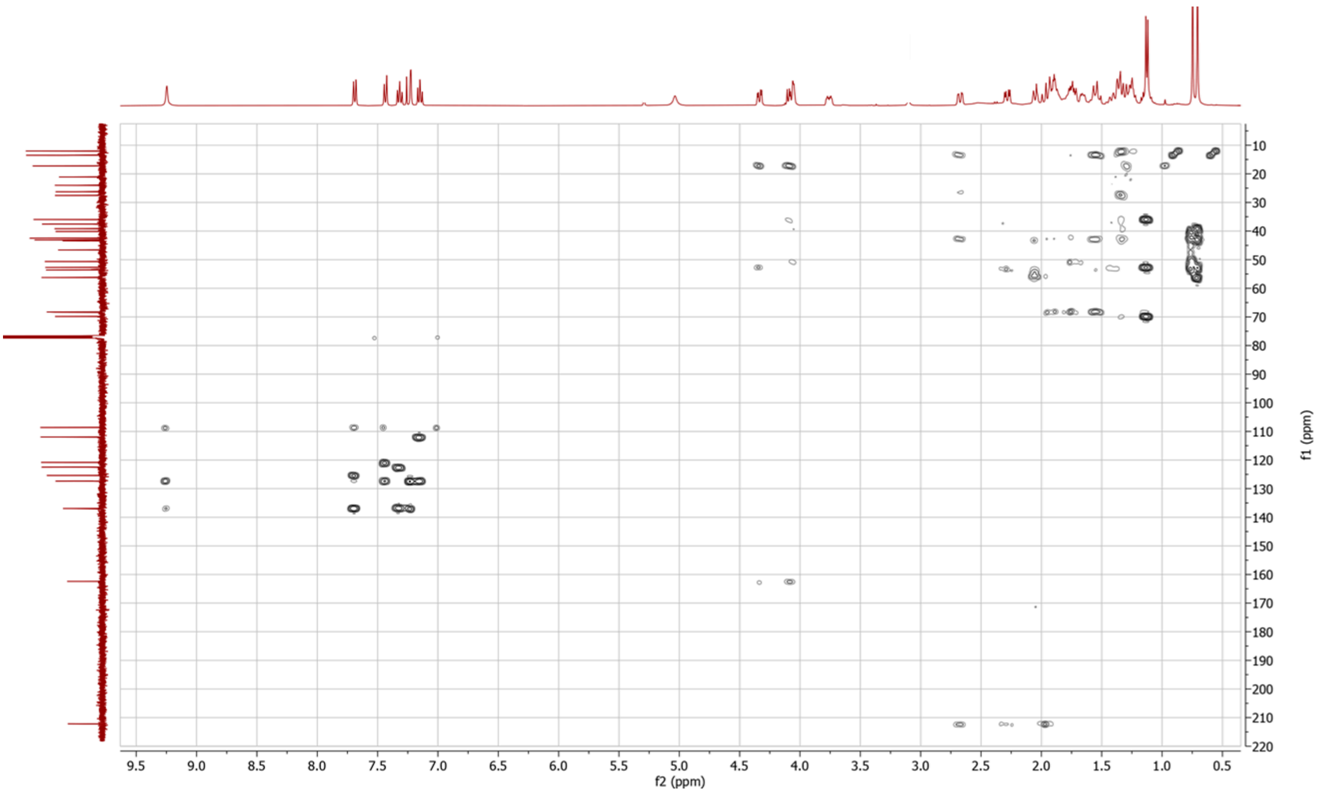

Figure S49. 2D ^{1}H-^{13}C HMBC spectrum of 2*α*,3*α*-Dihydroxy-6-oxo-23,24-dinor-5*α*-cholan-22-yl-1*H*-indole-2-carboxylate (**36**)

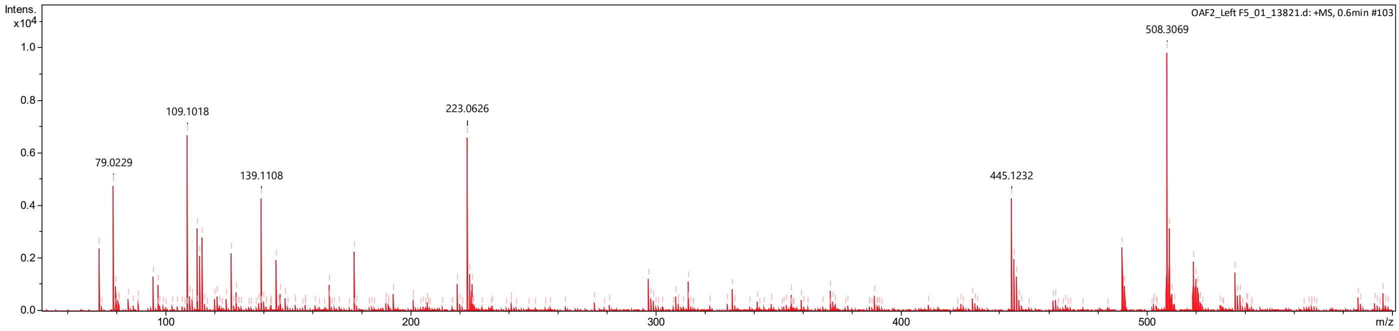

Figure S50. HRMS spectrum of 2*α*,3*α*-Dihydroxy-6-oxo-23,24-dinor-5*α*-cholan-22-yl-1*H*-indole-2-carboxylate (**36**)

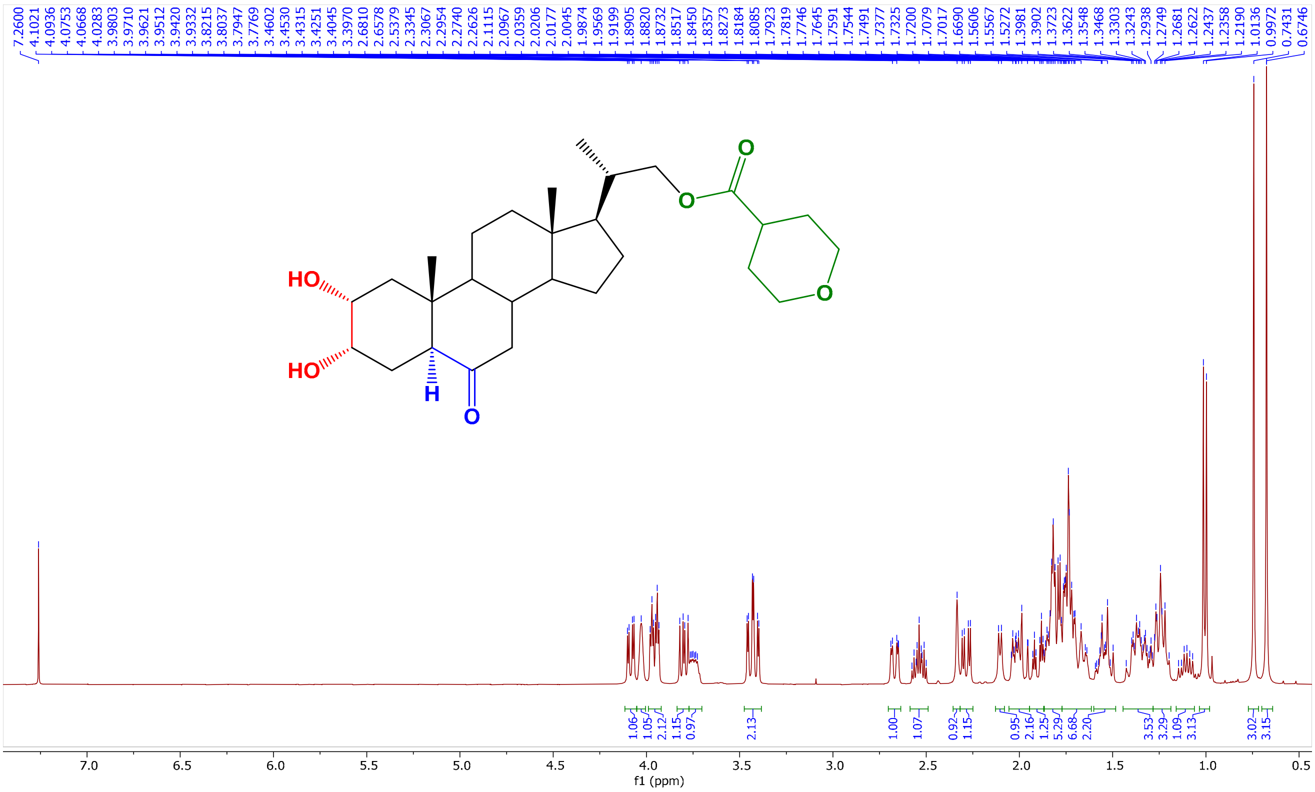

Figure S51. ^{1}H NMR spectrum of 2*α*,3*α*-Dihydroxy-6-oxo-23,24-dinor-5*α*-cholan-22-yl-2*H*-pyran-4-carbocarboxylate (**37**)

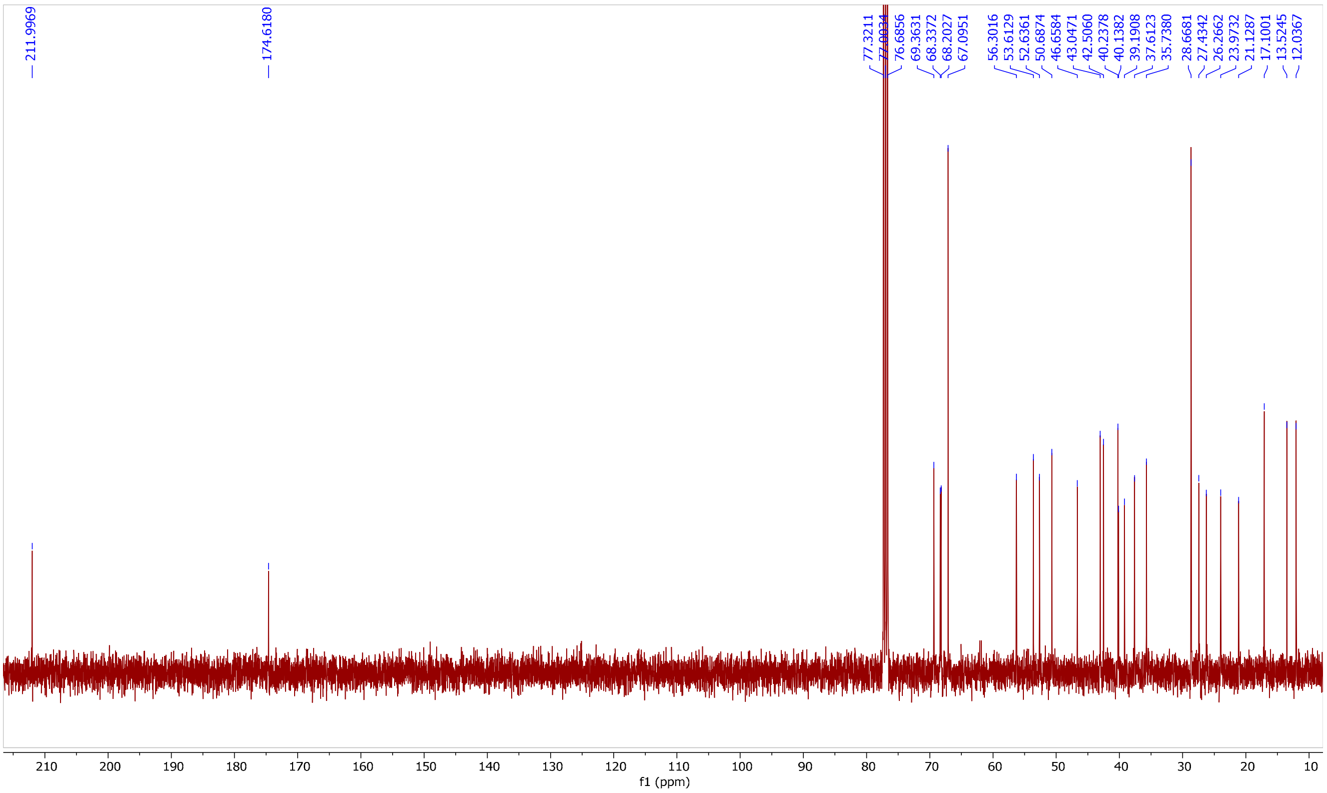

Figure S52. ^{13}C{^{1}H} NMR spectrum of 2*α*,3*α*-Dihydroxy-6-oxo-23,24-dinor-5*α*-cholan-22-yl-2*H*-pyran-4-carbocarboxylate (**37**)

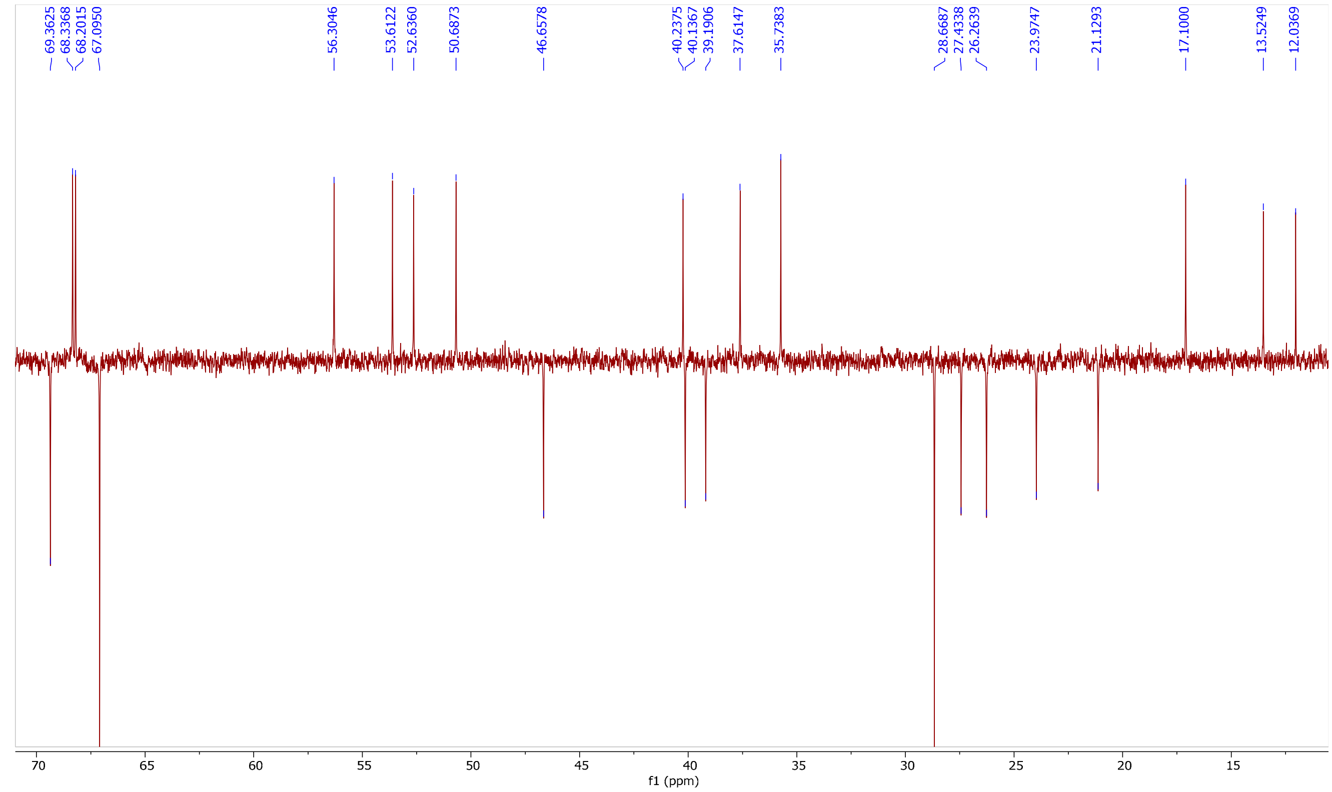

Figure S53. $^{13}C\{^{1}H\}$ DEPT-135 NMR spectrum of 2*α*,3*α*-Dihydroxy-6-oxo-23,24-dinor-5*α*-cholan-22-yl-2*H*-pyran-4-carbocarboxylate (**37**)

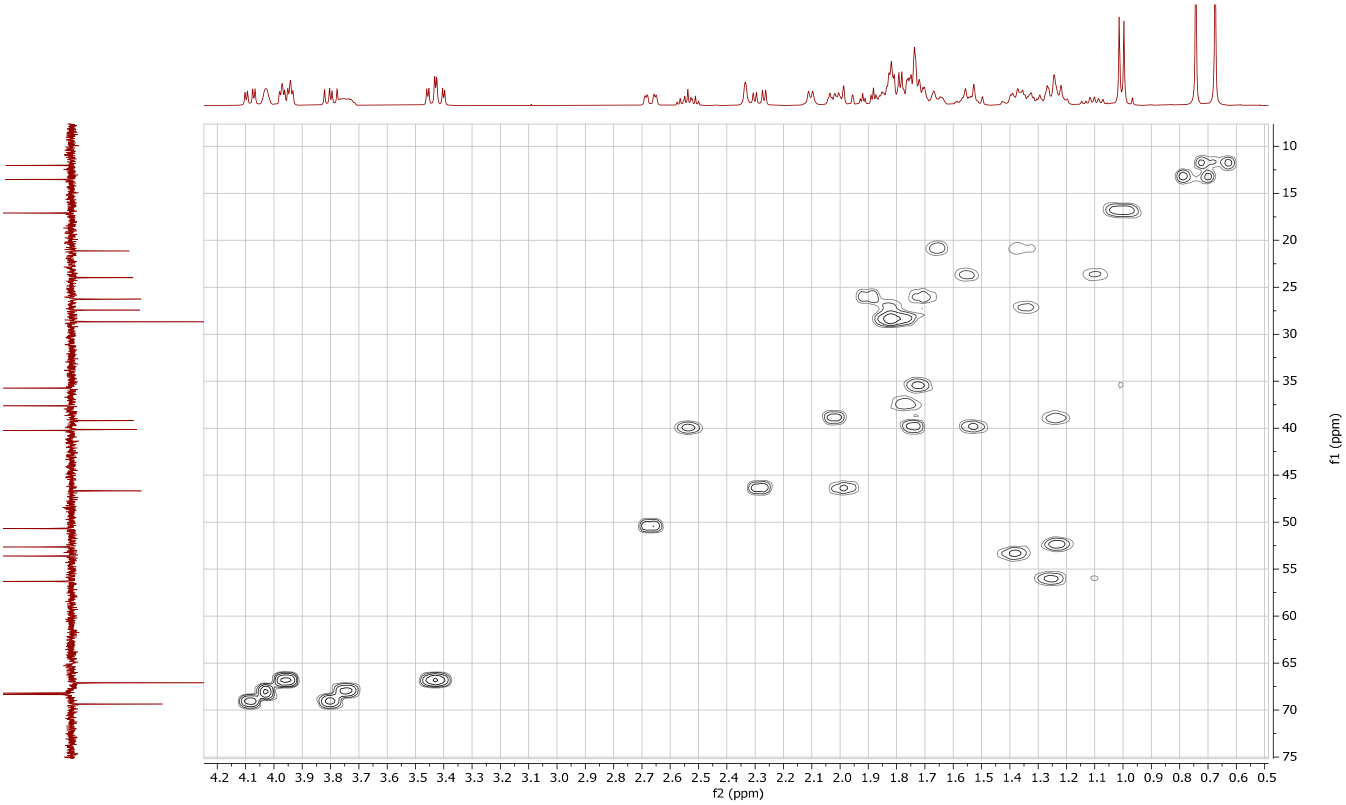

Figure S54. 2D ^{1}H-^{13}C HSQC spectrum of 2*α*,3*α*-Dihydroxy-6-oxo-23,24-dinor-5*α*-cholan-22-yl-2*H*-pyran-4-carbocarboxylate (**37**)

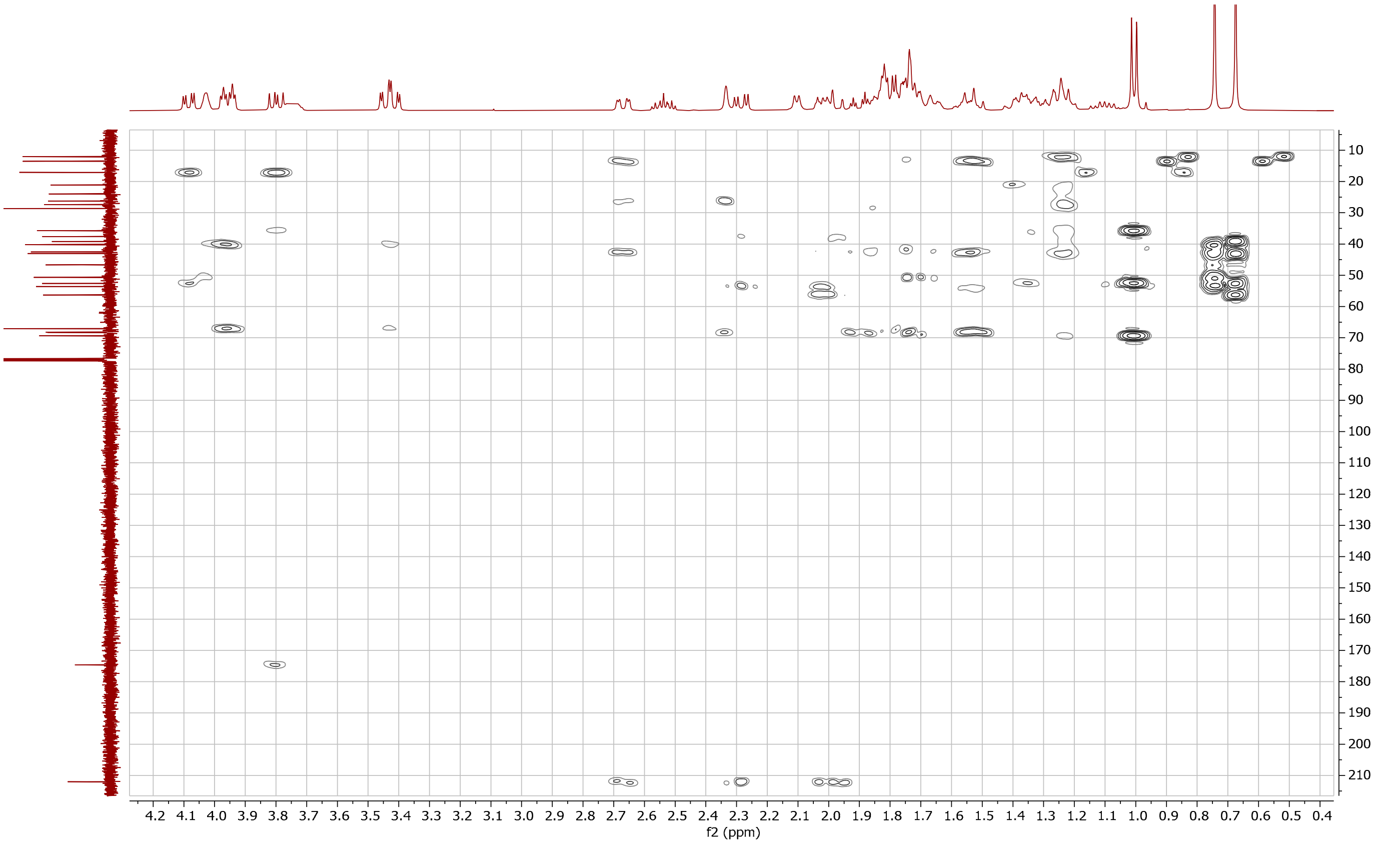

220

Figure S55. 2D ¹H-¹³C HMBC spectrum of 2*α*,3*α*-Dihydroxy-6-oxo-23,24-dinor-5*α*-cholan-22-yl-2*H*-pyran-4-carbocar- 221
boxylate (**37**) 222

223

224

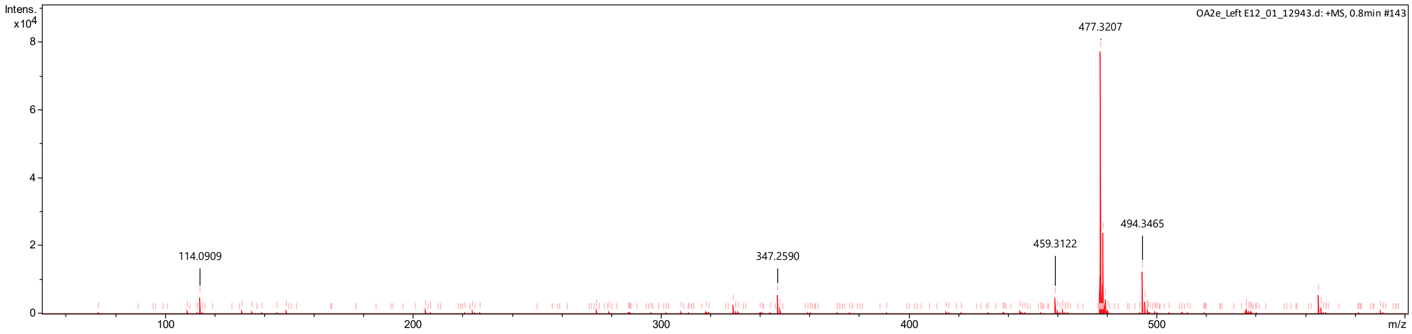

225

Figure S56. HRMS spectrum of 2*α*,3*α*-Dihydroxy-6-oxo-23,24-dinor-5*α*-cholan-22-yl-2*H*-pyran-4-carbocarboxylate (**37**) 226

227

228

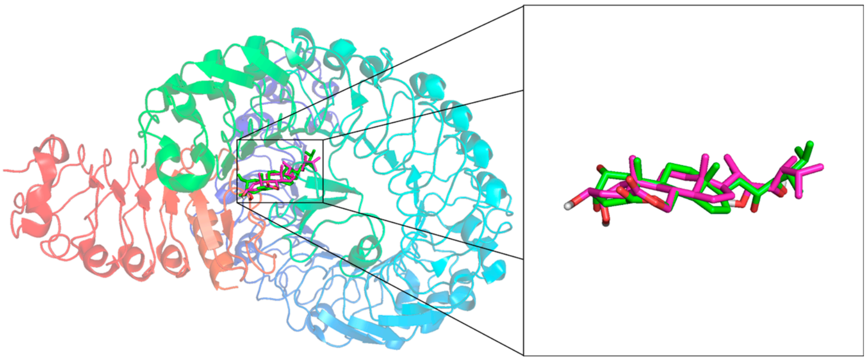

Figure S57. Redocking of brassinolide into the active site of the BRI1–BAK1. BRI1–BAK1 in cartoon mode and ligands in stick mode. Ligand color code: Green, C atoms of crystallyzed ligand BLB808; Magent: C atoms of ligand docked by using Autodock vina; Red: oxygen atoms.

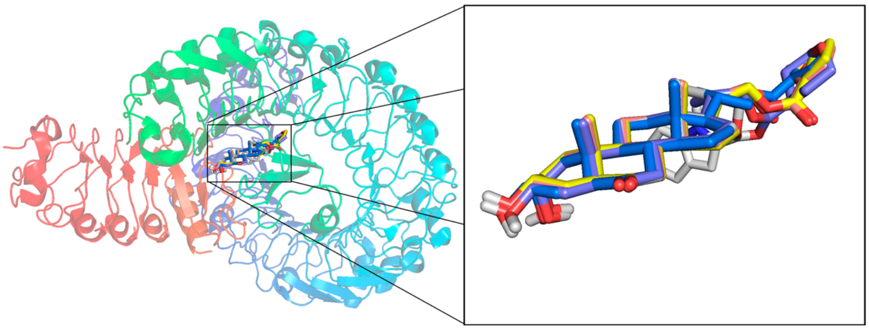

Figure S58. Results of molecular docking for new BRs analogs. BRI1–BAK1 in cartoon mode and ligands in stick mode. Ligand color code for carbon atoms: Blue: compound **33**; Yellow: compoundo **34**; Orange: compound **35**; White: compound **36**; Violet: compound **37**. Oxygen atoms: Red.

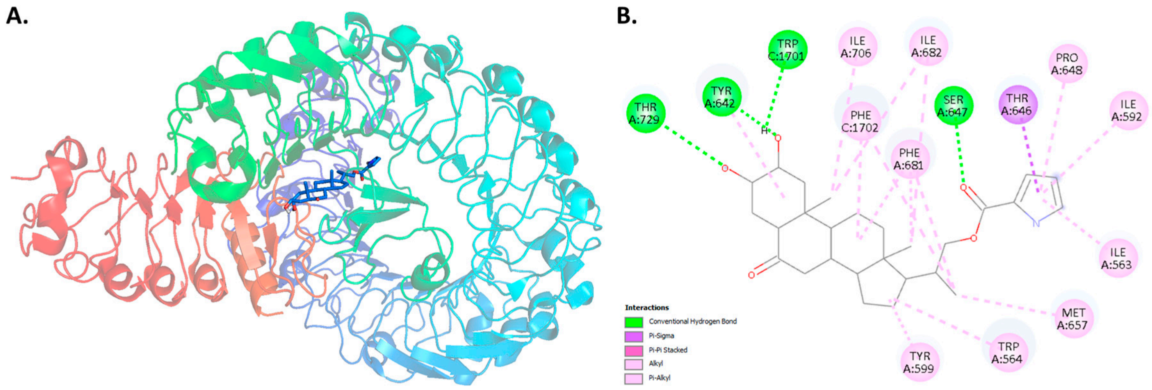

Figure S59. Results of molecular docking of compound **33** in the active site of BRI1–BAK1. A. 3D visualization and B. 2D representations of the most important interactions.

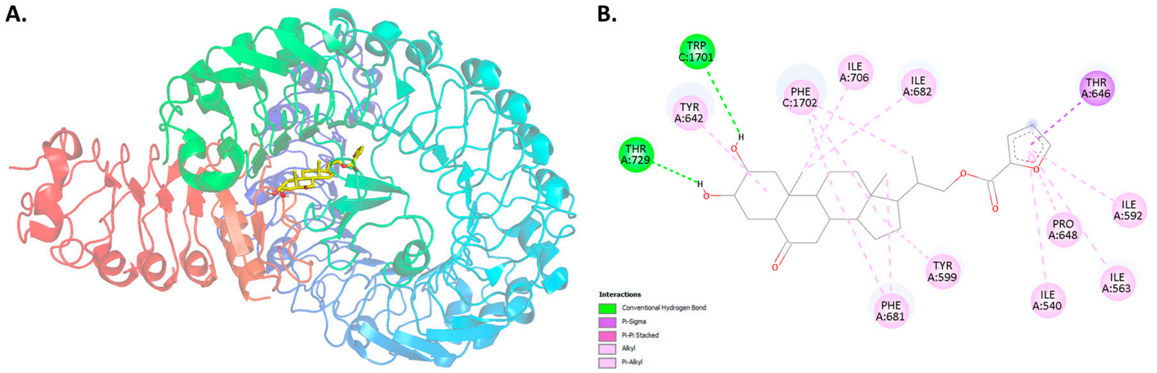

Figure S60. Results of molecular docking of compound **34** in the active site of BRI1–BAK1. A. 3D visualization and B. 2D representations of the most important interactions.

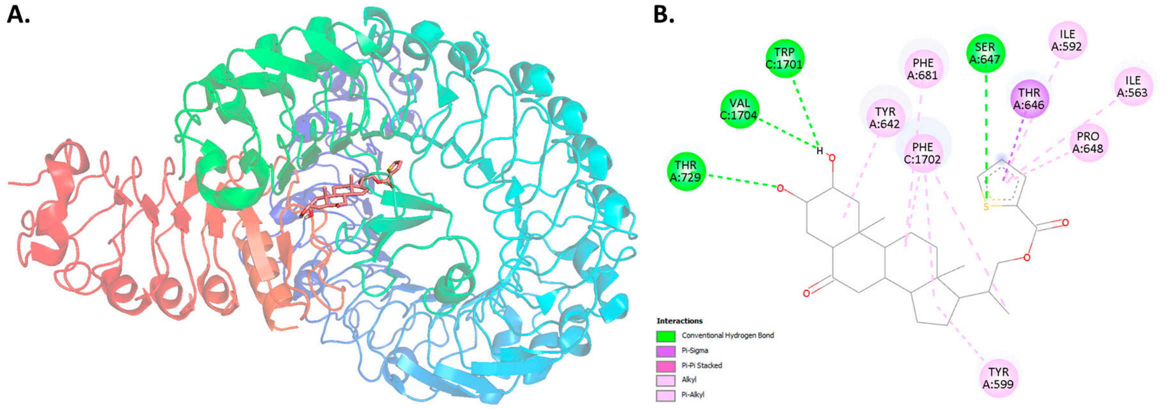

Figure S61. Results of molecular docking of compound **35** in the active site of BRI1–BAK1. A. 3D visualization and B. 2D representations of the most important interactions.

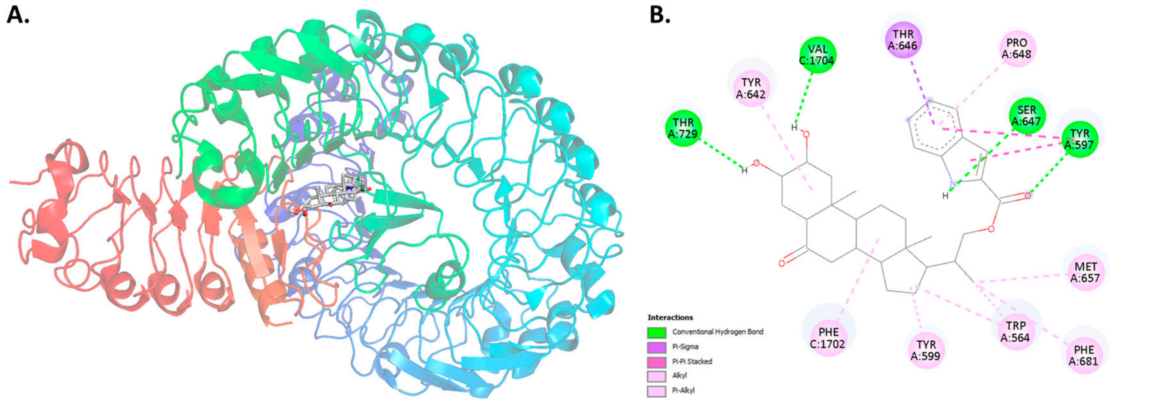

Figure S62. Results of molecular docking of compound **36** in the active site of BRI1–BAK1. A. 3D visualization and B. 2D representations of the most important interactions.

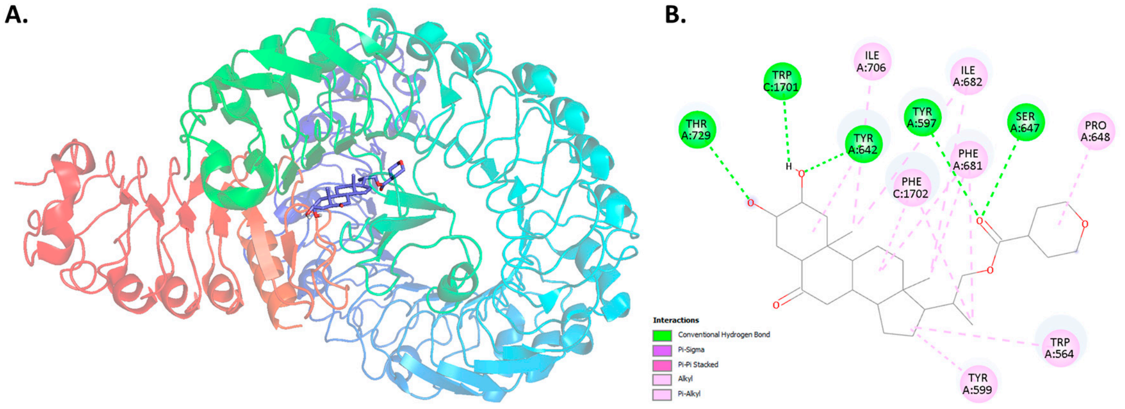

Figure S63. Results of molecular docking of compound **37** in the active site of BRI1–BAK1. A. 3D visualization and B. 2D representations of the most important interactions.

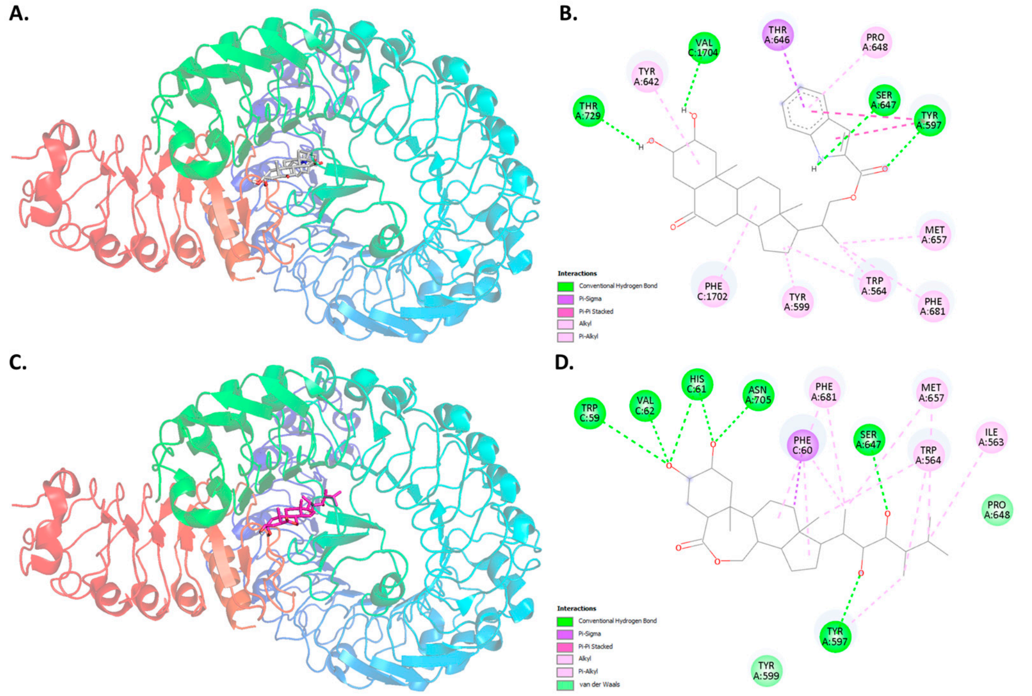

Figure S64. Results of molecular docking for compound **36** and brassinolide within the active site of BRI1–BAK1. A. 3D visualization of compound **36**; B. 2D representation of interactions of **36** with residues in BRI1–BAK1; C. 3D visualization of brassinolide docked to the active site; D. 2D representation of interactions of brassinolide with residues in BRI1–BAK1.

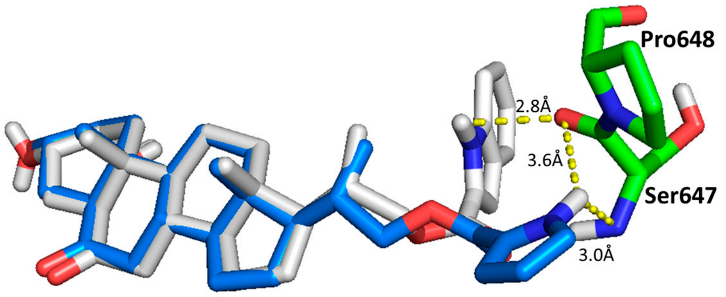

Figure S65: Molecular docking results for **33** and **36** in the active site of BRI1–BAK1, showing the aminoacids and distance for potential hydrogen bonding. Color code: Blue: C atoms of **33**; White: C atoms compound **33**; Green: C atomos of BRI1–BAK1.

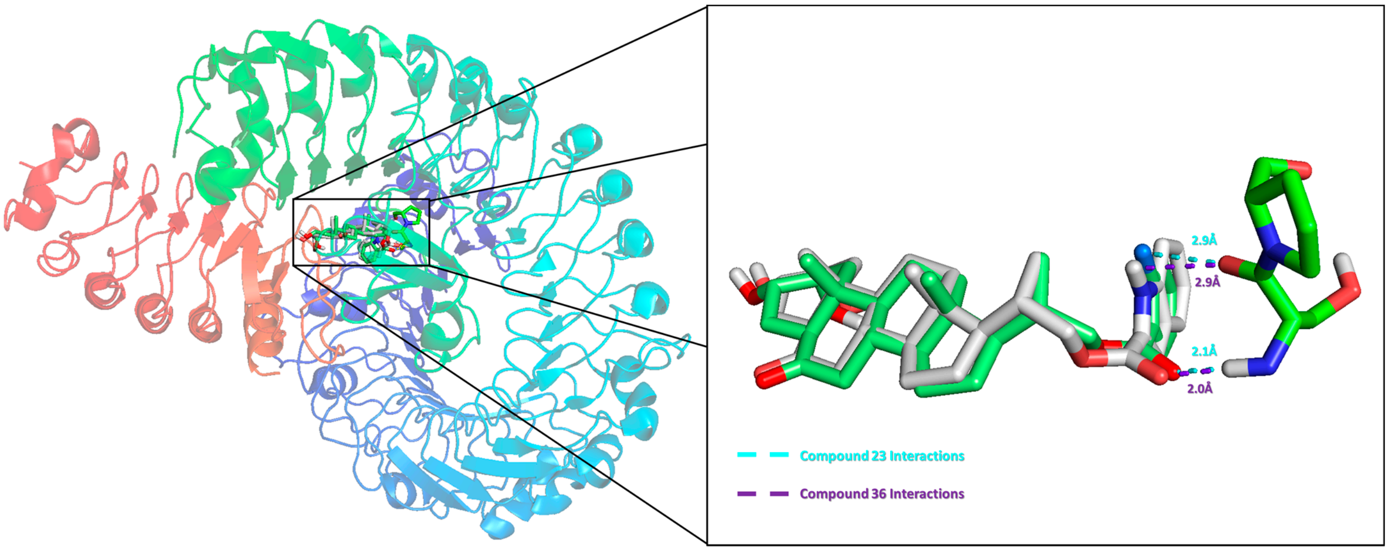

Figure S66. Results of molecular docking of compound **36** and compound **23** in the active site of BRI1–BAK1 showing the aminoacids and distance for potential hydrogen bonding. **Color code:** White: C atoms of **36**; Lime: C atoms compound **23**; Navy: F atom of **23**. Green: C atoms of BRI1–BAK1.

Table S1. Binding energies and interactions obtained for BRs analogs (**33-37**) and brassinolide

Compound	Binding energy (kcal/mol)	Interactions
**Brassinolide**	-12.9	**Hydrogen bond:**His61(3.2 Å, C), Val62(2.9 Å, C), Trp59(2.6 Å, C), Ser647(2.3 Å, A), Tyr597(2.3 Å ,A).<br>**Hydrophobic:** Phe681(4.0 Å, A), Phe60(3.0 Å, C), Met657(3.3 Å, A), Trp564(3.7 Å, A), Ile563(4.2 Å, A), Leu615(3.8 Å, A), Tyr597(4.0 Å, A).<br>**Stacking:** Phe60(3.8 Å, C)
**33**	-12.6	**Hydrogen bond:** Thr729(2.8 Å, A), Trp1701(3.0 Å, C).<br>**Hydrophobic:** Tyr642(3.9 Å, A), Tyr599(3.7 Å, A), Trp564(3.7 Å, A), Met567(3.6 Å, A), Ile563(5.5 Å, A), Ile592(3.6 Å, A), Pro648(3.8 Å, A), Phe681(3.1 Å, A), Ile682(4.1 Å, A), Ile706(4.1 Å, A), Phe1702(3.5 Å, C).<br>**Stacking:** Thr646(5.0 Å, A)
**34**	-12.7	**Hydrogen bond:** Thr729(2.1 Å, A), Tyr642(3.4 Å, A), Ser647(3.5 Å, A), Trp1701(3.0 Å, C).<br>**Hydrophobic:** Tyr642(3.4 Å, A), Phe681(3.4 Å, A), Ile682 (4.3 Å, A), Ile706(4.1 Å, A), Ile592(4.0 Å, A), Tyr599(4.0 Å, A), Ile540(3.6 Å, A), Ile563(3.5 Å, A), Pro648(3.9 Å, A), Phe1702(3.5 Å, C)<br>**Stacking:** Thr646(4.5 Å, A)
**35**	-12.7	**Hydrogen bond:** Thr729(3.5 Å, A), Val1704(3.0 Å, C), Trp1701(3.0 Å, C), Ser647(3.3 Å, A).<br>**Hydrophobic:** Tyr642(3.9 Å, A), Phe681(3.6 Å, A), Tyr599(4.0 Å, A), Trp564(3.5 Å, A), Pro648(3.7 Å, A), Ile563(3.5 Å, A), Ile592(4.4 Å, A), Phe1702(4.1 Å, C)<br>**Stacking:** Thr646(4.8 Å, A)
**36**	-13.7	**Hydrogen bond:** Thr729(3.5 Å, A), Val1704(3.0 Å, C), Ser647(2.8 Å, A), Tyr597(3.2 Å, A).<br>**Hydrophobic:** Tyr642(3.9 Å, A), Tyr599(4.0 Å, A), Trp564(3.5 Å, A), Pro648(5.4 Å, A), Phe681(3.6 Å, A), Met657(3.7 Å, A), Phe1702(4.1 Å, C)<br>**Stacking:** Tyr597(3.5 Å, A) y Thr646(5.0 Å, A).
**37**	-12.9	**Hydrogen bond:** Thr729(3.1 Å, A), Trp1701(3.0 Å, C), Tyr642(3.1 Å, A), Tyr597(2.7 Å, A), Ser647 (2.1 Å, A).<br>**Hydrophobic:** Tyr599(3.9 Å, A), Trp564(5.9 Å, A), Pro648(3.8 Å, A), Phe681(3.3 Å, A), Ile682(4.2 Å, A), Ile706(4.0 Å, A), Phe1702(3.8 Å, C)

Distance and letter in parenthesis indicate the shortest distance between the aminoacid and ligand, while the letter corresponds to the monomer of BRI1-BAK1.Detalils of the ligand fragment and aminoacid interaction, see Figures S59-S63
